# Parameterization-induced uncertainties and impacts of crop management harmonization in a global gridded crop model ensemble

**DOI:** 10.1371/journal.pone.0221862

**Published:** 2019-09-16

**Authors:** Christian Folberth, Joshua Elliott, Christoph Müller, Juraj Balkovič, James Chryssanthacopoulos, Roberto C. Izaurralde, Curtis D. Jones, Nikolay Khabarov, Wenfeng Liu, Ashwan Reddy, Erwin Schmid, Rastislav Skalský, Hong Yang, Almut Arneth, Philippe Ciais, Delphine Deryng, Peter J. Lawrence, Stefan Olin, Thomas A. M. Pugh, Alex C. Ruane, Xuhui Wang

**Affiliations:** 1 International Institute for Applied Systems Analysis, Ecosystem Services and Management Program, Laxenburg, Austria; 2 University of Chicago and ANL Computation Institute, Chicago, Illinois, United States of America; 3 Columbia University, Center for Climate Systems Research, New York, New York, United States of America; 4 National Aeronautics and Space Administration Goddard Institute for Space Studies, New York, New York, United States of America; 5 Potsdam Institute for Climate Impact Research, Member of the Leibniz Association, Potsdam, Germany; 6 Comenius University in Bratislava, Department of Soil Science, Bratislava, Slovak Republic; 7 University of Maryland, Department of Geographical Sciences, College Park, Maryland, United States of America; 8 Texas A&M University, Texas AgriLife Research and Extension, Temple, Texas, United States of America; 9 Eawag, Swiss Federal Institute of Aquatic Science and Technology, Duebendorf, Switzerland; 10 University of Natural Resources and Life Sciences, Institute for Sustainable Economic Development, Vienna, Austria; 11 Soil Science and Conservation Research Institute, National Agricultural and Food Centre, Bratislava, Slovak Republic; 12 Department of Environmental Sciences, University of Basel, Basel, Switzerland; 13 Karlsruhe Institute of Technology, IMK-IFU, Garmisch-Partenkirchen, Germany; 14 Laboratoire des Sciences du Climat et de l’Environnement, Gif-sur-Yvette, France; 15 Leibniz Centre for Agricultural Landscape Research (ZALF), Müncheberg, Germany; 16 IRI THESys, Humboldt University of Berlin, Berlin, Germany; 17 National Center for Atmospheric Research, Earth System Laboratory, Boulder, Colorado, United States of America; 18 Department of Physical Geography and Ecosystem Science, Lund University, Lund, Sweden; 19 School of Geography, Earth & Environmental Sciences, University of Birmingham, Edgbaston, Birmingham, United Kingdom; 20 Birmingham Institute of Forest Research, University of Birmingham, Edgbaston, Birmingham, United Kingdom; 21 Peking University, Sino-French Institute of Earth System Sciences, Beijing, China; Universidad de Murcia, SPAIN

## Abstract

Global gridded crop models (GGCMs) combine agronomic or plant growth models with gridded spatial input data to estimate spatially explicit crop yields and agricultural externalities at the global scale. Differences in GGCM outputs arise from the use of different biophysical models, setups, and input data. GGCM ensembles are frequently employed to bracket uncertainties in impact studies without investigating the causes of divergence in outputs. This study explores differences in maize yield estimates from five GGCMs based on the public domain field-scale model Environmental Policy Integrated Climate (EPIC) that participate in the AgMIP Global Gridded Crop Model Intercomparison initiative. Albeit using the same crop model, the GGCMs differ in model version, input data, management assumptions, parameterization, and selection of subroutines affecting crop yield estimates via cultivar distributions, soil attributes, and hydrology among others. The analyses reveal inter-annual yield variability and absolute yield levels in the EPIC-based GGCMs to be highly sensitive to soil parameterization and crop management. All GGCMs show an intermediate performance in reproducing reported yields with a higher skill if a static soil profile is assumed or sufficient plant nutrients are supplied. An in-depth comparison of setup domains for two EPIC-based GGCMs shows that GGCM performance and plant stress responses depend substantially on soil parameters and soil process parameterization, i.e. hydrology and nutrient turnover, indicating that these often neglected domains deserve more scrutiny. For agricultural impact assessments, employing a GGCM ensemble with its widely varying assumptions in setups appears the best solution for coping with uncertainties from lack of comprehensive global data on crop management, cultivar distributions and coefficients for agro-environmental processes. However, the underlying assumptions require systematic specifications to cover representative agricultural systems and environmental conditions. Furthermore, the interlinkage of parameter sensitivity from various domains such as soil parameters, nutrient turnover coefficients, and cultivar specifications highlights that global sensitivity analyses and calibration need to be performed in an integrated manner to avoid bias resulting from disregarded core model domains. Finally, relating evaluations of the EPIC-based GGCMs to a wider ensemble based on individual core models shows that structural differences outweigh in general differences in configurations of GGCMs based on the same model, and that the ensemble mean gains higher skill from the inclusion of structurally different GGCMs. Although the members of the wider ensemble herein do not consider crop-soil-management interactions, their sensitivity to nutrient supply indicates that findings for the EPIC-based sub-ensemble will likely become relevant for other GGCMs with the progressing inclusion of such processes.

## Introduction

Over the past decade, global gridded crop models (GGCMs) evolved to become major tools for agricultural climate change impact assessments [1–8]. They are also employed for studies on agricultural externalities [9–13] and provide key data for land use change and agro-economic models [14–17]. Typically, GGCMs are combinations of (a) a core model that estimates crop yields and externalities of crop production for a given set of input data and (b) a model framework that processes specified input data and runs the core model over large regions or the globe based on computational interfaces and georeferenced data from earth observations, statistical databases, or modelers’ assumptions. Most often, core models are based either on ecosystems models adapted for representing cropping systems or on field-scale crop models with varying level of detail in agro-environmental or management processes.

Despite their wide use and substantial deviations among studies based on single GGCMs (e.g. [2,11]), there is little insight regarding actual drivers behind these uncertainties, which can be grouped into simulated processes, process algorithms, input data, and parameterization including management assumptions. Field-scale crop models themselves have been subject to a wide range of uncertainty analyses ranging from crop yield and biomass performance [18] including in-season dynamics [19] to soil organic matter (SOM) spin-up [20], static or transient soil handling [21,22], and trial management [23]. Since the inception of the Agricultural Model Intercomparison and Improvement Project (AgMIP, [24]) these experiments are frequently carried out for crop model ensembles.

Past uncertainty analyses for GGCMs or large-scale crop models in contrast have mostly addressed GGCMs’ sensitivity to input data from different sources for climate (e.g. [2,11,25]) and soil (e.g. [26–28]), spatial resolution of input data (e.g. [29,30]), and deviations in outputs from different core models within the same framework ([28,30]). More recently, also core model subroutines have come under scrutiny, for example PET estimation methods [31], temperature response functions [32], and the parameterization of soil processes relating to land degradation at the regional scale [33]. In a recent GGCM ensemble study, Müller et al. [7] attributed shares of overall deviations in ensemble climate impact projections and found that the selection of GGCMs contributed the majority of overall uncertainty, at least when the effects of CO_2_ fertilization are accounted for.

Most prior studies targeting sources of uncertainty, however, have been performed for single GGCMs and typically for one singled out core model component. This provides thorough understanding of the considered components and their importance within a given GGCM and setup. Yet, it has previously not been investigated how assumptions on parameterizations and management–for which at present only limited data exist at the global scale—vary across GGCMs developed by different research groups, how single domains of GGCM setups interact, and how these translate into differences in GGCM outputs and performance. We present here a first evaluation of drivers in differences among yield estimates produced by a GGCM ensemble with a focus on setup configurations. Simulations were performed within AgMIP’s Global Gridded Crop Model Intercomparison (GGCMI) initiative phase 1 [34] in which GGCMs have been forced with their default or partly harmonized input data. The ensemble considered herein consists of 12 members (Table A in S1 File), five of which are based on the field-scale model Environmental Policy Integrated Climate (EPIC), while the remainder has unique core models. This allows for an in-depth evaluation how setup-related uncertainties translate into differences in GGCM outputs within the EPIC-based sub-ensemble and to assess how this sub-ensemble compares to the wider ensemble.

While other ensemble members have more complex routines for plant growth and yield formation processes (Table A in S1 File; [23]), EPIC presently stands out in its detailed representation of soil processes including organic matter and nutrient cycling, hydrology, and erosion as well as impacts of tillage on soil properties, besides being the only core model in GGCMI phase 1 used in multiple GGCMs. EPIC provides options for tracking changes in soil properties transiently or to reinitialize soils each year. This enables to account for temporal variations in soil quality or to focus on plant growth process with limited impact of dynamics in soil properties. Both options are employed in the ensemble based on modelers’ preferences. Further differences arise from the selection of subroutines for hydrologic processes, cultivar distributions, management assumptions, and process parameterization.

In most of the ensemble’s other GGCMs, detailed soil representations are absent as of now or soils are reinitialized annually. Still, few ensemble members such as APSIM- and DSSAT-based GGCMs [35–38] consider some of them in principle. Others have implemented transient dynamic soil routines and tillage operations affecting these in more recent versions not include in this experiment, as is the case for LPJmL and LPJ-GUESS [39–41]. Detailed soil processes are being implemented in other branches of ORCHIDEE (e.g. [42]) from which they may be transferred to the crop version. Beyond the present ensemble, various gridded crop models with detailed crop-soil-management interactions based on field-scale core models such as DNDC, Expert-N, MONICA, or STICS have been developed for regional studies (e.g. [43]), to which the same uncertainties apply.

This renders the present ensemble timely for the evaluation of differences in GGCM setups including the parameterization of agro-environmental processes and management assumptions, which we hypothesize to greatly affect GGCM outputs and performance. Furthermore, it allows to assess how such differences in setups relate to differences among core model formulations that entail substantial differences from inclusion and conceptualization to implementation of plant growth and agro-environmental processes. Through focusing the analysis on a single core model EPIC, it is possible to concentrate on the effects of different plausible setups whilst holding model structure constant, allowing comparison with a wider ensemble where both setup and structure vary. The knowledge gained from the analyses can inform both modelers and the wider scientific community, making use of the publicly available data (e.g. [44–48]), about the magnitude of process- and parameterization-induced uncertainty across highly distinct setups and the importance of specific setup domains.

The overarching aim of the study is to evaluate sources of uncertainty in maize yield estimates among the five EPIC-based GGCMs and to compare differences within this sub-ensemble to the wider ensemble. Specifically, the objectives are to

- identify key assumptions and setup components that drive differences in yield estimates,
- derive priorities for further improvements in GGCM input data and harmonization, and
- assess how findings for the sub-ensemble relate to a wider ensemble of different core models.

Complementary, a detailed evaluation is conducted for two EPIC-based GGCMs by step-wise introducing aggregated setup domains from one into the other in order to examine the importance of cultivar setups, organic matter turn-over and nutrient cycling, hydrologic parameterization, soil parameterization and handling, and crop management.

## Methods and data

### Global gridded crop models participating in this study

A total of 14 GGCMs contributed simulation outputs to GGCMI phase 1 [34], 12 of which provided outputs with harmonized input data (see next section). Five of the GGCMs (EPIC-BOKU, EPIC-IIASA, EPIC-TAMU, GEPIC, and PEPIC) are based on the field-scale model EPIC, while the remainder differs in core plant growth models. This study focuses on the five EPIC-based GGCMs, while the seven non-EPIC-based GGCMs are included in the Supplementary Information and Discussion to put the EPIC ensemble in a wider context, one of them only in the evaluation of model intercorrelation. Table A in S1 File provides an overview of key GGCM characteristics concerning plant growth, yield formation, and soil processes. More detailed information are provided on the website of ISI-MIP (http://www.isimip.org) and in Müller et al. [49,50]. In the further text, the term “model” refers to the core model routines and hence the structural differences, and the term “GGCM” to the global crop model framework, which may—as in the case of the EPIC-based GGCMs—only differ in setup and configuration.

### Crop management scenarios

Six crop management scenarios (Table 1) were simulated to allow for quantifying differences among GGCMs from assumptions on management. Three different harmonization setups on growing season (planting and harvest dates) and nutrient supply (default, fully harmonized, harmonized & sufficient nutrients) were combined with two water management scenarios: rainfed only and sufficiently irrigated using automatic irrigation scheduling based on water deficit. Parameterizations—except for the cumulative temperature requirement to reach maturity (see next section), which were adjusted according to the growing season setup of each management scenario—were not altered among scenarios to allow for evaluating the impacts of data harmonization alone.

**Table 1:** Crop management scenarios based on Elliott et al. [34]. The default setup represents each modelling group’s own assumptions, input data and management parameters. The harmonized scenarios use the same growing season data [51] and the same annual application rates for N and P [52] (fullharm) or sufficient nutrient supply (harm-suffN) to avoid nutrient-related plant growth limitations. See Fig E, panel a,b in S1 File for maps of harmonized N and P application rates.

Name	Abbreviation	Irrigation vol. [mm]	N [kg ha^-1^]	P [kg ha^-1^]	Growing season dates
Default, irrigated	default	sufficient	individual^1)^	individual^1)^	individual^1)^
Default, rainfed		-	individual^1)^	individual^1)^	individual^1)^
Fully harmonized, irrigated	fullharm	sufficient	harmon.^2)^	harmon.^2)^	harmon.^3)^
Fully harmonized, rainfed		-	harmon.^2)^	harmon.^2)^	harmon.^3)^
Harmonized & suff. nutrients, irrig.	harm-suffN	sufficient	sufficient	sufficient	harmon.^3)^
Harmonized & suff. nutrients, rainfed		-	sufficient	sufficient	harmon.^3)^

^1)^ Based on each research group’s assumptions and data

^2)^ Harmonized fertilizer application rates based on Mueller et al. [52] processed as described in Elliott et al. [34]

^3)^ Harmonized growing season data based on Sacks et al. [51] with gap filling as described in Elliott et al. [34]

The default scenario represents each research group’s assumptions on annual fertilizer application rates and growing seasons (see Text C in S1 File for EPIC-based GGCMs). It serves for evaluating differences among GGCMs if only climate data are harmonized. The fully harmonized (fullharm) setup allows for identifying remaining differences if annual nutrient application rates and growing seasons are harmonized using the input data described below. The fully harmonized setup with sufficient nutrient application (harm-suffN, referred to as harmnon in the simulation protocol [34]) aims to virtually eliminate plant nutrient deficits and consequently impacts of soil nutrient dynamics. This minimizes differences among GGCMs resulting from the setup of fertilizer application and soil nutrient cycling. As EPIC-TAMU was first setup in the course of this project, its default and fullharm setups are identical. To allow for evaluating the effect of harmonization from the default to the fullharm setups for the other EPIC-based GGCMs, supplementary results are shown with exclusion of EPIC-TAMU. This is also the case for LPJmL and LPJ-GUESS of the wider ensemble included in supplementary evaluations.

### The Environmental Policy Integrated Climate (EPIC) model

The EPIC model was first developed in the 1980s to assess the impacts of soil management on crop yields [53]. It has since been updated frequently to cover e.g. effects of elevated atmospheric CO_2_ concentration on plant growth [54], detailed soil organic matter cycling [55,56], and an extended number of crop types and cultivars [57,58] among others [59]. The presently publically available version is EPIC v.0810.

EPIC estimates potential biomass increase on a daily time-step based on light interception and conversion of CO_2_ to biomass. Plant growth and phenology are calculated based on the daily accumulation of heat units. Potential biomass increase is constrained by water and nutrient (nitrogen (N) and phosphorus (P)) deficits, adverse temperature, and aeration stress. On each day of the crop growth period, the potential biomass gain is adjusted by the major plant growth-regulating factor to obtain the actual biomass increment. Hence, only one stress factor limits biomass accumulation on a given day. Root growth can be limited by soil strength, adverse soil temperature, and aluminum toxicity. At maturity, the model calculates crop yield based on above ground biomass and an actual harvest index HI_a_, which is estimated within a range given by potential HI (HI_max_) and minimum HI under water stress (HI_min_).

Besides plant growth and yield formation, EPIC estimates a wide range of environmental processes, for example wind and water erosion rates, turnover and partitioning of organic matter (OM) based on the CENTURY model [55,60], mineral N and P cycling, evapotranspiration (ET), fluxes of selected gases, and soil hydrologic processes. All of these have feedbacks on plant growth, mainly through nutrient and water availability. EPIC has one central plant growth module, but provides various subroutines for calculating several of the externalities, e.g. six methods for water erosion estimation, eleven methods for estimating field capacity (FC) and wilting point (WP) including static input of own estimates or data, and five options for potential evapotranspiration (PET) among others. While this allows for adjusting the model to site conditions for which one method may be more appropriate than the other, it introduces another dimension of uncertainty besides the numeric parameterization of processes itself. Further information on relevant subroutines are provided in Text A in S1 File.

### Parameterizations of the EPIC-based global gridded crop models

#### Selection of global parameters

All EPIC-based GGCMs except EPIC-TAMU use EPIC v.0810 as the core model. EPIC-TAMU uses the experimental version v.1102, which has additional routines mainly for OM and nutrient cycling, but the same plant growth module. As shown in Text B (S1 File), EPIC v.1102 produces virtually identical outputs in high-input regions but shows differences in low-input agriculture where nutrient cycling has larger impacts on plant growth. It may hence be considered another configuration of EPIC in this context.

All EPIC-based GGCMs have been applied in prior studies except EPIC-TAMU, which has first been set up in the course of this project. Based on prior applications or modellers’ parameter estimates suitable for global simulations, the EPIC-GGCMs differ substantially in their parameterization and selection of subroutines. E.g. GEPIC has earlier been set up for reproducing small-holder agriculture in sub-Saharan Africa [61], relying partly on parameters calibrated in West Africa [58] whereas EPIC-IIASA has frequently been applied in high-input regions such as the EU [62] or China [63]. Yet, modelling purposes and target regions may change over time with ongoing research and parameterizations herein are accordingly results of both earlier research and modellers’ assumptions on globally representative settings. Outlines of EPIC-based GGCM setups, purposes, and prior applications are provided in Text C (S1 File). Table 2 gives an overview of the setups and parameterizations grouped by hydrology, soil degradation, OM and nutrient cycling, crop management, and crop growth apart from cultivar definitions, which are described in the subsequent section.

**Table 2:** Differences in parameters and choice of subroutines for the participating EPIC-based GGCMs. A dash indicates that the parameter is not relevant for the respective GGCM due to selection of subroutines. A brief explanation of parameters is provided in Table B in S1 File.

No	Parameter	EPIC-BOKU	EPIC-IIASA	EPIC-TAMU	GEPIC	PEPIC
**Hydrology**						
1	PET estimation method^1)^	PM	HG	PM	HG	PM
2	Hargreaves exp. coefficient	-	0.6	-	0.5	-
3	Hargreaves linear coefficient	-	0.0023	-	0.0032	-
4	Soil evaporation-cover coefficient	0	0	0.15	0	0
5	Soil cover-temperature function^2)^	1,30 8,95	1,30 8,95	1,05 3,95	1,30 8,95	1,30 8,95
6	Soil evaporation coefficient	2.5	1.5	2.5	2.5	1.5
7	Soil evaporation-depth function^2)^	10,50 100,95	10,50 100,95	10,70 100,95	10,50 100,95	10,50 100,95
8	Plant water use-soil water tension function^2)^	100,01 1000,90	100,01 1000,90	500,01 1500,50	100,01 1000,90	100,01 1000,90
9	FC, WP, and K_sat_ estimation^3)^	Rawls	static	Rawls	Rawls	Rawls
10	Soil variable dependence of CN^4)^	SMI	depth	depth	SMI	SMI
11	CN number index coefficient	1.5	1.2	1	0.5	1
12	CN coefficient for standing dead residue	0.0	0.0	0.3	0.2	0.0
**Soil degradation**						
13	Wind erosion considered^5)^	no	no	yes	yes	yes
14	Water erosion considered ^5)^	no	no	no	yes	yes
15	Water erosion conservation practice^6)^	-	-	-	0.5	1.0
16	Water erosion estimation method^7)^	-	-	-	MUSS	RUSL2
17	Field length for wind erosion	-	2.00	1.00	1.24	2.00
18	Field width for wind erosion	-	2.00	1.00	0.62	2.00
19	Soil profile handling (static/dynamic)^8)^	stat.	stat.	dyn.	dyn.	dyn.
20	Simulation continuity (transient/decadal)^9)^	trans.	trans.	trans.	dec.	trans.
**Organic matter and nutrient cycling**						
21	Denitrification method^10)^	EPIC	CI	AK	AK	AK
22	Microbial decay rate	1.0	0.8	1.0	1.0	1.0
23	Slow to passive humus coefficient	0.05	0.05	0.003	0.05	0.05
24	Oxygen content-soil depth function^2)^	200,05 500,90	400,05 600,90	200,05 500,90	200,05 500,90	200,05 500,90
25	Oxygen coefficient for microbial activity	0.90	0.99	0.80	0.90	0.90
26	N volatilization coefficient	0.005	0.700	0.030	0.005	0.300
**Crop management**						
27	Automatic irrigation trigger	0.90	0.80	0.99	0.90	0.90
28	Maximum single water application [mm]	50	500	100	1000	500
29	Automatic fertilizer application trigger^11)^	0.90	0.80	0.99	0.90	-
**Crop growth**						
30	Coefficient allocating root growth	0.5	0.5	0.7	0.5	0.5
31	Coefficient for root growth dist. by depth	10	10	7	10	10
32	Root growth stress considered	no	no	yes	no	no
33	Fraction of growing season from which HI_min_ affects yield formation	0.50	0.50	0.45	0.50	0.50

^1)^ PM: Penman-Monteith; HG: Hargreaves

^2)^ Parameters 5,7, 8, and 24 are X and Y values (separated by commas) for two points (upper and lower pairs) defining the shape of sigmoid functions

^3)^ Field capacity (FC) and wilting point (WP) can be estimated by 11 different methods or be an input in soil files. Saturated hydraulic conductivity (K_sat_) can be estimated according to Rawls method or be input. For EPIC-IIASA these parameters were estimated based on the ROSETTA model as described in Text C (S1 File).

^4)^ Describes the dependence of curve number (CN) estimation on soil moisture, which can be based on five methods, among them soil moisture gradient with profile depth or calculation of a daily soil moisture index (SMI)

^5)^ Water and wind erosion can be turned on or off and water erosion is estimated by different methods (see below)

^6)^ Water erosion rates are lowered by the given fraction (0 corresponds to virtually eliminated water erosion, 1 to no erosion control)

^7)^ MUSS: Modified Universal Soil Loss Equation for Small Watersheds; RUSL2: Modified Revised Universal Soil Loss Equation

^8)^ Static: annual re-initialization of soil profile, except water content and mineral nutrients; dynamic: transient updating of soil parameters throughout simulation

^9)^ GEPIC is run separately for each decade as described in Text C (S1 File)

^10)^ EPIC: original EPIC method [53]; CI: Cesar Izaurralde method [56]; AK: Armen Kemanian method (unpublished)

^11)^ The auto-fertilizer and irrigation triggers define at which stress level fertilizer or water are being applied. E.g., a value of 0.8 for the auto-fertilizer trigger implies that fertilizer is applied on a given day if potential biomass production would be limited by >20%. PEPIC employs rigid timing of N fertilizer application and has accordingly no threshold.

Numbers in parentheses below refer to column “No” in the table. As there are interactions among core model processes such as hydrology and OM cycling, this grouping is tentative and partly owed to the model structure. Concerning the choice of major subroutines, three GGCMs use Penman-Monteith (PM; [64]) for PET estimation (1) and two Hargreaves (HG; [65]) in different parameterizations. Only EPIC-IIASA uses prior estimated FC and WP parameters (9) while all other EPIC-based GGCMs estimate these parameters using Rawls method online ([66]). Water erosion (14) is considered in two of the GGCMs (and wind erosion in an additional one) with deviations in estimation method (16) and scaling of sediment yield (15). Three EPIC-GGCMs have a dynamic soil profile (19) with transient updating of profile depth, texture, OM, nutrient pools, and hydrology. In the two GGCMs with static soil profiles, soil texture and OM are re-initialized at the beginning of each year, but not mineral nutrient pools and soil humidity. All GGCMs are run transiently (20), except for GEPIC, which is run for each decade separately with a spin-up of 30 years (see Text C in S1 File). All three methods available for estimating denitrification (21) are used in the EPIC-based ensemble. Numeric parameters agree in some cases among GGCMs, especially if default parameter values have been selected (e.g. microbial decay rate (22)), but differ in several cases among four to five EPIC-GGCMs as is the case for the N volatilization coefficient (26). Different values have also been selected for defining irrigation water and fertilizer application strategies (27–29), and EPIC-TAMU differs in addition from the other GGCMs in the parameterization of selected plant growth process (30–33).

### Geographic distributions and parameterization of maize cultivars

**Fig 1:**
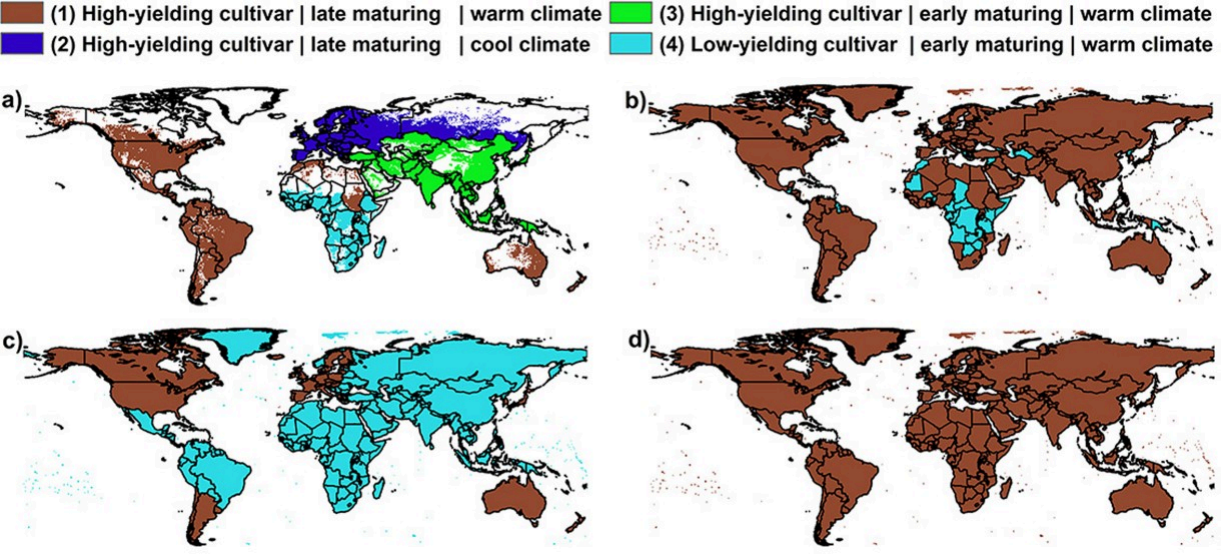
Distributions of maize cultivars in the EPIC-based GGCMs. (a) EPIC-IIASA, (b) EPIC-TAMU, (c) GEPIC and PEPIC, and (d) EPIC-BOKU. Differences in the parameterization of each cultivar are provided in Table D in S1 File. Numbers in parentheses (1–4) are used throughout the text to refer to the cultivars.

Differences in parameterizations of crop cultivars are evaluated here based on the parameters HI_min_, HI_max_, and optimal temperature ranges only. The latter is defined as the base and maximum temperatures for plant growth (see Text A, i.e. eq. S1 and S5, in S1 File). Although potential heat units (PHU), the temperature sum to reach maturity (Text A in S1 File), are an important cultivar characteristic as well, they are typically prescribed in GGCMs by growing season input data and long-term climate in each grid cell in order to meet reported sowing and harvest dates [61]. Between one and four different maize cultivars were planted within each EPIC-GGCM (Fig 1; Table D in S1 File). EPIC-IIASA uses four cultivars in its default setup (Fig 1A) that are attributed to major world regions based on climatic and economic characteristics. In the harmonized setup scenarios, cultivars 1 and 3 were merged as growing season length was defined according to common input data sets. EPIC-TAMU (Fig 1B) plants high- and low-yielding varieties. The latter is assigned to countries in which maize yields have stagnated or decreased within the past decades according to Ray et al. [67]. The high-yielding variety is assigned to all other regions. The same two maize cultivars were distributed in GEPIC and PEPIC (Fig 1C) based on the human development index (HDI). The high-yielding variety is planted in all countries with HDI≥80, which corresponds to “very high development” according to UN classification [68]. EPIC-BOKU used the high-yielding variety in all grid cells (Fig 1D).

### Common input data

Climate forcing data based on the WFDEI GPCC dataset [69] at a spatial resolution of 0.5° x 0.5° were provided by the ISI-MIP and GGCMI projects. The climate data are based on temperature and solar radiation from ERA-interim [70] and precipitation from GPCC [71]. All EPIC-based GGCMs used soil data from the ISRIC-WISE database [72] mapped to the Digital Soil Map of the World [73]. For EPIC-BOKU and EPIC-IIASA, the 5000 soil profiles had been reduced to the original 120 soil typologic units WISE is based on [74]. Soil hydraulic parameters not provided in the WISE database (FC, WP and saturated conductivity (K_S_)) were estimated for EPIC-IIASA using the ROSETTA model [75,76] and calculated endogenously in the other EPIC-based GGCMs using Rawls method (Table 2). Figure C in S1 File shows distributions of key soil parameters for both GGCMs.

For the harmonized runs, nutrient application rates for N and P were based on crop-specific mineral fertilizer application rates from [52] combined with N and P embedded in manure [77]. Harmonized planting dates and growing season lengths were based on Sacks et al. [51], complemented by gap filling with data from the MIRCA2000 dataset [78] and LPJmL [79]. Both datasets were provided by the GGCMI project [34]. Default runs were carried out using individual fertilizer and growing season data within each GGCM.

### Permutation of setup domains for GEPIC and EPIC-IIASA

**Fig 2:**
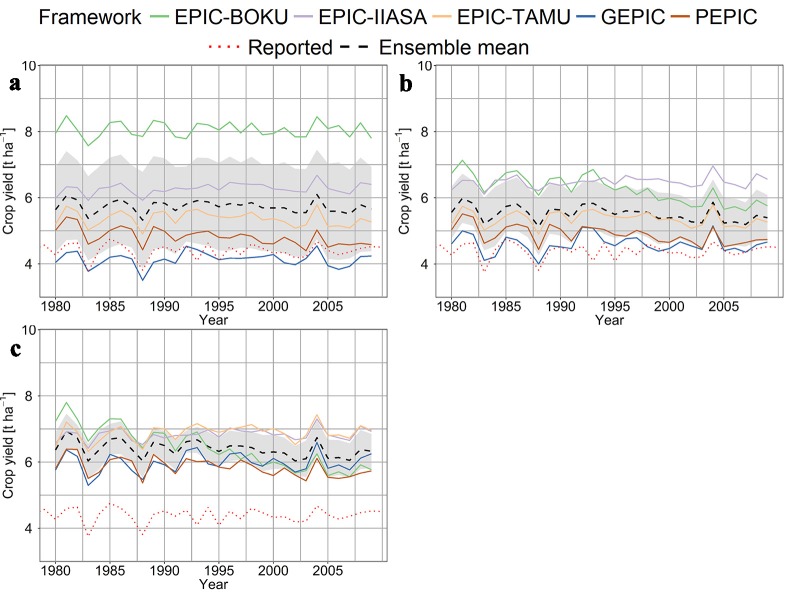
Global average area-weighted maize yield estimates of five EPIC-based GGCMs. (a) default, (b) fully harmonized (fullharm), and (c) fully harmonized with sufficient nutrient supply (harm-suffN) management scenario (Table 1). Reported yields are based on FAOSTAT **[85]** and have been detrended (see Methods). The black dashed line represents the ensemble mean. The grey ribbon shows the 95% confidence interval of the ensemble mean. Table F in S1 File hows statistical coefficients of yield trends over time and ME relative to FAO reported yields. Corresponding linear regressions are displayed in Fig F in S1 File.

To assess the importance of single data and parameterization domains within the EPIC-GGCMs, aggregated parameter domains of EPIC-IIASA were step-wise introduced into GEPIC. Parameters and routines were grouped into the six domains (Table 3) of cultivar distribution (Cult), soil parameterization (SoilD), soil handling (SoilP), nutrient turnover-related coefficients (CoeffN), hydrologic coefficients (CoeffW), and crop management (Manage). The two GGCMs were selected because yield simulations have no trend in time (Fig 2) and there are substantial differences in their setups (Table 2).

**Table 3:** Composition of aggregated setup domains the comparison of GEPIC in EPIC-IIASA in the fully harmonized (fullharm) scenario (Table 1). Numbers in the first column are used in selected figures to keep annotation short, otherwise the abbreviation is used. Numbers in column “Parameters considered” refer to those in Table 2. When referencing the setup domain parameterizations from each GGCM, e = EPIC-IIASA and g = GEPIC (e.g. eCult refers to cultivar setup of EPIC-IIASA).

No	Setup domain and abbreviation	Parameters considered	Effect in the EPIC model
**1**	**Cultivars** **(Cult)**	• see Fig 1 for distribution of cultivars and Table D in S1 File for differences in cultivar parameterization	• scaling of yields based on potential HI_max_ • higher sensitivity to water stress with lower HI_min_ • temperature ranges for optimal crop growth
**2**	**Soil parameterization** **(SoilD)**	• Table 2: 9 • differences in hydrologic soil group definitions • sum of bases and saturated conductivity only in EPIC-IIASA soil files • ten soil layers in EPIC-IIASA • five soil layers in GEPIC	• soil hydrology • nutrient cycling • little difference in basic soil properties (see Fig C in S1 File)
**3**	**Soil handling** **(SoilP)**	• Table 2: 13–20 • decadal runs with dynamic soil handling in gSoilP (Fig B in S1 File) fully transient runs with static soil profile in eSoilP	• carry-over effects in transient runs but re-initialization of soil texture, depth and OM for EPIC-IIASA setup • carry-over effects for all soil variables including losses from erosion transient for each decade with 30yr spin-up (see Text C in S1 File)
**4**	**Parameterization of organic matter and nutrient cycling** **(CoeffN)**	• Table 2: 21–26	• nutrient fate and availability • e.g. denitrification, microbial mineralization, partitioning to OM pools
**5**	**Parameterization of hydrologic processes** **(CoeffW)**	• Table 2: 2, 3, 6, 10, 11, 12	• PET estimation • runoff and percolation • plant water deficit • indirectly OM and nutrient cycling
**6**	**Crop management** **(Manage)**	• Table 2: 27–29 • a list of crop management operations in both GGCMs is provided in Table C in S1 File	• short- and long-term nutrient availability • surface roughness and soil erodibility • potential biomass estimation

GEPIC was run with all 64 (2^6^) resulting setup combinations using the land mask of EPIC-IIASA to ensure consistency. The evaluation focuses on rainfed yield estimates as these cover the whole range of uncertainty impacts. Magnitudes of plant growth stresses are included to analyze drivers behind different yield estimates. Benchmarking against reported yields at the country-level serves for quantifying the contribution of single setup domains to GGCM performance besides the GGCM’s sensitivity for a given setup domain in contrasting countries.

### Evaluation and reference data

#### Yield aggregation

Crop yields are compared and evaluated at the global, national, and grid level as well as aggregated to Koeppen-Geiger regions (Fig D in S1 File). Agreement among GGCMs is compared in relation to fertilizer application rates, mean annual precipitation (MAP), and cultivar distributions to identify drivers of deviations in yield estimates.

Global and national average yields (*YD*_*av*_) were calculated from simulated rainfed and irrigated yields in each grid cell and the respective rainfed and irrigated harvested areas obtained from the MIRCA2000 dataset [78], which provides rainfed and irrigated areas for various crops around the year 2000, according to

$$YD_{av,c}=\frac{\sum_{g=1}^{m}\left[YD_{i,g}\times HA_{i,g}+YD_{r,g}\times HA_{r,g}\right]}{\sum_{g=1}^{m}\left[HA_{i,g}+HA_{r,g}\right]}$$ (1)

where *YD*_*av*,*c*_ is the national average yield in country *c*, *YD*_*i*,*g*_ is yield under irrigated conditions in grid cell *g*, *YD*_*r*,*g*_ is yield under rainfed conditions in grid cell *g*, *HA*_*i*,*g*_ is irrigated area in grid cell *g*, and *HA*_*r*,*g*_ is rainfed area in each grid cell *g*, and *m* is the number of grid cells in country *c*. We acknowledge the uncertainty introduced from spatial aggregation [80] but as the focus is on a comparison among GGCMs, we consider this to be of minor importance here.

#### Metrics for GGCM agreement

The coefficient of variation (CV) [%] was used as a metric for absolute bias among yields averaged throughout the study period (CV_av_) as well as changes in inter-annual yield dynamics if GGCM setup components are introduced from GEPIC into EPIC-IIASA (CV_t_). The coefficient of variation is expressed as

$$CV=\frac{S}{\bar{X}}\times 100\%$$ (2)

where *S* is the standard deviation and $\bar{X}$ is the mean of yields throughout the evaluation period in each grid cell or globally aggregated. CV_av_ was calculated for the period 1980–2009 as the first simulation year 1979 did not have a complete growing season globally. CV_t_ was calculated the same way but after scaling the time series average to 1 in order to avoid bias caused by changes in the magnitude of yields.

The mean error (ME) was used in the same evaluations as a metric for absolute bias including the sign of change:

$$ME=\frac{\sum_{i=1}^{n}YD_{est}-YD_{ref}}{n}$$ (3)

where *YD*_*est*_ is the yield estimate, *YD*_*ref*_ is the reference yield, and *n* is the number of years considered. In the permutation of GGCM setup domains (Table 3) difference are evaluated in relative terms compared to the original EPIC-IIASA setup. ME hence corresponds there to the fraction of relative change [–].

To test the agreement in inter-annual yield variability among GGCMs, we used the time-series correlation [34,49] according to Pearson’s correlation coefficient *r* calculated for yield time-series in each grid cell pairwise among all GGCMs according to

$$r=\frac{\sum_{i=1}^{n}\left(x_{i}-\bar{x}\right)\left(y_{i}-\bar{y}\right)}{\sqrt{\sum_{i=1}^{n}{\left(x_{i}-\bar{x}\right)}^{2}}\sqrt{\sum_{i=1}^{n}{\left(y_{i}-\bar{y}\right)}^{2}}}$$ (4)

where *n* is the sample size, *x*_*i*‥*n*_ and *y*_*i*‥*n*_ are paired samples of yield estimates from two GGCMs, *x*_*i*_ and *y*_*i*_ are the *i*th elements of each total sample, and $\bar{x}$ and $\bar{y}$ are the sample means. As one of the EPIC-based GGCMs exhibited a substantial decline in yields after the first simulation years, the evaluation period was limited to 1980–1990 in the GGCM inter-comparison in order to avoid bias from unexpected model behavior later in the simulation period.

All evaluations were carried out with the statistics software R [81] using the packages ggplot2 [82], corrplot [83], and the heatmap.2 function of gplots [84] in a modified version from Müller et al. [49] for visualization.

#### Benchmark metrics for reproducing reported yields

Skills of the ensemble with respect to reproducing reported inter-annual yield variability and absolute yields have been assessed in detail in Müller et al. [49] across various sets of benchmark, land use, and climate data. Due to uncertainties inherent also in benchmark and land use data (see below), the performance evaluation herein does not aim at identifying an ensemble member performing optimally at global or regional scales. Rather, it serves for comparing skills of GGCMs in relation to differences in setups, i.e. the number of countries in which a specific GGCM shows high performance or how performance changes with permutation of model setup domains. Thereby, we focus on the two harmonized management scenarios fullharm and harm-suffN to ensure comparability in key input data.

Following the methodology of Müller et al. [49], we used time-series correlation coefficient r (Eq 4) between detrended national average simulated and reported yields as the main metric. Reported national and global average yields were obtained from FAOSTAT [85]. Detrending was performed in order to remove temporal trends in reported yields due to changes in technology and management by subtracting the 5-year moving mean [34,49]. As a reference, the detrended yields from FAOSTAT were multiplied by their mean of the period 1997–2003 for which fertilizer inputs are representative. The comparison of reported versus simulated global and national yields was performed for the complete simulation period 1980–2009. A significance threshold of p<0.1 (at approx. r>0.31) was selected for defining good performance compared to reported yields. The mean bias in absolute yield estimates from reported yields was measured as mean error (Eq 3).

The evaluation of GGCM performance itself is often limited by the quality of benchmark [49] and land-use data [80], characteristics of climate data (Ruane et al., in preparation), and representativeness of management data for a given region. Benchmarking itself is hence subject to substantial uncertainties and was here limited to major producers and other countries for which available benchmark and management data can be considered representative. These were selected based on whether (a) production and harvest area data had not been estimated by FAO and (b) harvested area did not fluctuate by >100% throughout the study period to account for the static cropland mask used in the aggregations.

## Results

### Effects of harmonization on global average maize yield estimates

If the EPIC-based GGCMs are run in their default setups, global average simulated maize yields differ by up to 124% annually (mean 95%) using the lowest estimate as a reference (Fig 2A; Table E in S1 File). This is mainly due to very high yield estimates from EPIC-BOKU of around 8 t ha^-1^, while the other EPIC-based GGCMs have yield estimates of around 4–6 t ha^-1^. The ranges decrease to 55% if harmonized planting dates and fertilizer application rates are used (Fig 2B) and further to 26% with sufficient nutrient supply (Fig 2C). Accordingly, the bias from reported yields varies greatly by GGCM and scenario with the largest bias in terms of ME for EPIC-BOKU in the default setup and the lowest for GEPIC in the fullharm scenario (Table F in S1 File). The mean bias is not constant over time, however, with significant negative trends in yield estimates for PEPIC in all setup scenarios, and for EPIC-BOKU in the fullharm and harm-suffN scenarios. EPIC-IIASA in contrast shows a slight positive trend in its default setup. Still, major inter-annual patterns agree among GGCMs and reported data reasonably well and the whole EPIC-based ensemble reproduces a reported peak in global average yield in 2004.

### Spatial differences in mean and inter-annual maize yield estimates

#### Deviations in long-term mean yields

**Fig 3:**
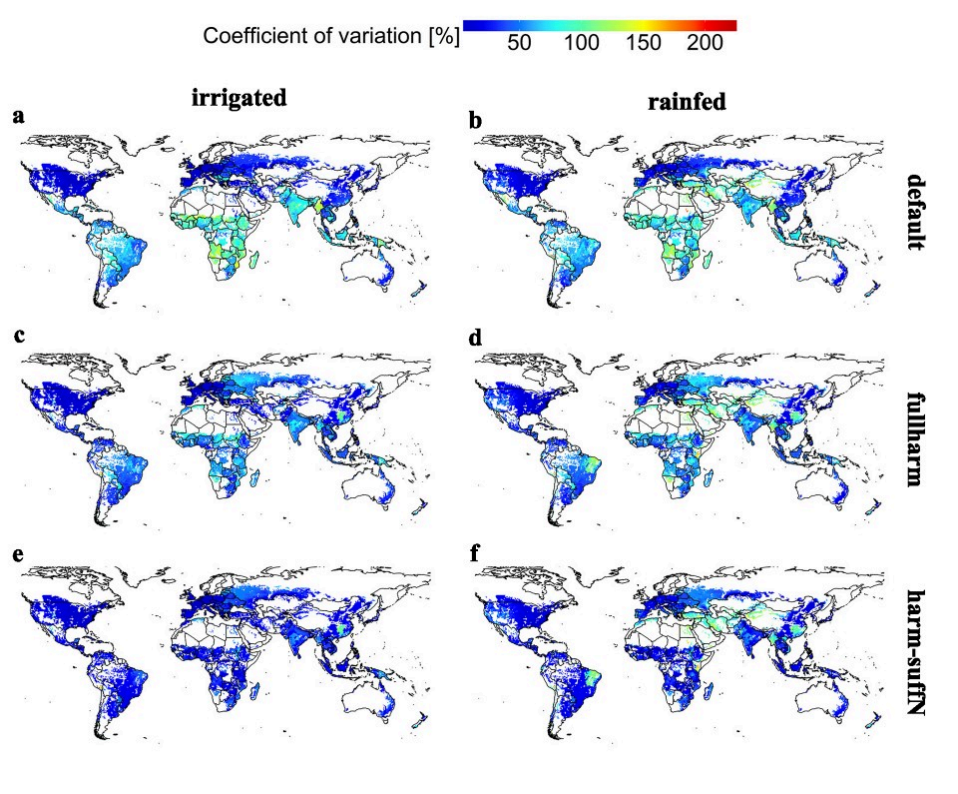
Coefficient of variation for long-term average maize yield estimates (CV_av_) among EPIC-based GGCMs. Panels reflect each of the six crop management scenarios defined in Table 1. Complementary maps without EPIC-TAMU, for which default and fullharm are identical, are provided in Fig G in S1 File.

Spatially, the deviation of maize yield estimates among the EPIC-GGCMs is largest with the default setups in tropical and arid regions (Fig D in S1 File) with CV_**av**_ of up to 224% and CV_**av**_ ≥ 44% in > 50% of all grid cells (Fig 3A and 3B; Table G in S1 File). The most distinct differences with CV_**av**_ > 100% were found in sub-Saharan Africa, South America, India, and Southeast Asia. The smallest differences occur in mid and high latitudes of both hemispheres, where (a) fertilizer inputs are at moderate or high levels (Fig E in S1 File), (b) most GGCMs plant the same high-yielding cultivar (Fig 1) and (c) the climatology usually defines a narrow growing season window limiting differences among GGCMs in planting date assumptions. Rainfed cultivation results in larger differences among GGCMs in (semi-)arid regions of Central and West Asia, the Western USA and Northeastern Brazil. If irrigation water is applied, differences increase in most parts of sub-Saharan Africa and Central India, but decrease in most of North and South America, Central Asia, and Europe.

Harmonizing fertilizer and growing season data reduces CV_av_ to ≤62% under rainfed and ≤54% under irrigated conditions in 75% of all grid cells (Fig 3C and 3D; Table G in S1 File). Spatial patterns remain similar to those found for the default managements, but CV_av_ also increases substantially in few regions after harmonization such as Western Russia or Southern China. The application of sufficient nutrients further reduces differences among EPIC-GGCMs (Fig 3E and 3F). Agreement improves especially in regions with low or moderate reported fertilizer application rates (see Fig E in S1 File) such as India, sub-Saharan Africa, and South America, but higher CV_av_ compared to the default setups remains in Western Russia and Southern China. Excluding EPIC-TAMU, for which missing default simulations have been replaced by the fullharm setup, from the analysis results in comparable spatial patterns (Fig G in S1 File), lower CV_av_ in the lower percentiles, and higher CV_av_ in the upper percentiles except for the maximum value (Table H in S1 File).

In the regions with increased CV_av_ in the harmonized setups, different mechanisms are at play. Selecting an administrative unit from each of the two regions (Fig I in S1 File) indicates that in the province Hunan (China), yields decrease uniformly for all EPIC-based GGCMs, except EPIC-TAMU which has identical default and fullharm setups, leaving the absolute difference constant but increasing the CV_av_ value. The fact that there is little difference between the fullharm and harm-suffN setups and higher yields in the default scenario indicates that the harmonized growing season, which refers to a side season at the end of the year with low precipitation and 129 days until harvest (not shown), drives the yield decrease. In the oblast Krasnodar (Russia), GGCM responses to harmonization are more diverse. GEPIC and PEPIC show a decrease in yields from default to fullharm followed by an increase with harm-suffN, indicating a partial impact of nutrient supply. Yet, for EPIC-IIASA, which simulates an adapted cultivar in this region (Fig 1A), yields increase continuously among scenarios. EPIC-BOKU in turn shows substantial yield reductions in the harmonized setups indicating that sufficient nutrient supply cannot offset yields in this GGCM.

#### Deviations in inter-annual yield dynamics

**Fig 4:**
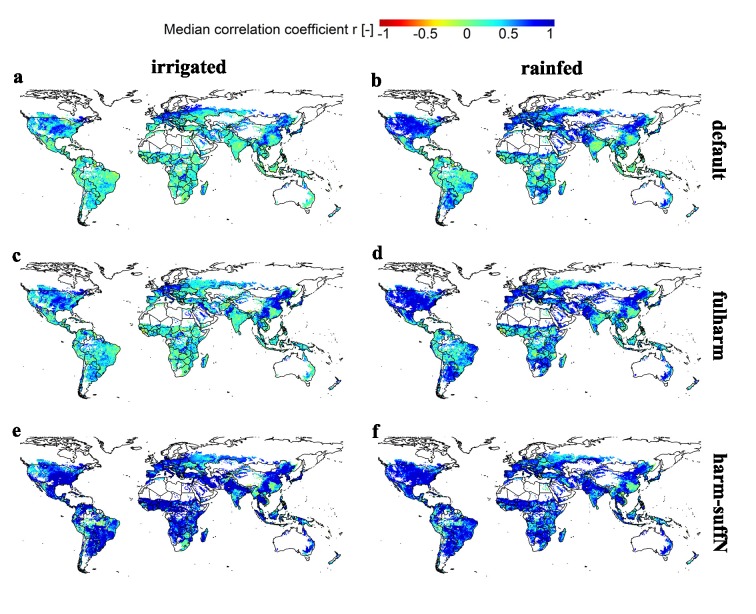
Median time-series correlation coefficient r for maize yield estimates among EPIC-based GGCMs. Panels reflect each of the six crop management scenarios defined in Table 1. Complementary maps without EPIC-TAMU, for which default and fullharm are identical, are provided in Fig K in S1 File.

In the default setup, the median time-series correlation coefficient r among the EPIC-based GGCMs is often around zero (Fig 4A and 4B; see also Fig M in S1 File), except for temperate and cold regions in case of sufficient irrigation and additionally in arid regions under rainfed conditions. Globally, still >40% of all grid cells have a median correlation that is statistically significant (r with at least p<0.1, Table I in S1 File) under rainfed conditions, but only 17% with sufficient irrigation. Harmonization provides often a slight improvement, foremost with a higher correlation in regions that already had a moderate agreement in the default setups (Fig 4C and 4D). Low agreement prevails especially in the tropics and along the Eurasian border, where the correlation partly decreases compared to default. With sufficient nutrient supply (Fig 4E and 4F), there is a significant correlation in 68% of grid cells under both irrigated and rainfed water supply, and a very high agreement at p<0.01 in 51% or 43%, respectively (Table I in S1 File). The largest deviations remain in the humid tropics of sub-Saharan Africa and South America, South(-East) Asia, and along the Eurasian border. Excluding EPIC-TAMU from the analysis results again in similar spatial patterns (Fig H in S1 File), but a substantially lower fraction of grid cells with a good median agreement occurs in the harm-suffN scenario (Table J in S1 File).

**Fig 5:**
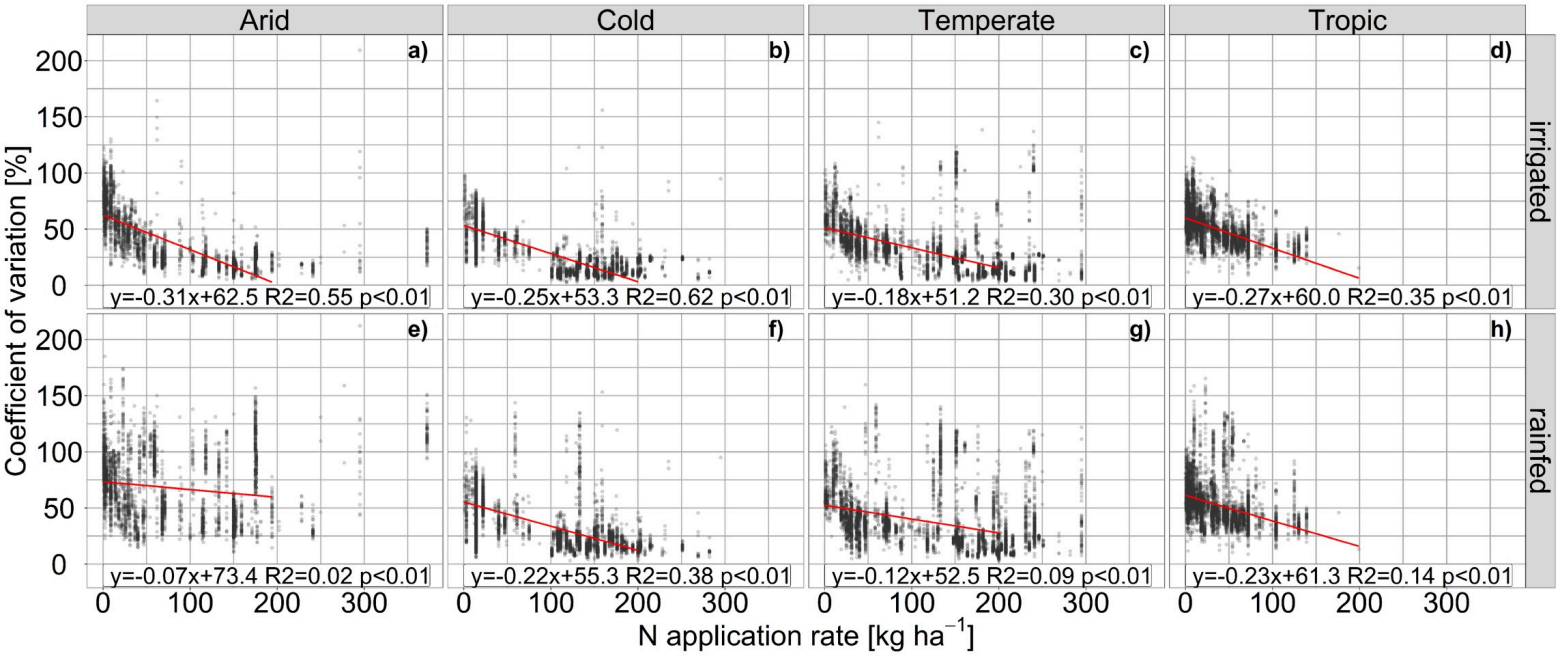
Coefficient of variation for maize yields among EPIC-based GGCMs compared to fertilizer application rates. Results are shown for the fully harmonized management scenario (fullharm) with sufficiently irrigated (a-d) or rainfed (e-h) water supply in each grid cell of four major climate regions. Linear regressions are limited to ≤200 kg N ha^-1^, which commonly corresponds to sufficient N supply [86].

**Impact of fertilizer supply on deviations in maize yield estimates.** As indicated by the cross-scenario analyses above, the bias among GGCMs in terms of CV_**av**_ is largely driven by crop nutrient supply. Accordingly, within the fullharm scenario CV_**av**_ is inversely correlated with the level of N fertilizer supply in most climate regions (Fig 5). Although linear regressions are highly significant in all climate region x water supply combinations, the explained variance is substantially higher under irrigated (Fig 5A–5D) than under rainfed conditions (Fig 5E–5H). CV_**av**_ is on average at about 60–85% in all climate regions at very low N application levels and highest in arid regions under irrigated conditions. It decreases on average to about 22% in arid and temperate and 17% in tropical and cold regions at applications rates above 200 kg N ha^**-1**^ yr^**-1**^. Rainfed cultivation substantially dampens the effect of nutrient application rates as a driver for differences among GGCMs in (semi-)arid climates and leaves larger deviations at moderate to high application rates also in other climate regions. The large deviation at fertilizer application rates >300 kg N ha^**-1**^ in arid regions under rainfed conditions (Fig 5E) is apparently caused by soil hydrology as indicated by the substantially lower deviation under irrigated conditions (Fig 5A).

**Fig 6:**
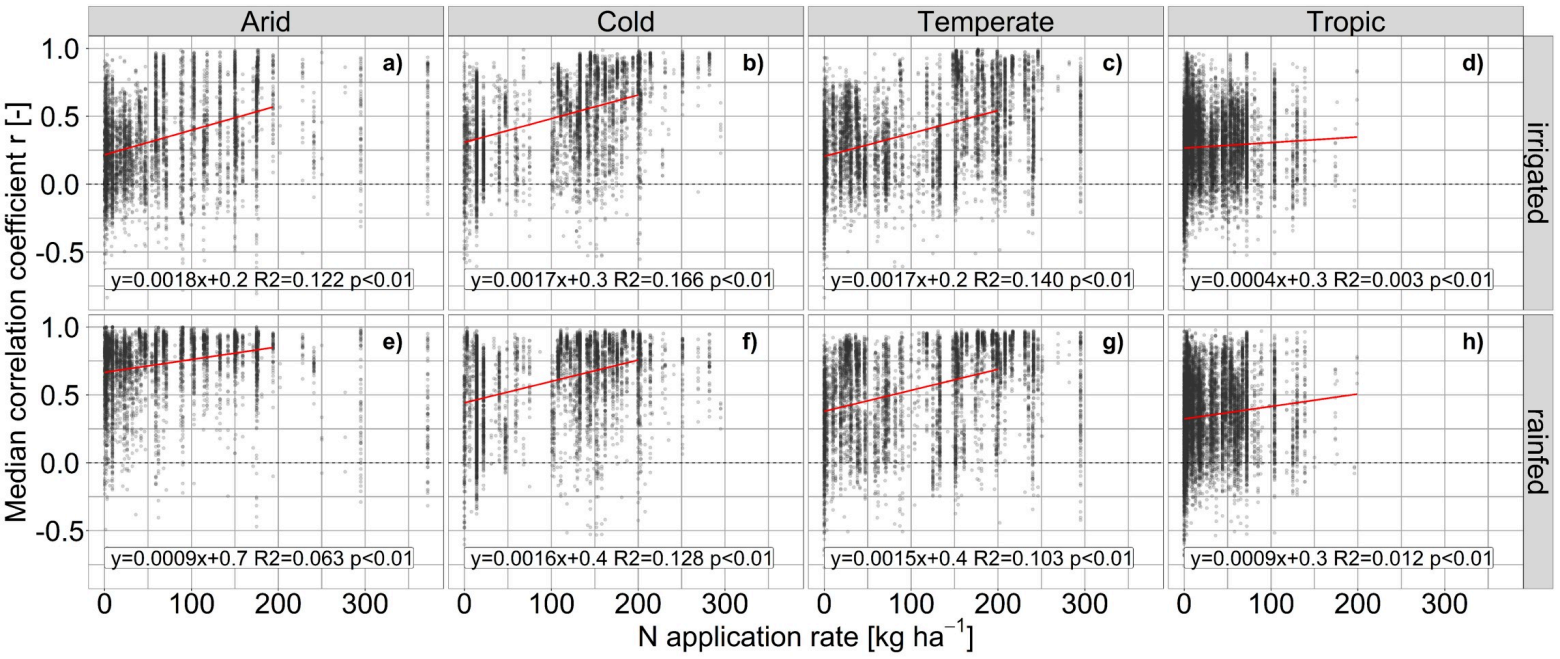
Median time-series correlation coefficient r for maize yields among EPIC-based GGCMs compared to fertilizer application rates. Results are shown for the fully harmonized management scenario (fullharm) with sufficiently irrigated (a-d) or rainfed (e-h) water supply in each grid cell of four major climate regions. Linear regressions are limited to ≤200 kg N ha^-1^, which commonly corresponds to sufficient N supply [86].

Similarly, the correlation among GGCMs increases with increasing fertilizer application rates (Fig 6A–6H) especially under irrigated conditions (Fig 6A–6D), but with comparably little explained variance. The highest impact of fertilizer application can be found in cold and temperate climates under irrigated conditions (Fig 6B and 6C). In arid regions under rainfed conditions, the correlation is often already high at low fertilizer application rates (Fig 6E) implying that climatic drivers dominate here the GGCM inter-correlation. In the tropics in contrast, where fertilizer application rates are commonly moderate to low, the correlation is at all application rates lower than in other regions (Fig 6D and 6H). Binning the fertilizer application rates (Fig J in S1 File) shows that there may rather be thresholds of application rates allowing for high correlation among GGCMs at least in arid, cold and temperate regions where a substantial increase can be found for the first two at 50–100 kg N ha^-1^ and for the latter at >150 kg N ha^-1^. Again, this occurs foremost with sufficient water supply (Fig J, panel a-d in S1 File).

**Impact of cultivar distributions on deviations in maize yield estimates.** Besides nutrient supply, an important driver for remaining differences in spatial mean yield estimates is the cultivar distribution. If all GGCMs plant one of the high-yielding cultivars 1 or 2, CV_**av**_ is typically lowest (Fig K in S1 File). The deviation increases for regions in which all four GGCMs that use Cultivar 4 plant this variety and is often 50–100% higher than the first option if none of the cultivars dominates. This applies to regions in which GEPIC and PEPIC but not EPIC-IIASA and EPIC-TAMU plant cultivar 4. The effect is stronger under rainfed than under irrigated conditions. Cultivar distributions can hence also explain some of the remaining differences in the harm-suffN scenario (Fig 2E and 2F), e.g. in Western Russia (see above).

The impact on the time-series correlation coefficient is less evident (Fig L in S1 File). Regardless of the water supply regime and cultivar definitions, the agreement is high in arid regions with lowest agreement if all GGCMs plant a high-yielding cultivar with irrigation. At overall lower agreement, the picture is similar in the tropics where it also applies to the mixed cultivar definitions. Cold and foremost temperate regions in contrast show a gradient in decreasing agreement among GGCMs from uniform planting of the high-yielding cultivar towards dominant low-yielding cultivar or mixed cultivar definitions.

### Impact of single setup domains on yield deviations

**Fig 7:**
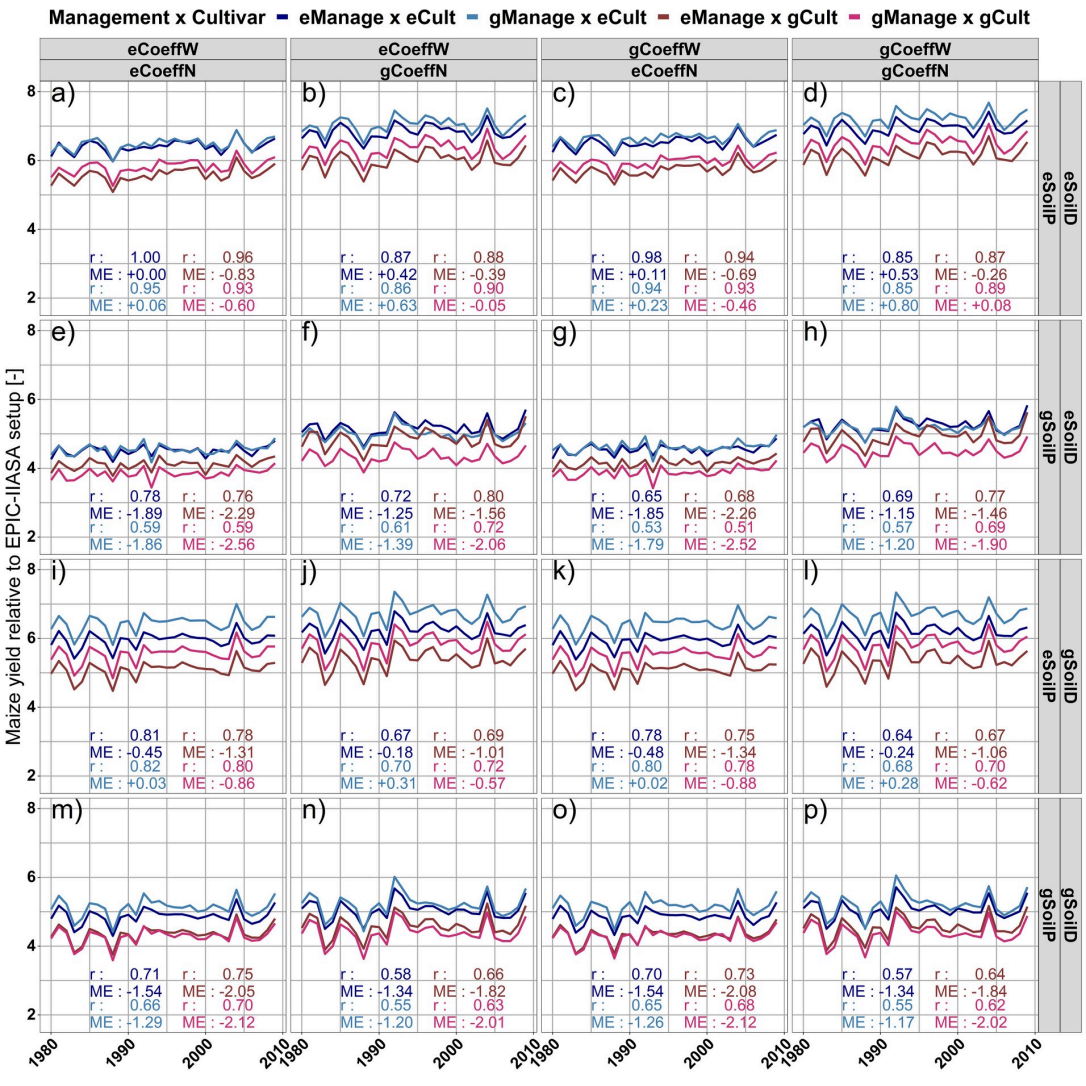
Relative difference in global average rainfed maize yields over a 29 year period for 64 setup combinations. Setup domains are introduced from GEPIC into the EPIC-IIASA setup (Table 3) and compared to the original EPIC-IIASA configuration. e = EPIC-IIASA, g = GEPIC, Cult = cultivar definition and distribution, SoilD = soil parameters, SoilP = spin-up and soil handling, CoeffN = organic matter and nutrient cycling coefficients, CoeffW = hydrologic coefficients, Manage = crop management. CV_t_ = coefficient of variation over time normalized to mean = 1. ME = mean error compared to the full EPIC-IIASA setup. Corresponding absolute yields are provided in Fig N in S1 File.

The further evaluation of differences in setup domains between EPIC-IIASA and GEPIC (Fig 7A–7P) focuses on the relative difference from the complete EPIC-IIASA setup (Fig 7A). Complementary magnitudes of plant stresses are provided in the Supplementary Information (Fig O in S1 File). They cannot be related to differences in yield estimates directly as their impact depends on estimates of potential biomass growth in the EPIC model (driven e.g. by growing season length, climate and management; see also Text A, S1 File) and the timing of the stress occurrence. They are hence addressed per panel but not among different managements. Selected examples for single grid cells are provided in Figs O and P in S1 File, maps of dominant stresses for contrasting setups in Fig R in S1 File.

If only the management is used from the GEPIC setup and everything else is set to the EPIC-IIASA setup, yields increase slightly compared to the full EPIC-IIASA setup (Fig 7A) despite an increase in phosphorus (P) and water (W) deficits (Fig O, panel a in S1 File) and show an increase in inter-annual yield variability in terms of CV_t_. This is caused by the narrower row spacing in GEPIC (Table C in S1 File), which increases the estimate of potential biomass (Text A in S1 File) often resulting in higher actual biomass estimates despite higher stress occurrence (see Fig P in S1 File for grid cell example). Replacing also the cultivars scales yields down and increases variability as GEPIC plants the low-yielding, drought-sensitive cultivar 4 in a larger number of countries (Fig 1A and 1C). Introducing the gCoeffN parameters into the setup (Fig 7B) increases yields in all cultivar x management combinations and affects inter-annual yield dynamics whereas nutrient-related stresses decrease (Fig O, panel b in S1 File) due to more rapid turnover of organic matter (Table 2; see Fig Q in S1 File for point level example of eCoeffN vs gCoeffN). The slight increase in temperature (T) stress is hence a secondary effect due to the stress handling in the EPIC model selecting only the major limiting factor for biomass production on a given day (see Methods). The gCoeffW parameters in turn import little change on yield variability but slightly scale yields up for each CoeffN parameterization (Fig 7C and 7D).

Replacing in a further step the static soil handling of EPIC-IIASA by the dynamic decadal runs of GEPIC (Fig 7E–7H) alters yield levels and inter-annual dynamics substantially with about 15% lower yields than in the corresponding eSoilP scenarios. Nutrient deficits become the dominant growth constraint, especially in combination with eCoeffN (Fig O, panel e,g in S1 File), which causes a slower release of nutrients from OM and higher volatilization of N. The higher P stress with gCoeffN (Fig O, panel f,h in S1 File) is often a secondary effect of high N availability early in the simulation that causes more rapid P mining form the soil in low-P input regions and a concomitant increase in P stress.

Introducing in addition the soil parameters of GEPIC gSoilD into the setup combinations (Fig 7I–7P) results in an increase in yield estimates and changes in inter-annual yield variability in all scenarios (Fig 7A–7H vs Fig 7I–7P). This is driven by decreases in N stress and increases in P stress if a static soil profile eSoilP is employed or if the dynamic soil handling gSoilP is combined with eCoeffN (Fig O, panel m,o in S1 File). The most significant difference between the soil parameterizations is in the estimation of hydraulic parameters field capacity (FC) and wilting point (WP) where EPIC-IIASA has typically higher values for the first and lower for the latter (Fig C in S1 File). Both parameters affect a wide range of processes in the EPIC model, among them the threshold for percolation of water and the optimal soil humidity for microbial processes (see Text A in S1 File). The gSoilD component hence allows for providing larger amounts of nutrients from OM as required soil humidity is reached earlier, but causes higher water stress as an effect of (a) lower water storage capacity and (b) higher model sensitivity to climate stresses caused by higher nutrient supply. In the combination of the static soil profile eSoilP and the parameter set gCoeffN (Fig 7J and 7L), nutrient stresses are virtually eliminated and yield estimates are foremost driven by climate (Fig O, panel d; Fig R, panel d in S1 File), potential biomass accumulation, and cultivar specification.

A correlation matrix of global area-weighted yields among all permutations (Fig S in S1 File) shows that the combination of eSoilD, gSoilP, and eCoeffN (Fig 7E and 7G) has the lowest agreement with the remainder of setups (Fig S in S1 File). In turn, the nutrient and OM turnover parameterizations and soil parameters of GEPIC (gCoeffN and gSoilD) as well as the static soil handling of EPIC-IIASA (eSoilP) render the GGCM resilient to changes in other setup domains (Fig T in S1 File), while the remaining setup domains show bimodal distributions and hence depend more strongly on interactions.

### Impacts of setups on GGCM performance

The EPIC-based GGCMs show a mixed performance in the fullharm setups (Table 4). GEPIC and PEPIC, notably the two GGCMs considering a dynamic soil profile and erosion (Table 2), exhibit relatively poor skills in terms of countries in which they have best performance. However, all EPIC-GGCMs have a good performance in about half or more of the countries considered. If sufficient nutrients are supplied, the numbers of countries in which each GGCM is best performing or has at least a high performance, converge to about ±2, except for PEPIC in the latter case.

**Table 4:** Numbers of countries (out of 99 for which benchmark data and GGCM outputs are available) in each harmonized setup scenario, in which each EPIC-based GGCMs has the highest (column “best”) performance compared against reported yields within the EPIC ensemble and all countries (column “all”) in which the correlation coefficient is significant at p<0.1 and positive.

Scenario	fullharm		harm-suffN	
**GGCM**	best	all	best	all
**EPIC-BOKU**	20	56	18	59
**EPIC-IIASA**	26	56	23	60
**GEPIC**	15	50	19	58
**EPIC-TAMU**	23	48	20	61
**PEPIC**	15	48	19	52

**Fig 8:**
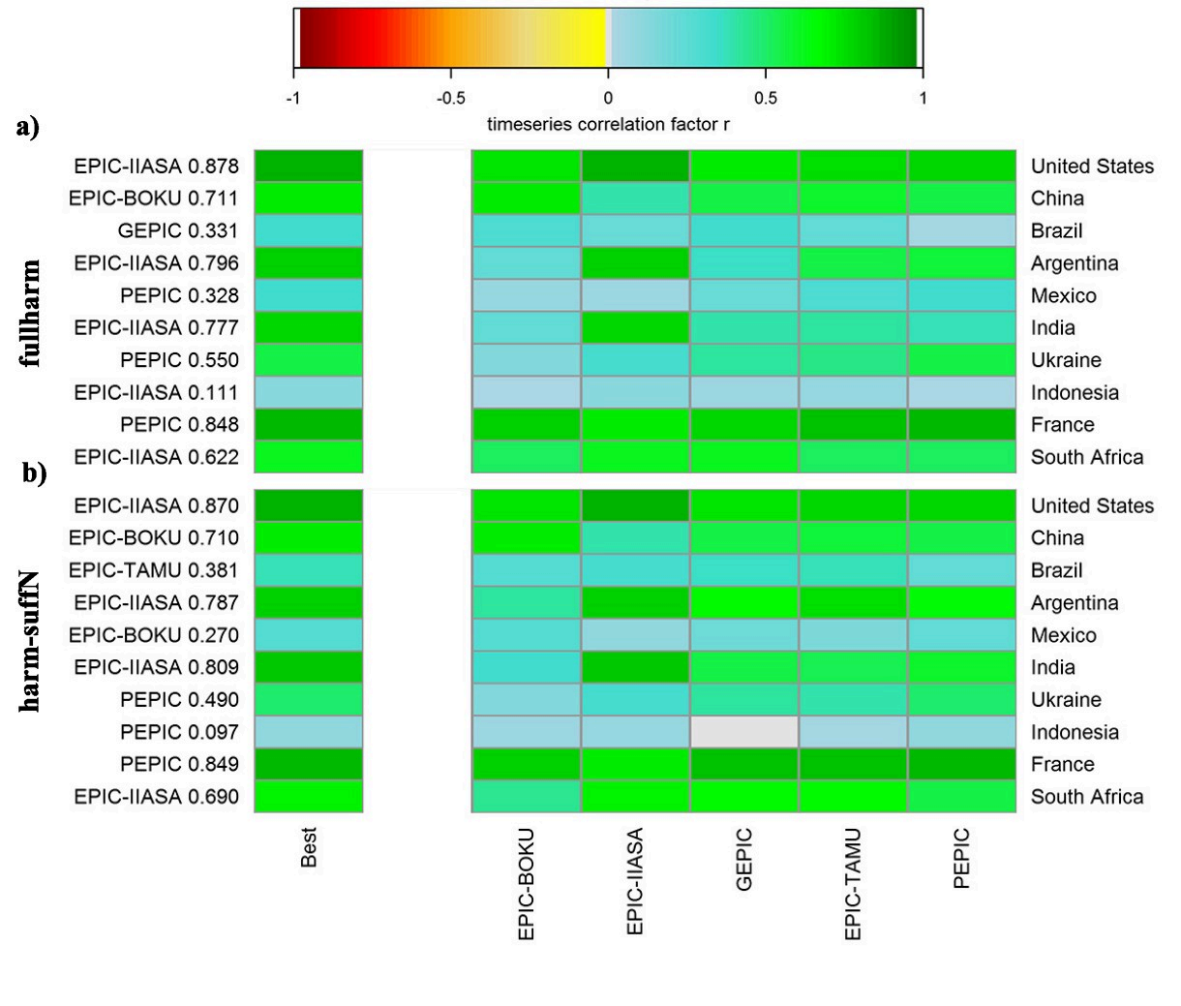
Time-series correlation coefficients against reported detrended yields for EPIC-based GGCMs in the top ten maize producing countries. (a) the fullharm and (b) the harm-suffN simulations. The best performing GGCM including r value is displayed on the left y-axis. Correlation coefficients for each GGCM and country are provided in Table K in S1 File.

For the ten major maize producing countries, substantial variability in GGCM performance can be observed for both the fullharm (Fig 8A) and the harm-suffN (Fig 8B) scenarios. With the fully harmonized setup, EPIC-IIASA shows in most cases the best performance, followed by PEPIC, and finally EPIC-BOKU and GEPIC. All GGCMs show high performance in the USA and France and low performance for Indonesia or Mexico (see also Table K in S1 File). With sufficient nutrient supply, the best performing GGCMs change in various countries, primarily those with overall low to moderate time-series correlation, such as Brazil, Indonesia, and Mexico with decreases in ensemble performance in the latter two (Fig 8B). For some countries in which at least one GGCM has a high performance in the fullharm scenario, several EPIC-GGCMs achieve better results with sufficient nutrient supply, i.e. in Argentina and to a lesser extent in India (Table K in S1 File).

**Fig 9:**
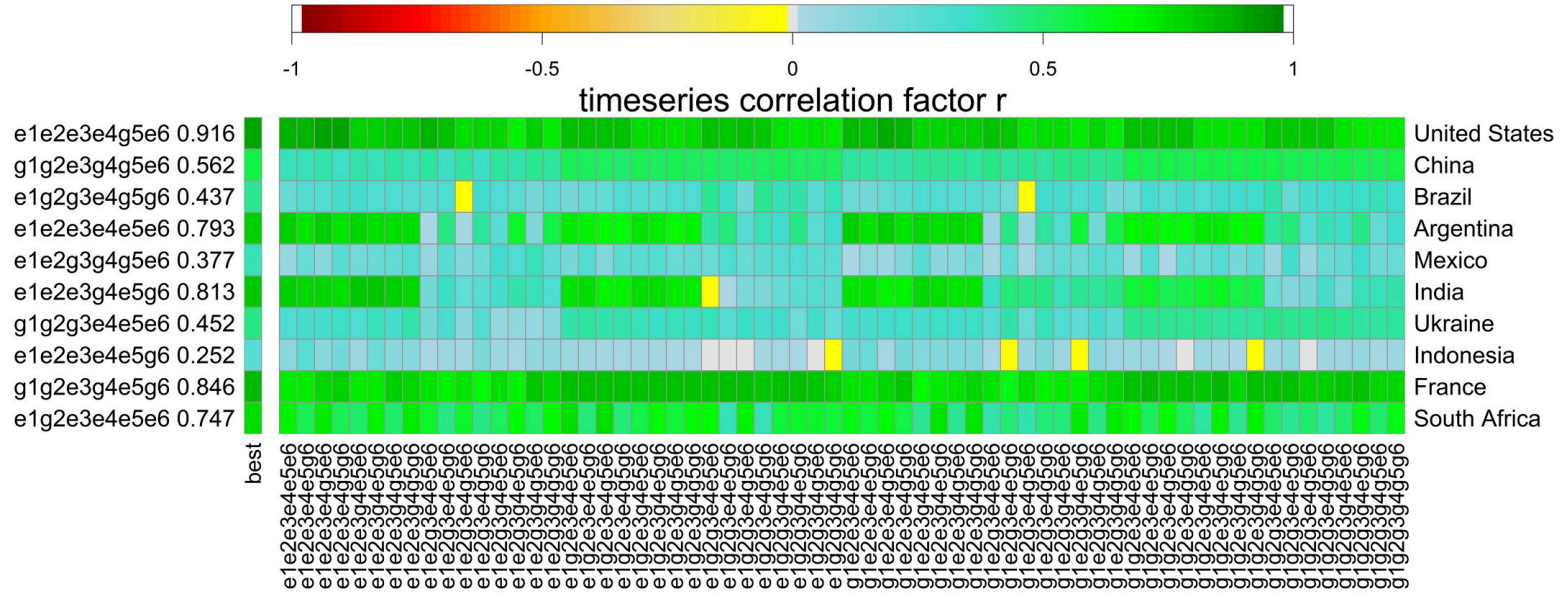
Time-series correlation coefficients against reported detrended yields for all EPIC-IIASA / GEPIC setup combinations. Results are shown for the top ten maize producing countries. GGCM names and r values are shown for the best performing setup in each country. 1 = cultivar distribution, 2 = soil parameters, 3 = soil handling, 4 = nutrient cycling coefficient, 5 = hydrologic coefficients, and 6 = management. e = EPIC-IIASA and g = GEPIC.

Accordingly, also the permutation of setup domains greatly affects the performance (Fig 9). The maximum correlation coefficient increases in all countries compared to the basic fullharm setups of the two GGCMs (Fig 8A), except for Argentina (with slightly lower r due to different digit precisions in output files used in GEPIC). In various countries, the setup with the highest correlation coefficient also exceeds the highest value of the EPIC ensemble (Fig 8A), apart from China and France.

The sensitivity of the GGCM to the selection of setup domains differs considerably among countries with distinct patterns of positive or adverse impacts (Fig U in S1 File). In the USA, the correlation is considerably high with any setup whereas the OM and nutrient cycling parameterization eCoeffN typically provides a higher correlation (Fig U, panel a in S1 File). The opposite is the case for the corresponding GEPIC component gCoeffN. For China in contrast, the performance is overall moderate and most sensitive to soil parameters but also cultivar definitions (Fig U, panel b in S1 File). In Argentina and India (Fig U, panel c,d in S1 File), two countries with low reported fertilizer rates (Table K in S1 File), performance shows a very high sensitivity to setup specifics ranging from r<0 to r>0.8 with lowest results for soil handling from GEPIC (gSoilP). Most setup components however show wide or bimodal distributions indicating that relative performance of single domains depends on the combination with other parameters.

## Discussion

### Effects of harmonization on GGCM agreement

At the pixel level, harmonization expectedly decreases differences between simulated means and increases correlation among the EPIC-based GGCMs in most parts of the world (Figs 3 and 4), driven by harmonized growing seasons and more importantly increasing conformity in nutrient supply. Considering that EPIC-GGCMs agree on average in the majority of grid cells with respect to inter-annual variability (Table I in S1 File) and have low remaining differences in means if nutrient deficits are eliminated (Table G in S1 File) shows that this element causes here the greatest impact in GGCM convergence. This is most apparent under irrigated conditions where the agreement in inter-annual variability improves to a minimum level of significance in 7% of grid cells form default (17%) to fullharm (24%) setups and in >45% from fullharm to harm-suffN (69%). Yet, substantial differences remain in inter-annual variability in about one third and CV_av_ is > 30% in more than a quarter of grid cells highlighting that setup components other than nutrient supply and growing seasons cause lower but still significant impacts on yield estimates. The fact that CV_av_ among GGCMs increases in few regions (e.g. Fig I in S1 File) indicates that data used for harmonization will require further evaluation and continued improvement to best reflect actual on-ground conditions. E.g., the harmonized growing seasons for Southern China reflect a side season in winter, while typically summer maize is planted in the region [87], which may explain the low simulated yields. Vice versa, the fact that GGCMs exhibit highly diverse response to harmonization implies that also GGCMs will need to be iteratively adapted to harmonized input data in future experiments, while it was in this experiment prescribed not to adapt default parameterizations in the harmonized scenarios [34].

### Effects of setup domains on GGCM agreement and performance

#### Cultivar distributions

Cultivar distributions (Fig 1) resulted mostly in the scaling of yields (Fig 7) due to different parameterizations of the upper and lower HI coefficients as PHU are prescribed by growing season input data and climate. The lower HI boundary is approached under water stress and hence renders the EPIC model more susceptible to water deficits during the reproductive growth stage [58], affecting also the magnitude of inter-annual yield variability but hardly inter-annual dynamics as such (Fig 7). Additional effects may still incur from cycling of plant residues, whose amounts depend on the fraction of crop removed at harvest. Planting a cultivar with lower base and optimum temperatures (Fig 1A) contributes to higher yield estimates from EPIC-IIASA in most of temperate Europe and Russia (not shown). Overall, the effect of cultivar distributions on GGCM agreement for inter-annual yield variability (Table J in S1 File) is lower than that of nutrient supply (Table H in S1 File) but can have substantial impacts in temperate and tropic regions.

#### Soil attribute estimation, soil handling, and crop nutrient supply

Among the two EPIC-GGCMs compared in detail, soil parameters differ hardly in primary characteristics, but considerably in the derived hydrologic properties FC, WP, and K_**S**_ (Fig C in S1 File), which can either be input or estimated by EPIC based on various routines (Table 2). These parameters have a substantial effect on inter-annual yield variability (Fig 7E and 7M). They affect modeled drought stress by defining soil water storage capacity and hence the plant available water volume, and also modulate nutrient availability from soil OM mineralization, especially if a dynamic soil profile is assumed. Estimation methods for FC and WP depend on the set of soil samples for which they were developed [88,89]. At present, there is no single optimal method for deriving soil hydraulic parameters globally [90] with none of the methods employed here providing the best performance across the majority of the main producing countries (Fig 9) and substantially varying sensitivities to these parameters among regions (Fig U in S1 File).

Whether soils are treated dynamically with transient carry-over of all soil properties or statically with annual re-initialization of soil properties greatly influences performance (Table 4 and Figs 8 and 9). Especially GEPIC and PEPIC, which have dynamic profile handling and consider soil erosion processes, exhibit high performance in the least number of countries for the fullharm setup, which improves if sufficient nutrients are supplied (Table 4). The GGCMs showing the best performance in terms of total number of countries with good performance in the fullharm scenario and the lowest increase in the harm-suffN scenario are EPIC-BOKU and EPIC-IIASA, both of which employ static soil profiles. In the case of EPIC-IIASA the setup results in a dominance of climatic stresses over nutrient supply (Fig O, panel a in S1 File) compared to GEPIC, which has substantial nutrient limitations in low-input regions (Fig O, panel p in S1 File). The findings are in line with an earlier performance evaluation of the ensemble investigated here [49], which also showed that GGCMs with a static soil profile exhibited often better skills in reproducing national inter-annual yield variability.

Hence, while dynamic soil profiles are essential for assessing agricultural externalities such as soil OM dynamics as well as climate change adaptation and mitigation options [21,22], static soil profiles and even more so sufficient nutrient supply appear key for obtaining high performance in terms of inter-annual yield variability. Obviously, inter-annual dynamics of nutrient supply from OM dominate—especially in the case of limited fertilizer supply—over inter-annual climate dynamics, hampering the signal of the latter in inter-annual yield dynamics. This is perspicuous, as the limited number of pixels in a given country at the employed spatial resolution cannot be expected to reflect OM dynamics on actual farmland. Rather, the latter must be assumed to level out at the country-scale to a certain extent, leaving a signal that is to a larger or smaller extent climate driven [67] and can hence be better picked up by GGCMs not considering soil dynamics.

This poses a dilemma for GGCM performance evaluation and applications to crop-soil-management studies. It may lend itself to assess GGCM performance with respect to climate-driven inter-annual yield variability using a static soil profile and/or sufficient nutrient supply, but to use dynamic soil profiles and business-as-usual fertilization rates for impact and adaptation assessments. However, the latter requires additional evaluations to ensure the correct representation and parameterization of relevant processes, which is as of now strongly limited by lack of globally representative data.

#### Soil organic matter and hydrologic process parameterization

The parameterizations of soil OM and nutrient turnover encompass in various cases recommended parameter ranges [91] and hence a wide range in assumptions on microbial process dynamics. For example, coefficients for slow to passive humus partitioning (Table 2, parameter 23) or N volatilization (parameter 26) cover ranges of one to two orders of magnitude. Values for both parameters are at the edges of recommended ranges [91] and hence bracket extreme cases. Herein, the parameterization of soil OM-related processes was found to be of considerable importance concerning GGCM performance, which depends strongly on this set of parameters in few countries (Fig U in S1 File) but with no clear superiority of either setup from EPIC-IIASA or GEPIC (Fig 9). Yield magnitudes and inter-annual dynamics are greatly affected as well depending on which parameter set is selected (Fig 7). Various field and regional studies have shown that OM partitioning and turnover parameters for CENTURY [60], which is at the core of the OM cycling routines of EPIC and a range of other crop models such as DSSAT or DayCent, are subject to substantial uncertainty as OM pools are conceptually represented and hence not feasible to measure [92]. Therefore, these parameters typically require calibration to regional conditions, which is presently lacking within the ensemble and generally difficult at the global scale due to limited data availability. GGCM setups may here need to be informed by cross calibration based on representative site data [93] beyond crop yields.

We found a rather low sensitivity to hydrologic model domains (Figs 7 and 9; Fig T; Fig U in S1 File). In a recent study, Liu et al. [31] reported that the PET estimation method PM provides the best results globally within the otherwise constant setup of PEPIC with respect to national crop yield estimates, albeit with overall minor differences compared to using the HG method. As for soil OM cycling, methods for hydrologic processes such as algorithms for PET estimation have often been developed for specific regions or require local calibration for optimal performance [94], which is as well presently not implemented in the ensemble members.

#### Crop management operations

The substantial impact of crop nutrient supply as such on agreement and performance of GGCMs has already been discussed above. General management coefficients for irrigation water and fertilizer application (Table 2, parameter 27–29) follow pragmatic assumptions rather than the representation of actual farming systems. Low trigger thresholds (i.e. high values for parameters 27 and 29) allow for a more rapid plant stress reduction but cause in the case of suboptimal fertilizer application rates an earlier consumption of the annual maximum rate, especially if also a low trigger threshold is selected for irrigation water application, which can result in stronger leaching. Low fertilizer application thresholds in contrast do not allow for full plant stress reduction in the harm-suffN scenario. In contrast to the four other EPIC-based GGCMs, PEPIC was set up with a rigid timing of fertilizer application, which likely contributes to the negative yield trend observed. A study based on the same GGCM with automatic fertilizer application of the same annual amounts resulted in overall higher yields [95].

The field operations compared for GEPIC and EPIC-IIASA (Table C in S1 File) differ most substantially in the removal of plant residue and row spacing, affecting long-term nutrient availability and potential biomass estimation, both of which depend in practice on socio-economic decisions and prevailing practices on-farm or locally.

### Representativeness of setups for global agricultural systems

The inherently different assumptions on agro-environmental systems parameterization within the EPIC-based GGCM ensemble result in greatly varying crop growth conditions and limitations. For EPIC-BOKU the average global yields in the default setup indicate the virtual elimination of nutrient stresses (Fig 2A). Similarly, EPIC-IIASA exhibits in its fullharm setup dominant climate stresses (Fig O, panel a; Fig R, panel a in S1 File), which can be assumed to be highly similar in the default setup that is based on nearly identical input data (Text C in S1 File) and shows only slightly lower global average yields (Fig 2A). For GEPIC in contrast, productivity on large parts of the global cropland–especially in the tropics—is limited by nutrient deficits (Fig R, panel b in S1 File), which can also be expected for PEPIC based on the identical cultivar distribution, similar global average yield levels (Fig 2A), and the consideration of dynamic soil handling (Table 2). EPIC-TAMU presents a compromise including soil OM dynamics–with rather rapid turnover rates and moderate N volatilization (Table 2)—over time but no water erosion. The substantial differences in nutrient deficits and associated climate sensitivity can hence also explain why in two earlier studies EPIC-BOKU responded more sensitively to climatic change than GEPIC [6], and EPIC-BPOKU and EPIC-IIASA provided more explained variance for weather-induced yield variability than GEPIC [96]. Thus, the large variation in yield projections from various GGCMs seen in e.g. Rosenzweig et al. [6] for a smaller ensemble may be as much due to setup as to model structural differences.

However, due to lack of spatial parameterizations except for a limited number of cultivars, none of the GGCMs can be expected to represent a globally optimal setup. The improved GGCM performance if setup domains are permutated (Fig U in S1 File and Fig 9) and the presence of all EPIC-based GGCMs among the top performing GGCMs (Fig 8 and Table 4) highlight that no globally uniform parameterization performs optimally for all countries. Instead, the EPIC ensemble allows for covering a range of uncertainties that may exist in a given pixel or the sub-grid scale [97] below the spatial resolution of 0.5° x 0.5°, e.g. through the representation of low-input or high-input agricultural systems and associated plant growth limitations, which coexist in close proximity especially in transition countries [98]. The diversity in cultivars, soil types, and tillage regimes potentially occurring at the sub-grid level, however, cannot be assumed to be fully covered by the range of setups present in the ensemble. In addition, all GGCMs assume high-input agriculture in regions with high reported average fertilizer application rates (Fig E in S1 File), which neglects potential imbalances and heterogeneity in input systems present in these regions. For a more targeted representation of agricultural systems within the ensemble, setups may rather need to be defined in a systematic way to cover distinct agricultural production systems for impact studies.

Increasing the spatial resolution of input data may suggest a viable alternative to bracketing uncertainties at the sub-grid scale. Although this has been shown to improve performance or at least affect yield estimates, especially in environmentally heterogeneous regions with respect to soils, climate, and topography (e.g. [29,43,99]), it can at present not be expected to improve the representation of management practices. Global data on cultivar distributions even between broad regions are lacking, which requires assumptions by modelers (cf. Fig 1). The same is the case for tillage practices including residue management, which may also vary in time subject to farmers’ management preferences, economic opportunities, and incentives for specific agricultural practices. Albeit spatial data on representative management practices such as tillage systems are in the process of being compiled globally [100] and remote sensing products may allow for spatial attribution of field management practices in the future [101,102], employing an ensemble to bracket uncertainties appears the most robust approach meanwhile. Nevertheless, increasing the spatial resolution will be required to better reflect actually cultivated soils [27] or microclimate [99] at the sub-grid scale. However, the fact that high performance could be achieved herein for the majority of countries (Figs 8 and 9; Fig AB in S1 File and Table 4) indicates that the presently employed resolution is in most regions sufficient for obtaining robust nationally weighted estimates.

### The EPIC-based GGCMs in the context of a wider ensemble

Performing key analyses also for a wider ensemble of six GGCMs based on other core models (Text D; Figs U to AE and associated tables in S1 File) shows in various aspects contrasting results. While global average yield estimates converge among the EPIC-based GGCMs, they diverge in the wider ensemble (Fig V in S1 File). Importantly, the spread among EPIC-based GGCMs and the non-EPIC-based in the default setup is highly comparable indicating that the differences in input data, parameterization, and management assumptions are for absolute yield levels of similar importance as core model selection itself. The wide range in estimates for non-nutrient limited yield potentials (Fig V, panel f in S1 File) can be attributed to very high or low cultivar productivity in specific GGCMs due to core model processes as such and cultivar parameterizations. The increase in absolute yields from the fullharm to harm-suffN scenario, which is substantial for some GGCMs as indicated by the threefold increase for PEGASUS, shows that also most of the non-EPIC-based GGCMs are moderately to highly responsive to nutrient supply, but that their sensitivities vary greatly.

Similar to absolute yield estimates, the range of correlation coefficients of global mean inter-annual yield variability compared to FAOSTAT reported data increases with harmonization for the non-EPIC-based sub-ensemble (Fig AD in S1 File) due to decreasing skill for some GGCMs. The EPIC-based sub-ensemble has here a comparably constant range from default to fullharm and a narrower in the harm-suffN scenario. Both metrics indicate that harmonization tends to improve the agreement among GGCMs with the same structure but not among structurally different GGCMs, which vary in their sensitivities to specific setup components such as nutrient inputs and have partly been calibrated in their default setups to reflect reported crop yields [49]. Except for the default scenario, these results are in line with a recent field-scale study finding that crop model structure has a greater impact on deviations than parameterization [103]. Yet, the default scenario is not comparable here, as ground conditions such as management and soil are typically well known at the field scale but pose substantial uncertainty in global studies.

A common finding across the whole ensemble is that GGCM performance for major producing countries is highest for GGCMs with either a static soil profile or sufficient nutrient supply and in most cases performance of individual GGCMs is higher with sufficient nutrient supply compared to the fullharm setup (Fig AB in S1 File). E.g., PEGASUS only considers hydrology in its soil module but has a multiplicative plant stress impact combining temperature, water, and nutrient limitations from insufficient fertilizer application [104] resulting in higher performance in most countries in the harm-suffN setup (Fig AB in S1 File). As pointed out above, this suggests that GGCMs should not only be evaluated for business-as-usual fertilizer application but also sufficient nutrient supply if inter-annual yield variability is the benchmark. The increasing spreads in mean yields and correlations with reported yields (Fig V and Fig AD in S1 File), partly caused by very low yields or negative trends in yields, furthermore suggest that while most GGCMs have been positively evaluated in their default setups and partly been calibrated to reproduce reported yields, the resultant default parameterizations may not be compatible with the harmonization approach taken here. This suggests that harmonization should be an iterative process with re-evaluation of GGCMs.

The agreement in inter-annual yield variability among GGCMs at the pixel level is far lower for the whole ensemble (Fig W in S1 File) or the non-EPIC based GGCMs (Fig Y in S1 File) than for the EPIC-based GGCMs alone and only shows a trend in improvement with increasing harmonization (Table M in S1 File), which is not evident if the EPIC-based GGCMs are not considered (Table O in S1 File). The substantial divergence even in the harm-suffN scenario, where under non-nutrient limited conditions plant phenology and photosynthesis dominate biomass accumulation and yield formation, indicates that these processes—differing greatly among GGCMs and core models (Table A in S1 File)—exhibit differences that exceed the impact of nutrient supply in the EPIC-based sub-ensemble (Table I in S1 File). Spatially, the agreement is best in temperate and cold regions of the northern hemisphere with clear climatic constraints of growing seasons, which impacts planting and harvest in the default setups but may also affect cultivar definitions carried over to the harmonized setups. Similar to the EPIC-based GGCMs, agreement is poor across the tropics. Interestingly, the non-EPIC-based GGCMs also show little agreement in arid regions, indicating substantial differences in crop water use and/or soil hydrology, which can result either from process implementations in the core models or from divergence in soil input data.

Albeit GGCMs have partly been harmonized in this study, it is at this point not feasible to attribute differences in GGCM outputs to specific setup domains ranging from input data to parameterization and process representation. A basic evaluation (Fig AC in S1 File) indicates that GGCMs with site-based core models and with prescribed leaf area development have a tendency towards higher skill in reproducing average global inter-annual yield variability. However, due to the sample bias caused by the multiple EPIC-based GGCMs, robustly identifying model routines and structures providing high skill will require more scrutiny. As pointed out below, this will also require further harmonization or in-depth analyses for contrasting locations.

Finally, evaluations of the multi-GGCM mean (MGM) for simulated vs reported global inter-annual yield variability show that the MGM has typically a higher score than individual GGCMs across (sub-)ensembles and scenarios (Fig AD in S1 File), analogously to earlier findings for an ensemble of structurally different field-scale wheat models [19]. An exception occurs for the harmonized setups of the EPIC-based sub-ensemble in which some GGCMs have higher skill. The highest time-series correlation coefficient occurs for the MGM of the whole ensemble in the default and harm-suffN setups, but is marginally surpassed by the MGM of the non-EPIC-based GGCMs in the fullharm scenario. These evaluations indicate that the MGM of the whole ensemble has a higher gain from including GGCMs with structural differences rather than multiple configurations of the same core model. The resilience of MGM for the whole ensemble to exclusion of specific GGCMs (Fig AE in S1 File) furthermore shows that the incompatibility of single GGCMs with harmonization does hardly affect the ensemble mean.

### Conclusions for global crop model and ensemble applications

#### Extension of sensitivity analyses and model calibration

Our findings highlight the importance of parameter choices in global-scale crop modeling studies that have not received much attention so far. While recent years have seen a vast growth in sensitivity analyses and calibration efforts of crop models at the field, local, and regional scales [103,105–107] with increasing methodologic sophistication [108], GGCMs have been subject to such studies only to a very limited extent (e.g. [109,110]). In all cases, a focus is typically on directly plant growth related parameters (e.g. photosynthesis, leaf development, or temperature response) or these are identified as the most sensitive variables.

Acknowledging the narrow parameterization of cultivars herein, our findings show that other setup components such as soil processes and parameters have substantial impact on yield estimates (Fig 7; Fig T; Fig U in S1 File). Such parameter domains should hence be included in future sensitivity analyses in order to derive a full picture of model sensitivity globally. The same applies to calibration efforts as uncertainties related to agro-environmental processes and management are otherwise projected unto plant or cultivar coefficients and will likely affect impact assessments or evaluations of adaptation measures. At the regional scale, the sensitivity of EPIC to soil and management parameters alone has been evaluated in a very recent impact study addressing climate change and soil degradation in Europe [33], indicating high sensitivity to soil and management parameters, which partly outweigh climate impacts.

#### Bracketing uncertainties and potential for further harmonization

The GGCM parameterizations by individual modeling groups do not reflect poor vs. careful parameterization but the lack of reference and input data on many aspects of agricultural production systems, such as soil and crop management. The uncertainty in management and soil parametrization is a problem that is specific to large-scale and global gridded crop model applications, while field-scale assessments have typically precise information on management practices, variety characteristics and soil properties. Still, the importance of these parameters for simulation results, as shown here, demonstrates that simple extrapolation of a small set of site-specific parameterizations is not possible. Global-scale applications are thus challenged to improve on the representation of management and soil conditions. In addition, applications of crop models at all scales need to better understand and represent the dynamics in environmental (e.g. soil degradation) and management (e.g. variety selection) conditions. Thus, employing a large GGCM ensemble with substantial differences in setups presents an asset as it allows for covering some of the uncertainties in the range of possible environment x management conditions, but the management aspects need to be better represented in models by process implementation and better informed by suitable input data.

Whether to further harmonize GGCM setups for ensemble runs is primarily a question of whether (a) GGCMs are being evaluated with respect to procedural differences (comparison studies) or (b) ranges of agro-environmental conditions are to be covered (impact assessments). New datasets relevant for GGCMs are continuously becoming available and may aide in reducing or attributing uncertainties within the ensemble for case (a).

Recently, Gbegbelegbe et al. [111] have presented a global distribution of representative wheat cultivars for crop modelling based on extensive data on physiology and global agro-climatic zoning. The same is in principle feasible for maize using agro-climatic mega-environments [112]. Cultivar specifications, however, are difficult to translate between models and will therefore require new data management tools as proposed by Porter et al. [113]. Global tillage practices–albeit not relevant for all GGCMs (Table A in S1 File)—are presently being compiled [100] and will likely become publically available in the near future.

Soil properties lend themselves to be harmonized to avoid differences in nutrient supply in low-input regions from SOM mineralization and especially differences in soil hydrology once appropriate data become available. The derived soil parameters FC, WP, and K_S_, which were here shown to play a major role in drought response and soil microbial processes, are used directly or have a counterpart in all GGCMs. For optimal results, they need to be estimated based on regionally calibrated functions and algorithms [90]. Albeit a new global soil database provides these estimates, they were derived based on soil samples foremost from sub-Saharan Africa and can hence not be considered representative globally (update of [114]), while a dataset specifically calibrated for Europe exists [115]. Spatially parameterizing microbial turnover of soil OM and nutrients in turn appears not feasible in the near term due to lack of comprehensive field data.

#### Implications for the wider ensemble and ensemble studies

Several of the conclusions stated above also apply to the wider ensemble of this study, such as the potential for further harmonization of input data and setups on the one hand, but also the requirement for iterative GGCM parameterization and re-evaluation after harmonization on the other. I.e., local and global increases in deviations with harmonization underpin that this is not a trivial process. Our results suggest that common input data, foremost growing seasons, will require more scrutiny to ensure they reflect the most common practices. Within this study, the protocol mandated that default parameterizations remain constant among scenarios to study the effect of harmonization without confounding impacts of adjusted parameters [34]. Yet, unanticipated GGCM behavior, e.g. resulting in lower than expected yields or negative yield trends despite sufficient nutrient supply (Fig F, Fig W, Table L in S1 File), suggests that in future studies employing harmonized ensembles, GGCMs should be re-evaluated after harmonization to ensure valid responses to changes in input data and crop management. Noteworthy, our findings are robust against excluding individual GGCMs (Text D in S1 File).

Further findings for the EPIC-based sub-ensemble will become relevant for other GGCMs with the progressing inclusion of crop-soil-management-related processes. The majority of non-EPIC-based GGCMs shows a moderate to strong response to nutrient supply from the fullharm to harm-suffN scenario, which can be substantial as in the case of PEGASUS. Accordingly, the progressing inclusion of agro-environmental processes such as transient nutrient cycling [39–42], presently only accounted for in the EPIC-based GGCMs within this ensemble, bears the potential to further exacerbate differences in GGCM outputs.

Evaluations of the EPIC-based sub-ensemble show that the inclusion of multiple configurations of the same core model has several merits in bracketing uncertainties. However, the MGM performance for different sub-ensembles (Text D in S1 File; Fig AE in S1 File) highlights that the ensemble mean has a higher gain from including structurally distinct GGCMs compared to varying configurations of the same core model. Yet, the range of GGCM skills and their divergence with harmonization suggest that GGCM setups are of high importance as well and performance of both single GGCMs and ensemble means are typically highest in the default setups for which most GGCMs have originally been set up and evaluated.

Investigating the role of specific model processes or process conceptualizations in detail is beyond the scope of this study and the underlying experiment. Grouping GGCMs by basic characteristics suggests higher skill in reproducing global average inter-annual yield dynamics for site-based GGCMs and those with prescribed phenology. However, this will need to be studied in more detail across scales in specifically designed experiments covering both parameterizations and process representations based on different conceptualizations.

## Supporting information

S1 File The supplementary information (SI) is provided in one single PDF file. **Text A.** Relevant routines of the EPIC model. **Text B.** Differences between EPIC model versions v0810 and v1102. **Text C.** Description of EPIC-based GGCMs. **Text D.** Evaluations of the wider ensemble. **Table A.** Relevant characteristics of GGCMs in this study based on Müller et al. [30]. **Table B.** Legend for Table 2 in main paper with brief explanation for parameters differing among EPIC-based GGCMs. **Table C.** Crop management operations of EPIC-IASA and GEPIC. **Table D.** Parameterization of different maize cultivars used in the GGCMs as shown in Fig 1 of the manuscript with corresponding coloring of column headings. **Table E.** Relative spread of maize yield estimates measured as yields of the highest estimate in relation to yields of the lowest estimate in Fig 2 of the main paper. **Table F.** Statistical coefficients for linear regressions of yield estimates over time in Fig 2 of the main article and in Fig F and mean error [t ha^-1^] compared to reported yields. **Table G.** Quantiles of coefficient of variation [%] among EPIC-GGCMs for grid-wise maize yield estimates (Fig 3 in main article) depending on the setup and management scenarios (see Table 1 in main article). **Table H.** Same as Table G but excluding EPIC-TAMU. **Table I.** Fractions of grid cells [%] in which the median time series correlation among the EPIC-based GGCMs (Fig 4 in main article) fulfils a certain level of significance. **Table J.** Same as Table I but excluding EPIC-TAMU. **Table K.** Time-series correlation coefficient r for each GGCM in the ten major maize producing countries for the fullharm and harm-suffN scenarios (Table 1 in main paper) and annual N fertilizer application rates for maize in each country. **Table L.** Statistical coefficients for linear regressions of yield estimates (not shown) corresponding to global average yields in Fig V and mean error [t ha^-1^] compared to reported yields. **Table M.** Fractions of grid cells [%] in which the median time series correlation among all GGCMs (Fig W) fulfils a certain level of significance. **Table N.** Same as Table M but excluding EPIC-TAMU, LPJmL, and LPJ-GUESS. **Table O.** Fractions of grid cells [%] in which the median time series correlation among the GGCMs, excluding the EPIC-based ones, (Fig Y) fulfils a certain level of significance. **Table P.** Same as Table O but excluding LPJmL and LPJ-GUESS. **Fig A.** Maize yield estimates of EPIC v0810 and EPIC v1102 for four contrasting locations. **Fig B.** Schematic representation of decadal GEPIC runs with dynamic soil profile and erosion for (a) high nutrient input and (b) low nutrient input conditions. **Fig C.** Density distributions of key soil parameters in the original ISRIC WISE dataset used in GEPIC and the processed soil parameters used in EPIC-IIASA based on WISE. **Fig D.** Major Koeppen-Geiger climate regions according to Peel et al. [33] based on the climate data used in this study. **Fig E.** (a) Nitrogen and (b) phosphorus fertilizer application rates used in the fullharm setup [34]. **Fig F.** Same as Fig 2 in the main manuscript but with linear regressions included but without ensemble mean and reported yields. **Fig G.** Same as Fig 3 in the main body but excluding EPIC-TAMU. **Fig H.** Same as Fig 4 in the main body but excluding EPIC-TAMU. **Fig I.** Long-term maize yield estimates for the EPIC-based GGCMs in two spatial units at administrative level 2 with increase in CV_av_ after harmonization in Fig 3 of the main body. **Fig J.** Median time-series correlation coefficient r for maize yields among EPIC-based GGCMs compared to binned fertilizer application rates in the fully harmonized management scenario (fullharm) with sufficiently irrigated (a-d) or rainfed (e-h) water supply in each grid cell of four major climate regions. **Fig K.** Coefficient of variation (CV_av_) among maize yield estimates in the harm-suffN scenario in grid cells in which either all GGCMs plant the high-yielding cultivars 1 or 2 (Fig 1 in main paper) or in which at least four GGCMs plant the low-yielding drought-sensitive cultivar 4 or in which cultivar types are mostly mixed. **Fig L.** Median time-series correlation coefficient in the harm-suffN scenario in grid cells in which either all GGCMs plant the high-yielding cultivars 1 or 2 (Fig 1 in main paper and Table D) or in which at least four GGCMs plant the low-yielding drought-sensitive cultivar 4 or in which cultivar types are mostly mixed. **Fig M.** Frequency distribution of time-series correlation coefficients among EPIC-based GGCMs in each grid cell and for each management scenario. **Fig N.** Global average rainfed maize yields over a 29 year period for 64 setup combinations based on the EPIC-IIASA and GEPIC setups (Table 3 in main article). **Fig O.** Box-and-whisker plots of global averaged growth stresses over a 29 year period for 64 setup combinations based on the EPIC-IIASA and GEPIC setups under rainfed conditions (Table 3 in main paper). **Fig P.** (a) Monthly total biomass and (b-c) stress occurrence for a single year in a randomly sampled grid cell of the US Corn Belt differing the managements of EPIC-IIASA and GEPIC (eManage/gManage) with otherwise identical setups (included in Fig 7A of the main article) and a static soil profile (eSoilP). **Fig Q.** Annual (a-c) yields, (d-f) stresses, (g-i) water fluxes, and (j-l) nitrogen fluxes for a randomly sampled grid in Ukraine with low fertilizer application for three EPIC-GGCM setups differing in soil parameters (SoilD) and OM/nutrient cycling parameterization (CoeffN) using dynamic soil profile handling (gSoilP). **Fig R.** Dominant stress per grid cell averaged over the simulation period in four selected setups shown underneath each panel. **Fig S.** Correlation matrix for the 64 setup permutation of EPIC-IIASA and GEPIC. Colour indicates the correlation coefficient r as shown on the right scale, circle sizes represent the level of significance. **Fig T.** Distributions (violins) and box-and-whisker plots of correlation coefficients among all setup combinations of EPIC-IIASA and GEPIC (Table 3 in main article) aggregated by setup domains. **Fig U.** Distributions (violins) and medians (horizontal lines) of the time series correlation coefficient r of simulated and reported yields for each setup domain (Table 3 in main article) in (a) USA, (b) China, (c) Argentina, and (d) India. **Fig V.** Global average area-weighted maize yields and 95% confidence interval of the mean for EPIC-GGCMs and non-EPIC-based GGCMs for three management scenarios. **Fig W.** Median of time-series correlation coefficient r for maize yield estimates among the whole GGCM ensemble for each of the six crop management scenarios defined in Table 1 of the main article. **Fig X.** Same as Fig W but excluding EPIC-TAMU, LPJmL, and LPJ-GUESS. **Fig Y.** Median of time-series correlation coefficient r for maize yield estimates among the GGCM ensemble excluding the EPIC-based GGCMs for each of the six crop management scenarios defined in Table 1 of the main article. **Fig Z.** Same as Fig Y but excluding LPJmL and LPJ-GUESS. **Fig AA.** Frequency distributions of time-series correlation coefficients in each grid cell for all GGCMs and setup scenarios (Table 1 in main article). Solid and dashed lines at the top of each panel indicated the location of the major peak in the distribution for rainfed (dashed) or sufficiently irrigated (solid) simulations of each management scenario (Table 1 in main article). **Fig AB.** Time-series correlation coefficients for all GGCMs with the fullharm and harm-suffN scenarios (x-axis) in the top ten maize producing countries (right y-axis) and the best performing GGCM/setup combination including the r value (left y-axis). **Fig AC.** Time-series correlation coefficients for GGCMs grouped by basic characteristics (Table A) for the three setup scenarios. **Fig AD.** Box-and-whisker plots of time-series correlation coefficients for single GGCMs against FOASTAT global reported yields grouped into the (sub-)ensembles “All GGCMs”, “EPIC-based GGCMs”, and “non-EPIC-based GGCMs” for each setup scenario (a-c). **Fig AE.** Box-and-whisker plots of time-series correlation coefficients for permutations of multi-GGCM means excluding one GGCM at a time against FOASTAT global reported yields for each setup scenario (a-c).
(PDF)

## References

[1] Tan G, Shibasaki R. Global estimation of crop productivity and the impacts of global warming by GIS and EPIC integration. Ecological Modelling. 2003;168: 357–370. 10.1016/S0304-3800(03)00146-7

[2] Liu J, Folberth C, Yang H, Röckström J, Abbaspour K, Zehnder AJB. A Global and Spatially Explicit Assessment of Climate Change Impacts on Crop Production and Consumptive Water Use. PLOS ONE. 2013;8: e57750 10.1371/journal.pone.0057750 23460901 PMC3583897

[3] Balkovič J, van der Velde M, Skalský R, Xiong W, Folberth C, Khabarov N, et al Global wheat production potentials and management flexibility under the representative concentration pathways. Global and Planetary Change. 2014;122: 107–121. 10.1016/j.gloplacha.2014.08.010

[4] Elliott J, Deryng D, Müller C, Frieler K, Konzmann M, Gerten D, et al Constraints and potentials of future irrigation water availability on agricultural production under climate change. PNAS. 2014;111: 3239–3244. 10.1073/pnas.1222474110 24344283 PMC3948288

[5] Folberth C, Yang H, Gaiser T, Liu J, Wang X, Williams J, et al Effects of ecological and conventional agricultural intensification practices on maize yields in sub-Saharan Africa under potential climate change. Environ Res Lett. 2014;9: 044004 10.1088/1748-9326/9/4/044004

[6] Rosenzweig C, Elliott J, Deryng D, Ruane AC, Müller C, Arneth A, et al Assessing agricultural risks of climate change in the 21st century in a global gridded crop model intercomparison. PNAS. 2014;111: 3268–3273. 10.1073/pnas.1222463110 24344314 PMC3948251

[7] Müller C, Elliott J, Chryssanthacopoulos J, Deryng D, Folberth C, Pugh TAM, et al Implications of climate mitigation for future agricultural production. Environ Res Lett. 2015;10: 125004 10.1088/1748-9326/10/12/125004

[8] Deryng D, Elliott J, Folberth C, Müller C, Pugh TAM, Boote KJ, et al Regional disparities in the beneficial effects of rising CO_2_ concentrations on crop water productivity. Nature Climate Change. 2016;6: 786–790. 10.1038/nclimate2995

[9] Bondeau A, Smith PC, Zaehle S, Schaphoff S, Lucht W, Cramer W, et al Modelling the role of agriculture for the 20th century global terrestrial carbon balance. Global Change Biology. 2007;13: 679–706. 10.1111/j.1365-2486.2006.01305.x

[10] Liu J, Williams JR, Zehnder AJB, Yang H. GEPIC–modelling wheat yield and crop water productivity with high resolution on a global scale. Agricultural Systems. 2007;94: 478–493. 10.1016/j.agsy.2006.11.019

[11] Fader M, Rost S, Müller C, Bondeau A, Gerten D. Virtual water content of temperate cereals and maize: Present and potential future patterns. Journal of Hydrology. 2010;384: 218–231. 10.1016/j.jhydrol.2009.12.011

[12] Stehfest E, Heistermann M, Priess JA, Ojima DS, Alcamo J. Simulation of global crop production with the ecosystem model DayCent. Ecological Modelling. 2007;209: 203–219. 10.1016/j.ecolmodel.2007.06.028

[13] Liu W, Yang H, Ciais P, Stamm C, Zhao X, Williams JR, et al Integrative Crop-Soil-Management Modeling to Assess Global Phosphorus Losses from Major Crop Cultivations. Global Biogeochemical Cycles. 2018;32: 1074–1086. 10.1029/2017GB005849

[14] Havlík P, Schneider UA, Schmid E, Böttcher H, Fritz S, Skalský R, et al Global land-use implications of first and second generation biofuel targets. Energy Policy. 2011;39: 5690–5702. 10.1016/j.enpol.2010.03.030

[15] Schneider UA, Havlík P, Schmid E, Valin H, Mosnier A, Obersteiner M, et al Impacts of population growth, economic development, and technical change on global food production and consumption. Agricultural Systems. 2011;104: 204–215. 10.1016/j.agsy.2010.11.003

[16] Müller C, Robertson RD. Projecting future crop productivity for global economic modeling. Agricultural Economics. 2014;45: 37–50. 10.1111/agec.12088

[17] Nelson GC, Valin H, Sands RD, Havlík P, Ahammad H, Deryng D, et al Climate change effects on agriculture: Economic responses to biophysical shocks. PNAS. 2014;111: 3274–3279. 10.1073/pnas.1222465110 24344285 PMC3948295

[18] Asseng S, Ewert F, Rosenzweig C, Jones JW, Hatfield JL, Ruane AC, et al Uncertainty in simulating wheat yields under climate change. Nature Climate Change. 2013;3: 827–832. 10.1038/nclimate1916

[19] Martre P, Wallach D, Asseng S, Ewert F, Jones JW, Rötter RP, et al Multimodel ensembles of wheat growth: many models are better than one. Global Change Biology. 2015;21: 911–925. 10.1111/gcb.12768 25330243

[20] Sándor R, Ehrhardt F, Basso B, Bellocchi G, Bhatia A, Brilli L, et al C and N models Intercomparison–benchmark and ensemble model estimates for grassland production. Advances in Animal Biosciences. 2016;7: 245–247. 10.1017/S2040470016000297

[21] Basso B, Hyndman DW, Kendall AD, Grace PR, Robertson GP. Can Impacts of Climate Change and Agricultural Adaptation Strategies Be Accurately Quantified if Crop Models Are Annually Re-Initialized? PLOS ONE. 2015;10: e0127333 10.1371/journal.pone.0127333 26043188 PMC4456366

[22] Basso B, Dumont B, Maestrini B, Shcherbak I, Robertson GP, Porter JR, et al Soil Organic Carbon and Nitrogen Feedbacks on Crop Yields under Climate Change. ael. 2018;3: 0. 10.2134/ael2018.05.0026

[23] Bassu S, Brisson N, Durand J-L, Boote K, Lizaso J, Jones JW, et al How do various maize crop models vary in their responses to climate change factors? Global Change Biology. 2014;20: 2301–2320. 10.1111/gcb.12520 24395589

[24] Rosenzweig C, Jones JW, Hatfield JL, Ruane AC, Boote KJ, Thorburn P, et al The Agricultural Model Intercomparison and Improvement Project (AgMIP): Protocols and pilot studies. Agricultural and Forest Meteorology. 2013;170: 166–182. 10.1016/j.agrformet.2012.09.011

[25] Osborne T, Rose G, Wheeler T. Variation in the global-scale impacts of climate change on crop productivity due to climate model uncertainty and adaptation. Agricultural and Forest Meteorology. 2013;170: 183–194. 10.1016/j.agrformet.2012.07.006

[26] Zhang X, Izaurralde RC, Manowitz DH, Sahajpal R, West TO, Thomson AM, et al Regional scale cropland carbon budgets: Evaluating a geospatial agricultural modeling system using inventory data. Environmental Modelling & Software. 2015;63: 199–216. 10.1016/j.envsoft.2014.10.005

[27] Folberth C, Skalský R, Moltchanova E, Balkovič J, Azevedo LB, Obersteiner M, et al Uncertainty in soil data can outweigh climate impact signals in global crop yield simulations. Nature Communications. 2016;7: 11872 10.1038/ncomms11872 27323866 PMC4919520

[28] Waha K, Huth N, Carberry P, Wang E. How model and input uncertainty impact maize yield simulations in West Africa. Environmental Research Letters. 2015;10: 024017 10.1088/1748-9326/10/2/024017

[29] Folberth C, Yang H, Wang X, Abbaspour KC. Impact of input data resolution and extent of harvested areas on crop yield estimates in large-scale agricultural modeling for maize in the USA. Ecological Modelling. 2012;235–236: 8–18. 10.1016/j.ecolmodel.2012.03.035

[30] Angulo C, Gaiser T, Rötter RP, Børgesen CD, Hlavinka P, Trnka M, et al ‘Fingerprints’ of four crop models as affected by soil input data aggregation. European Journal of Agronomy. 2014;61: 35–48. 10.1016/j.eja.2014.07.005

[31] Liu W, Yang H, Folberth C, Wang X, Luo Q, Schulin R. Global investigation of impacts of PET methods on simulating crop-water relations for maize. Agricultural and Forest Meteorology. 2016;221: 164–175. 10.1016/j.agrformet.2016.02.017

[32] Wang E, Martre P, Zhao Z, Ewert F, Maiorano A, Rötter RP, et al The uncertainty of crop yield projections is reduced by improved temperature response functions. Nature Plants. 2017;3: 17102 10.1038/nplants.2017.102 28714956

[33] Balkovič J, Skalský R, Folberth C, Khabarov N, Schmid E, Madaras M, et al Impacts and Uncertainties of +2°C of Climate Change and Soil Degradation on European Crop Calorie Supply. Earth’s Future. 2018;6: 373–395. 10.1002/2017EF000629 29938209 PMC5993244

[34] Elliott J, Müller C, Deryng D, Chryssanthacopoulos J, Boote KJ, Büchner M, et al The Global Gridded Crop Model Intercomparison: data and modeling protocols for Phase 1 (v1.0). Geosci Model Dev. 2015;8: 261–277. 10.5194/gmd-8-261-2015

[35] Elliott J, Kelly D, Chryssanthacopoulos J, Glotter M, Jhunjhnuwala K, Best N, et al The parallel system for integrating impact models and sectors (pSIMS). Environmental Modelling & Software. 2014;62: 509–516. 10.1016/j.envsoft.2014.04.008

[36] Holzworth DP, Huth NI, deVoil PG, Zurcher EJ, Herrmann NI, McLean G, et al APSIM–Evolution towards a new generation of agricultural systems simulation. Environmental Modelling & Software. 2014;62: 327–350. 10.1016/j.envsoft.2014.07.009

[37] Jones J, Hoogenboom G, Porter CH, Boote KJ, Batchelor WD, Hunt LA, et al The DSSAT cropping system model. European Journal of Agronomy. 2003;18: 235–265. 10.1016/S1161-0301(02)00107-7

[38] Maharjan GR, Prescher A-K, Nendel C, Ewert F, Mboh CM, Gaiser T, et al Approaches to model the impact of tillage implements on soil physical and nutrient properties in different agro-ecosystem models. Soil and Tillage Research. 2018;180: 210–221. 10.1016/j.still.2018.03.009

[39] Lutz F, Herzfeld T, Heinke J, Rolinski S, Schaphoff S, Bloh W von, et al Simulating the effect of tillage practices with the global ecosystem model LPJmL (version 5.0-tillage). Geoscientific Model Development. 2019;12: 2419–2440. 10.5194/gmd-12-2419-2019

[40] Bloh W von, Schaphoff S, Müller C, Rolinski S, Waha K, Zaehle S. Implementing the nitrogen cycle into the dynamic global vegetation, hydrology, and crop growth model LPJmL (version 5.0). Geoscientific Model Development. 2018;11: 2789–2812. 10.5194/gmd-11-2789-2018

[41] Olin S, Schurgers G, Lindeskog M, Wårlind D, Smith B, Bodin P, et al Modelling the response of yields and tissue C: N to changes in atmospheric CO_2_ and N management in the main wheat regions of western Europe. Biogeosciences. 2015;12: 2489–2515. 10.5194/bg-12-2489-2015

[42] Goll DS, Vuichard N, Maignan F, Jornet-Puig A, Sardans J, Violette A, et al A representation of the phosphorus cycle for ORCHIDEE (revision 4520). Geosci Model Dev. 2017; 26.

[43] Hoffmann H, Zhao G, Asseng S, Bindi M, Biernath C, Constantin J, et al Impact of Spatial Soil and Climate Input Data Aggregation on Regional Yield Simulations. PLOS ONE. 2016;11: e0151782 10.1371/journal.pone.0151782 27055028 PMC4824533

[44] Yin Y, Tang Q, Liu X, Zhang X. Water scarcity under various socio-economic pathways and its potential effects on food production in the Yellow River basin. Hydrology and Earth System Sciences. 2017;21: 791–804. 10.5194/hess-21-791-2017

[45] Ostberg S, Schewe J, Childers K, Frieler K. Changes in crop yields and their variability at different levels of global warming. Earth System Dynamics. 2018;9: 479–496. 10.5194/esd-9-479-2018

[46] Blanc É. Statistical emulators of maize, rice, soybean and wheat yields from global gridded crop models. Agricultural and Forest Meteorology. 2017;236: 145–161. 10.1016/j.agrformet.2016.12.022

[47] Leng G. Recent changes in county-level corn yield variability in the United States from observations and crop models. Science of The Total Environment. 2017;607–608: 683–690. 10.1016/j.scitotenv.2017.07.017 28710999

[48] Mistry MN, Wing IS, Cian ED. Simulated vs. empirical weather responsiveness of crop yields: US evidence and implications for the agricultural impacts of climate change. Environ Res Lett. 2017;12: 075007 10.1088/1748-9326/aa788c

[49] Müller C, Elliott J, Chryssanthacopoulos J, Arneth A, Balkovic J, Ciais P, et al Global gridded crop model evaluation: benchmarking, skills, deficiencies and implications. Geosci Model Dev. 2017;10: 1403–1422. 10.5194/gmd-10-1403-2017

[50] Müller C, Elliott J, Kelly D, Arneth A, Balkovic J, Ciais P, et al The Global Gridded Crop Model Intercomparison phase 1 simulation dataset. Scientific Data. 2019;6: 50 10.1038/s41597-019-0023-8 31068583 PMC6506552

[51] Sacks WJ, Deryng D, Foley JA, Ramankutty N. Crop planting dates: an analysis of global patterns. Global Ecology and Biogeography. 2010;19: 607–620. 10.1111/j.1466-8238.2010.00551.x

[52] Mueller ND, Gerber JS, Johnston M, Ray DK, Ramankutty N, Foley JA. Closing yield gaps through nutrient and water management. Nature. 2012;490: 254–257. 10.1038/nature11420 22932270

[53] Williams JR, Jones CA, Kiniry JR, Spanel DA. The EPIC crop growth model. Transactions of the ASAE. 1989;32: 497–0511.

[54] Stockle CO, Williams JR, Rosenberg NJ, Jones CA. A method for estimating the direct and climatic effects of rising atmospheric carbon dioxide on growth and yield of crops: Part I—Modification of the EPIC model for climate change analysis. Agricultural Systems. 1992;38: 225–238. 10.1016/0308-521X(92)90067-X

[55] Izaurralde RC, Williams JR, McGill WB, Rosenberg NJ, Jakas MCQ. Simulating soil C dynamics with EPIC: Model description and testing against long-term data. Ecological Modelling. 2006;192: 362–384. 10.1016/j.ecolmodel.2005.07.010

[56] Izaurralde RC, McGill WB, Williams JR. Development and application of the EPIC model for carbon cycle, greenhouse gas mitigation, and biofuel studies Managing Agricultural Greenhouse Gases. Elsevier; 2012 pp. 293–308.

[57] Kiniry JR, Williams JR, Major DJ, Izaurralde RC, Gassman PW, Morrison M, et al EPIC model parameters for cereal, oilseed, and forage crops in the northern Great Plains region. Canadian Journal of Plant Science. 1995;75: 679–688.

[58] Gaiser T, de Barros I, Sereke F, Lange F-M. Validation and reliability of the EPIC model to simulate maize production in small-holder farming systems in tropical sub-humid West Africa and semi-arid Brazil. Agriculture, Ecosystems & Environment. 2010;135: 318–327. 10.1016/j.agee.2009.10.014

[59] Gassman PW, Williams JR, Benson VW, Izaurralde RC, Hauck LM, Jones CA, et al. Historical development and applications of the EPIC and APEX models. 2004 ASAE Annual Meeting. American Society of Agricultural and Biological Engineers; 2004. p. 1. Available: https://www.card.iastate.edu/products/publications/synopsis/?p=763

[60] Parton WJ, Ojima DS, Cole CV, Schimel DS. A General Model for Soil Organic Matter Dynamics: Sensitivity to Litter Chemistry, Texture and Management. Quantitative Modeling of Soil Forming Processes. 1994;sssaspecialpubl: 147–167. 10.2136/sssaspecpub39.c9

[61] Folberth C, Gaiser T, Abbaspour KC, Schulin R, Yang H. Regionalization of a large-scale crop growth model for sub-Saharan Africa: Model setup, evaluation, and estimation of maize yields. Agriculture, Ecosystems & Environment. 2012;151: 21–33. 10.1016/j.agee.2012.01.026

[62] Balkovič J, van der Velde M, Schmid E, Skalský R, Khabarov N, Obersteiner M, et al Pan-European crop modelling with EPIC: Implementation, up-scaling and regional crop yield validation. Agricultural Systems. 2013;120: 61–75. 10.1016/j.agsy.2013.05.008

[63] Xiong W, van der Velde M, Holman IP, Balkovic J, Lin E, Skalský R, et al Can climate-smart agriculture reverse the recent slowing of rice yield growth in China? Agriculture, Ecosystems & Environment. 2014;196: 125–136. 10.1016/j.agee.2014.06.014

[64] Monteith JL. Evaporation and environment. Symp Soc Exp Biol. 1965 p. 4. 5321565

[65] Hargreaves GH, Samani ZA. Reference crop evapotranspiration from temperature. Applied engineering in agriculture. 1985;1: 96–99.

[66] Rawls WJ, Brakensiek DL. Prediction of soil water properties for hydrologic modeling Watershed management in the eighties. ASCE; 1985 pp. 293–299.

[67] Ray DK, Ramankutty N, Mueller ND, West PC, Foley JA. Recent patterns of crop yield growth and stagnation. Nature Communications. 2012;3: 1293 10.1038/ncomms2296 23250423

[68] United Nations Development Programme. Human Development Report 2016: Human Development for Everyone [Internet]. UN; 2017. 10.18356/b6186701-en

[69] Weedon GP, Balsamo G, Bellouin N, Gomes S, Best MJ, Viterbo P. The WFDEI meteorological forcing data set: WATCH Forcing Data methodology applied to ERA-Interim reanalysis data. Water Resources Research. 2014;50: 7505–7514. 10.1002/2014WR015638

[70] Dee DP, Uppala SM, Simmons AJ, Berrisford P, Poli P, Kobayashi S, et al The ERA-Interim reanalysis: configuration and performance of the data assimilation system. Quarterly Journal of the Royal Meteorological Society. 2011;137: 553–597. 10.1002/qj.828

[71] Schneider U, Becker A, Finger P, Meyer-Christoffer A, Ziese M, Rudolf B. GPCC’s new land surface precipitation climatology based on quality-controlled in situ data and its role in quantifying the global water cycle. Theor Appl Climatol. 2014;115: 15–40. 10.1007/s00704-013-0860-x

[72] Batjes NH. ISRIC-WISE derived soil properties on a 5 by 5 arc minutes global grid. Report 2006/02. Wageningen: ISRIC-WISE derived soil properties on a; 2006.

[73] FAO F. Digital Soil Map of the World. FAO, Rome 1995;

[74] Skalský R, Tarasovičová Z, Balkovič J, Schmid E, Fuchs M, Moltchanova E, et al GEO-BENE global database for bio-physical modeling. GEOBENE project. 2008;

[75] Wösten JHM, Genuchten V, Th M. Using Texture and Other Soil Properties to Predict the Unsaturated Soil Hydraulic Functions. Soil Science Society of America Journal. 1988;52: 1762–1770. 10.2136/sssaj1988.03615995005200060045x

[76] Schaap MG, Bouten W. Modeling water retention curves of sandy soils using neural networks. Water Resources Research. 1996;32: 3033–3040. 10.1029/96WR02278

[77] Potter P, Ramankutty N, Bennett EM, Donner SD. Characterizing the Spatial Patterns of Global Fertilizer Application and Manure Production. Earth Interact. 2010;14: 1–22. 10.1175/2009EI288.1

[78] Portmann FT, Siebert S, Döll P. MIRCA2000—Global monthly irrigated and rainfed crop areas around the year 2000: A new high-resolution data set for agricultural and hydrological modeling. Global Biogeochemical Cycles. 2010;24 10.1029/2008GB003435

[79] Waha K, Bussel LGJ van, Müller C, Bondeau A. Climate-driven simulation of global crop sowing dates. Global Ecology and Biogeography. 2012;21: 247–259. 10.1111/j.1466-8238.2011.00678.x

[80] Porwollik V, Müller C, Elliott J, Chryssanthacopoulos J, Iizumi T, Ray DK, et al Spatial and temporal uncertainty of crop yield aggregations. European Journal of Agronomy. 2017;88: 10–21. 10.1016/j.eja.2016.08.006

[81] RDevelopment Core Team. R: A language and environment for statistical computing. R foundation for statistical computing Vienna, Austria; 2008.

[82] Wickham H. ggplot2: elegant graphics for data analysis. Springer; 2016.

[83] Wei T, Simko V. corrplot: Visualization of a correlation matrix. R package version 073. 2013;230: 11.

[84] Warnes GR, Bolker B, Bonebakker L, Gentleman R, Liaw WHA, Lumley T, et al gplots: various R programming tools for plotting data. R package version 3.0. 1. The Comprehensive R Archive Network 2016;

[85] FAO. FAOSTAT statistical database [Internet]. 2016. Available: https://faostat.fao.org

[86] Folberth C, Yang H, Gaiser T, Abbaspour KC, Schulin R. Modeling maize yield responses to improvement in nutrient, water and cultivar inputs in sub-Saharan Africa. Agricultural Systems. 2013;119: 22–34. 10.1016/j.agsy.2013.04.002

[87] Zhang X, Wu L, Sun N, Ding X, Li J, Wang B, et al Soil CO2 and N2O Emissions in Maize Growing Season Under Different Fertilizer Regimes in an Upland Red Soil Region of South China. Journal of Integrative Agriculture. 2014;13: 604–614. 10.1016/S2095-3119(13)60718-2

[88] Givi J, Prasher SO, Patel RM. Evaluation of pedotransfer functions in predicting the soil water contents at field capacity and wilting point. Agricultural Water Management. 2004;70: 83–96. 10.1016/j.agwat.2004.06.009

[89] Baroni G, Facchi A, Gandolfi C, Ortuani B, Horeschi D, van Dam JC. Uncertainty in the determination of soil hydraulic parameters and its influence on the performance of two hydrological models of different complexity. Hydrol Earth Syst Sci. 2010;14: 251–270. 10.5194/hess-14-251-2010

[90] Kris Van Looy, Johan Bouma, Michael Herbst, John Koestel, Budiman Minasny, Umakant Mishra, et al Pedotransfer Functions in Earth System Science: Challenges and Perspectives. Reviews of Geophysics. 2017;55: 1199–1256. 10.1002/2017RG000581

[91] Gerik T, Williams J, Francis L, Greiner J, Magre M, Meinardus A, et al Environmental Policy Integrated Climate Model-User’s Manual Version 0810. Blackland Research and Extension Center, Texas A&M AgriLife, Temple, USA 2014;

[92] Matthews RB, Pilbeam C. Modelling the long-term productivity and soil fertility of maize/millet cropping systems in the mid-hills of Nepal. Agriculture, Ecosystems & Environment. 2005;111: 119–139. 10.1016/j.agee.2005.06.016

[93] Xiong W, Holman I, Conway D, Lin E, Li Y. A crop model cross calibration for use in regional climate impacts studies. Ecological Modelling. 2008;213: 365–380. 10.1016/j.ecolmodel.2008.01.005

[94] Gavilán P, Lorite IJ, Tornero S, Berengena J. Regional calibration of Hargreaves equation for estimating reference ET in a semiarid environment. Agricultural Water Management. 2006;81: 257–281. 10.1016/j.agwat.2005.05.001

[95] Liu W, Yang H, Liu J, Azevedo LB, Wang X, Xu Z, et al Global assessment of nitrogen losses and trade-offs with yields from major crop cultivations. Science of The Total Environment. 2016;572: 526–537. 10.1016/j.scitotenv.2016.08.093 27552131

[96] Frieler K, Schauberger B, Arneth A, Balkovič J, Chryssanthacopoulos J, Deryng D, et al Understanding the weather signal in national crop-yield variability. Earth’s Future. 2017;5: 605–616. 10.1002/2016EF000525 30377624 PMC6204259

[97] Ewert F, van Ittersum MK, Heckelei T, Therond O, Bezlepkina I, Andersen E. Scale changes and model linking methods for integrated assessment of agri-environmental systems. Agriculture, Ecosystems & Environment. 2011;142: 6–17. 10.1016/j.agee.2011.05.016

[98] Sheahan M, Barrett CB. Ten striking facts about agricultural input use in Sub-Saharan Africa. Food Policy. 2017;67: 12–25. 10.1016/j.foodpol.2016.09.010 28413243 PMC5384438

[99] Eyshi Rezaei E, Siebert S, Ewert F. Impact of data resolution on heat and drought stress simulated for winter wheat in Germany. European Journal of Agronomy. 2015;65: 69–82. 10.1016/j.eja.2015.02.003

[100] Porwollik V, Rolinski S, Heinke J, Müller C. Generating a rule-based global gridded tillage dataset. Earth System Science Data. 2019;11: 823–843. 10.5194/essd-11-823-2019

[101] Zheng B, Campbell JB, Serbin G, Galbraith JM. Remote sensing of crop residue and tillage practices: Present capabilities and future prospects. Soil and Tillage Research. 2014;138: 26–34. 10.1016/j.still.2013.12.009

[102] Hively WD, Lamb BT, Daughtry CST, Shermeyer J, McCarty GW, Quemada M. Mapping Crop Residue and Tillage Intensity Using WorldView-3 Satellite Shortwave Infrared Residue Indices. Remote Sensing. 2018;10: 1657 10.3390/rs10101657

[103] Tao F, Rötter RP, Palosuo T, Díaz‐Ambrona CGH, Mínguez MI, Semenov MA, et al Contribution of crop model structure, parameters and climate projections to uncertainty in climate change impact assessments. Global Change Biology. 2018;24: 1291–1307. 10.1111/gcb.14019 29245185

[104] Deryng D, Sacks WJ, Barford CC, Ramankutty N. Simulating the effects of climate and agricultural management practices on global crop yield. Global Biogeochemical Cycles. 2011;25 10.1029/2009GB003765

[105] Iizumi T, Yokozawa M, Nishimori M. Parameter estimation and uncertainty analysis of a large-scale crop model for paddy rice: Application of a Bayesian approach. Agricultural and Forest Meteorology. 2009;149: 333–348. 10.1016/j.agrformet.2008.08.015

[106] Valade A, Ciais P, Vuichard N, Viovy N, Caubel A, Huth N, et al Modeling sugarcane yield with a process-based model from site to continental scale: uncertainties arising from model structure and parameter values. Geosci Model Dev. 2014;7: 1225–1245. 10.5194/gmd-7-1225-2014

[107] Zhao G, Bryan BA, Song X. Sensitivity and uncertainty analysis of the APSIM-wheat model: Interactions between cultivar, environmental, and management parameters. Ecological Modelling. 2014;279: 1–11. 10.1016/j.ecolmodel.2014.02.003

[108] Wang J, Li X, Lu L, Fang F. Parameter sensitivity analysis of crop growth models based on the extended Fourier Amplitude Sensitivity Test method. Environmental Modelling & Software. 2013;48: 171–182. 10.1016/j.envsoft.2013.06.007

[109] Liu J. A GIS-based tool for modelling large-scale crop-water relations. Environmental Modelling & Software. 2009;24: 411–422. 10.1016/j.envsoft.2008.08.004

[110] Xiong W, Skalský R, Porter CH, Balkovič J, Jones JW, Yang D. Calibration-induced uncertainty of the EPIC model to estimate climate change impact on global maize yield. Journal of Advances in Modeling Earth Systems. 2016;8: 1358–1375. 10.1002/2016MS000625

[111] Gbegbelegbe S, Cammarano D, Asseng S, Robertson R, Chung U, Adam M, et al Baseline simulation for global wheat production with CIMMYT mega-environment specific cultivars. Field Crops Research. 2017;202: 122–135. 10.1016/j.fcr.2016.06.010

[112] Hartkamp AD. Maize production environments revisited: a GIS-based approach. CIMMYT; 2001.

[113] Porter CH, Villalobos C, Holzworth D, Nelson R, White JW, Athanasiadis IN, et al Harmonization and translation of crop modeling data to ensure interoperability. Environmental Modelling & Software. 2014;62: 495–508. 10.1016/j.envsoft.2014.09.004

[114] Hengl T, Jesus JM de, Heuvelink GBM, Gonzalez MR, Kilibarda M, Blagotić A, et al SoilGrids250m: Global gridded soil information based on machine learning. PLOS ONE. 2017;12: e0169748 10.1371/journal.pone.0169748 28207752 PMC5313206

[115] Tóth B, Weynants M, Pásztor L, Hengl T. 3D soil hydraulic database of Europe at 250 m resolution. Hydrological Processes. 2017;31: 2662–2666. 10.1002/hyp.11203

