# Supplementary material for: Parameterization-induced uncertainties and impacts of crop management harmonization in a global gridded crop model ensemble
Source: PLoS One. 2019 Sep 16;14(9):e0221862. doi: 10.1371/journal.pone.0221862 (PMC6746385; doi:10.1371/journal.pone.0221862)
Supplement: S1 File — Text A. Relevant routines of the EPIC model. Text B. Differences between EPIC model versions v0810 and v1102. Text C. Description of EPIC-based GGCMs. Text D. Evaluations of the wider ensemble. Table A. Relevant characteristics of GGCMs in this study based on Müller et al. [30]. Table B. Legend for Table 2 in main paper with brief explanation for parameters differing among EPIC-based GGCMs. Table C. Crop management operations of EPIC-IASA and GEPIC. Table D. Parameterization of different maize cultivars used in the GGCMs as shown in Fig 1 of the manuscript with corresponding coloring of column headings. Table E. Relative spread of maize yield estimates measured as yields of the highest estimate in relation to yields of the lowest estimate in Fig 2 of the main paper. Table F. Statistical coefficients for linear regressions of yield estimates over time in Fig 2 of the main article and in Fig F and mean error [t ha-1] compared to reported yields. Table G. Quantiles of coefficient of variation [%] among EPIC-GGCMs for grid-wise maize yield estimates (Fig 3 in main article) depending on the setup and management scenarios (see Table 1 in main article). Table H. Same as Table G but excluding EPIC-TAMU. Table I. Fractions of grid cells [%] in which the median time series correlation among the EPIC-based GGCMs (Fig 4 in main article) fulfils a certain level of significance. Table J. Same as Table I but excluding EPIC-TAMU. Table K. Time-series correlation coefficient r for each GGCM in the ten major maize producing countries for the fullharm and harm-suffN scenarios (Table 1 in main paper) and annual N fertilizer application rates for maize in each country. Table L. Statistical coefficients for linear regressions of yield estimates (not shown) corresponding to global average yields in Fig V and mean error [t ha-1] compared to reported yields. Table M. Fractions of grid cells [%] in which the median time series correlation among all GGCMs (Fig W) fulfils a certain level of si [file pone.0221862.s001.pdf]

Supplementary Information for "*Parameterization-induced uncertainties and impacts of crop management harmonization in a global gridded crop model ensemble*"

Table of contents

Supplementary Text A-D 1- 8
Supplementary Tables A-P 9-16
Supplementary Figures A-AE 17-41

Text A. Relevant routines of the EPIC model

This sections provides an overview of selected routines of the EPIC model relevant for this study. The descriptions are based on the original documentation of the EPIC model, which is available at http://epicapex.tamu.edu/files/2015/05/EpicModelDocumentation.pdf. Further detail on the soil organic matter routines is provided in ref. [1,2]. Parameter numbers mentioned throughout the section refer to those in Table 2 of the main article and Table B below.

Plant growth, yield formation, and associated stresses

Phenologic development of the crop follows the heat unit (HU) accumulation approach. The average temperature on each day is summed up throughout the growing season until a defined heat unit requirement to reach maturity (potential heat units; PHU) is met. The HUs on a given day are calculated as

$$HU_k = \frac{T_{\max,k} - T_{\min,k}}{2} - T_b \quad \text{(S1)}$$

where HU_k is the heat units accumulated on day k [°C], $T_{max,k}$ and $T_{min,k}$ are the maximum and minimum temperatures on the day [°C], and T_b is the base temperature [°C] as specified in Table D for the maize cultivars used in this study. Potential biomass increase ΔB_p on each day is estimated according to

$$\Delta B_p = 0.001 \times BE \times PAR \quad \text{(S2)}$$

where ΔB_p [t ha^{-1}] is biomass gain, BE [(kg ha^{-1})/(MJ m^{-2})] is a crop-specific biomass-energy-conversion coefficient, and PAR [MJ m^{-2}] is intercepted photosynthetic active radiation depending on LAI, solar radiation, and plant row width (see Table C for examples) according to

$$PAR = 0.5 \times RA \times (1 - e^{-(0.685 - 0.209 \times RIN) \times LAI}) \quad \text{(S3)}$$

where RA [MJ m^{-2}] in incoming solar radiation on a given day, RIN is the row width at planting [-] and LAI is the leaf area index [-] on the respective day.
Biomass gain is constrained mainly by water and nutrient (N and P) deficits as well as temperature and aeration stress. Only the dominant stress on a given day regulates plant growth through a plant growth regulation factor REG ranging from 0 to 1. The sum of the daily values for each stress factor over the growing season is referred to as "stress days" [d].
Water stress (WS) is based on the concept that drought stress is proportional to the transpiration reduction. It is estimated as

$$WS_i = \frac{\sum_{l=1}^{M} u_{l,i}}{PET_i} \quad \text{(S4)}$$

where WS_i is the amount of water stress on day i [-], l is a given soil layer [-], M is the total number of soil layers [-], $u_{l,i}$ is the plant available water in layer l on day i [mm], and PET_i is the potential evapotranspiration on day i [mm]. In addition, water deficit has an impact on HI as described below. Temperature stress (TS) occurs if the average air temperature TG is above the optimum temperature TO or below the base temperature TB on a given day according to

$$TS_i = \sin\left(\frac{\pi}{2} \times \frac{TG_i - TB_i}{TO_i - TB_i}\right) \quad \text{(S5)}$$

or if the average daily temperature exceeds TO by 50%. Nutrient deficits (N stress (NS) and P stress (PS)) vary non-linearly between optimum supply and 50% of the optimum supply when the deficit reaches 100%. First, a scaling factor SNS (here for NS) on a given day i is calculated as

$$SNS_i = 200 \times \left(\frac{UN_i}{cNB_i \times B_i} - 0.5 \right) \quad \text{(S6)}$$

where UN_i is the N uptake on day i [kg ha^{-1}], cNB_i is the optimum N concentration in biomass on day i [kg kg^{-1}] and B_i is the total plant biomass on day i [kg ha^{-1}]. This factor is then used in the estimation of actual nitrogen stress NS according to

$$NS_i = \frac{SNS_i}{SNS_i + \exp(3.52 - 0.026 \times SNS_i)} \quad \text{(S7)}$$

The calculation of PS follows the same pattern. Aeration stress (AS) occurs if the soil humidity approaches water saturation and depends on pore space volume, soil humidity, and a crop-specific sensitivity factor. AS occurs rarely in global simulations, as depth to ground water is typically not considered. It is hence foremost limited to soils prone to water logging such as vertisiols. The plant growth regulation factor REG is finally calculated as

$$REG = \max(WS, TS, NS, PS, AS) \quad \text{(S8)}$$

and subsequently actual biomass gain ΔB_a [t ha^{-1}] as

$$\Delta B_a = \Delta B_p \times REG \quad \text{(S9)}$$

where ΔB_p [t ha^{-1}] is the potential biomass gain on a given day. At maturity, crop yield is calculated by multiplying total aboveground biomass with a water stress-adjusted harvest index (HI_a). HI_a is estimated from simulated potential HI (HI_{max}; depending on HU accumulation) and a defined minimum HI (HI_{min}) according to

$$HI_a = (HI_{max} - HI_{min}) \times \frac{WUR}{WUR + \exp(6.13 - 0.0883 \times WUR)} + HI_{min} \quad \text{(S10)}$$

where WUR is the water use ratio. The fraction of the growing season when water stress starts to limit HI_a is defined in parameter 33. Values for HI_{max} and HI_{min} used in this study are provided in Table D. WUR is estimated at harvest as

$$WUR = 100 \times \frac{\sum_{i=1}^{K} U_i}{\sum_{i=1}^{K} E_{Pi}} \quad \text{(S11)}$$

where U_i [mm d^{-1}] is the actual and E_{Pi} [mm d^{-1}] the potential plant water use rate for day i. K is the total number of days of the growing season.

Biomass accumulation may in addition be limited by root growth stresses (parameter 32), which include soil strength, salinity, and aluminum toxicity. Herein, only one of the EPIC-based GGCMs (EPIC-TAMU) considers these types of stresses.

Hydrology

Daily precipitation is split at the surface into runoff and infiltration. Daily runoff Q [mm] is estimated according to the USDA SCS curve number equation

$$Q = \frac{(R - 0.2s)^2}{R + 0.8s} \quad \text{for } R>0.2s \quad \text{(S12)}$$

where R is precipitation [mm] and s is the retention parameter [mm]. At $R \leq 0.2s$, no runoff occurs. Parameter s depends on the curve number (CN) according to

$$s = 254 \times \frac{100}{CN} - 1 \tag{S13}$$

The CN reflects different types of soils, landuse and management, and are adjusted for slope, surface roughness, and soil humidity in the model. Impacts of these factors on CN as partly calculated dynamically in the model, but includes also various coefficients that need to be determined exogenously. These are among others the CN number index coefficient (parameter 11), which determines the impact of ET on water retention, a CN adjustment factor for plant residue on the field (parameter 12), and the soil variable dependence of CN (parameter 10). Soil humidity is included in the estimation of the retention parameter directly, based on the fraction of field capacity (FC; parameter 9) at present soil humidity. A higher fraction of FC causes higher runoff rates. Percolation from one soil layer l to a deeper soil layer or aquifers starts when soil water storage exceeds FC according to

$$PC_l = (SW_{0,l} - FC_l) \times 1 - e^{(-\Delta t - TT_l)} \tag{S14}$$

where PC_l is the percolation rate, $SW_{0,l}$ is the initial soil water storage, FC_l is the water content at field capacity, Δt is the time interval (here 24h) and TT_l is the travel time through the soil layer. The latter is a function of porosity, field capacity, and saturated conductivity. Also upward movement of soil water may occur if soil layer of low porosity are saturated.
Actual evapotranspiration (ETa) is driven by potential evapotranspiration (PET; parameter 1) and water available for evaporation from soil and transpiration by the crop. EPIC offers five methods for estimating PET, namely Baier-Robertson, Hargreaves, Penman, Penman-Monteith, and Priestly-Taylor. A detailed description and evaluation of the methods in the global EPIC-based GGCM PEPIC is provided in [3]. Various coefficients of the PET estimation can be adjusted manually to local conditions, such as a crop canopy resistance factor for Penman-Monteith, or the linear and exponential coefficients of the Hargreaves method (parameters 2 and 3). ETa is estimated following the approach of Ritchie (1972) with plant transpiration based on leaf area index alone, except for Penman-Monteith, which also considers vapor pressure deficit and canopy resistance. Plant water use throughout the soil profile depends on water demand, root distribution (affected among others by parameters 30 and 31), water availability in each layer, and a water tension function defined by parameter 8. Soil evaporation is the remainder of ETa after transpiration demand is met. Various coefficient regulate soil evaporation, such as parameters 6 and 7, which regulate the evaporation rate with soil depth and parameters 4 and 5, which increase the albedo of soil cover and accordingly decreases the effect of solar radiation on PET estimation if considered by the selected method.

Soil organic matter turnover and nutrient mineralization

Soil organic matter (SOM) and nitrogen (N) cycling follow largely the routines of the CENTURY model [4], which have been adapted for EPIC by Izaurralde et al. [1]. SOM is split into the pools standing dead residue and roots, metabolic and structural litter, slow humus, passive humus, and microbial biomass, which vary in exchange and turnover rates such as the slow to passive humus partitioning coefficient (parameter 23). Mineral and organic C, N and P may leave the system through erosion, leaching and volatilization (C and N). Fluxes between different pools depend on soil and crop management, soil hydrology, temperature, oxygen availability, stoichiometry, directly parameterized coefficients, and the selection of subroutines. The complex routines of OM turnover are laid out in ref [1] in detail.
Field capacity (FC) and wilting point (WP), which differ here most substantially between the soil data used in EPIC-IIASA and GEPIC (Figure C) are key parameters in various soil microbial routines. The soil water control factor SUT for biological processes is estimated according to

$$SUT = \sqrt{\frac{e^{6\times(DB-DB_P)}}{FC - WP}} \tag{S15}$$

where DB is the soil bulk density and DB_P is the bulk density of the plough layer. SUT is limited to a maximum of 1. SUT is part of the combined factor CS for soil microbial activity with

$$CS = \sqrt{CDG \times SUT} \times MAC \times OX \times e^{6\times(DB-DB_P)} \tag{S16}$$

where CDG is a soil temperature coefficient, MAC is the soil microbial activity coefficient (parameter 22), OX is a coefficient for oxygen availability depending on soil depth (modulated by parameters 24 and 25), and the last term reflects the ratio of average soil bulk density and plough layer bulk density as in Eq. S17.

Mineralization from and transformation between pools of N is driven by supply and demand functions as well as transformation coefficients. Net mineralization of N is the differences between gross mineralization (sum of N mineralization from all OM pools) and N immobilized in microbial biomass, depending in turn on the C:N stoichiometry of the biomass. P mineralization is based on the PAPRAN model [5] and depends on the CS factor (Eq. S16) and P concentration in fresh biomass and humus. For both nutrients, the solving of turnover equations follows the law of conservation of mass.

The total volume of nitrification and volatilization is first estimated simultaneously based on NH_3 concentration in the profile, water saturation, pH, soil temperature, and wind sped. The volatilization coefficient is then calculated based on a user specified coefficient (parameter 26), the total nitrification and volatilization volume, and soil layer depth. Denitrification can be estimated by one of three methods. The original EPIC method is based on soil temperature and humidity, total OC, the NO_3 concentration, and a user defined soil humidity threshold and estimates N_2 production only. The more recently introduced Armen Kemanian method considers in addition a respiration factor and produces also N_2O loss estimates without user input requirements. The yet most detailed representation of denitrification processes has been introduced by Cesar Izaurralde and includes production and consumption of O_2, CO_2, and N_2O throughout the soil profile at an hourly time step based on a mechanistic electron transfer model [1].

Dynamic soil handling and degradation

Soil profiles can be handled dynamically or statically (parameter 19). In the latter case, all soil characteristics except pools of mineral nutrients are reset to initial conditions at the beginning of each year. Soil degradation is represented in the form of nutrient and OM depletion in case these constituents are not replenished in sufficient amounts. In addition, the model considers wind and water erosion (parameters 13, 14 and 16) using the WEPS model in the first case – depending on slope, wind speed and field size (parameters 17 and 18) - and providing 6 methods (USLE, RUSLE, modified RUSLE (RUSL2), MUSLE, MUST, MUSS) in the latter case, with differences in driving forces (rainfall or runoff) and assumptions concerning the watershed/field size. Water erosion can be scaled directly, using a water erosion conservation practice factor (parameter 15), which reflects assumptions on farmers practice concerning countermeasures for erosion.

Crop management

The EPIC model allows for a wide range of management specifications from field preparation over planting, fertilizer, manure, pesticide, and irrigation water application, and weeding to harvest and plant residue handling. Fertilizer and irrigation water applications can follow a rigid schedule based on heat unit scheduling or fixed dates or occur automatically based on plant stresses. Triggers (parameters 27 and 29) are set to specific values of the plant growth regulating factor REG (see Eq. S8). E.g. a fertilizer application trigger of 0.9 causes fertilizer application if potential plant growth would be limited on a given day by >10%.

Examples of more detailed operation schedules for two GGCMs are provided in Table C. Besides their direct purpose, field operations are defined by their soil mixing efficiencies, effects on surface roughness, affected soil depth, and ridge height, all of which affect soil OM turnover and hydrologic processes. For planting operations, the initial row width has a direct impact on the estimation of potential plant growth (see Eq. S2).

Text B. Differences between EPIC model versions v0810 and v1102

Two versions of the EPIC field-scale model were used in this study, designated as v0810 and the more recent v1102. The first is the presently publicly available version from the developers at Blackland Research Center of Texas A&M University. The latter has been modified by the developers of the global model framework EPIC-TAMU, mainly with more detailed and revised routines for soil nutrient and carbon cycling. These include gas diffusion routines, root respiration, nutrients in microbial biomass, and improved (de-)nitrification among others. Testing both field-scale models at four sites in differing climate, soil and management conditions shows that the absolute yield levels are at least after a spin-up period mostly at a comparable level and also inter-annual yield

dynamics are mostly very similar (Figure A). The identification of drivers in differences between the models is beyond the scope of this study.

Text C. Description of EPIC-based GGCMs

EPIC-BOKU

EPIC-BOKU (named after home institution University of Natural Resources and Life Sciences (BOKU)) was initially developed to provide yield estimates at contrasting management intensities for land use change and agro-economic models [6–9] at the European and global scales [10–12].
The spatial structure of its input data is based on a regular 5 arcmin grid, which is first aggregated to homogenous response units (HRUs) based on a classification of physical characteristics (elevation, slope, soil). The HRUs are subsequently intersected with administrative units (national borders at the global scale) that determine specific crop management parameters, which are derived from databases or socio-economic data. The field-scale model is run for each of the resulting simulation units (SimU). For comparison with GGCMs running at a 0.5° x 0.5° resolution, the results from the SimUs were resampled based on the pixel-weighted 5 arcmin model outputs per 0.5° x 0.5° grid. Presently, the GGCM runs two nutrient management intensities, high-input and low-input agriculture with accordingly high or low fertilizer application rates. For the default simulations, outputs from the high-input runs were submitted, corresponding to non-nutrient limited yield potential with default growing season assumptions.

EPIC-IIASA

EPIC-IIASA (named after home institution International Institute for Applied Systems Analysis (IIASA)) has been developed in parallel to EPIC-BOKU, partly by the same researchers and shares in principle the same spatial data structure based on HRUs and SimUs whereas simulations can also be run at the 0.5°x0.5° grid cell level. Parameterizations and input data have been adjusted throughout research projects resulting in a substantially differing setup with the major remaining communality being the use of a static soil profile (Table 1 in main manuscript). Growing seasons have been adopted from Sacks et al. [13] and crop-specific spatially explicit N and P application rates from Mueller et al. [14]. Focus regions of recent studies for which model setups have been adjusted are the EU (e.g. [15]) and China [16,17] besides global applications [18,19]. Simulations for the default setup were carried out at the SimU level and the harmonized runs were based on the 0.5° grid.

EPIC-TAMU

EPIC-TAMU (named after home institution Texas A&M University (TAMU)) follows the model development and implementation of EPIC, version 1102, which accounts for C and N stocks and flows in managed terrestrial ecosystems [2]. As in EPIC v. 0810, the coupled C and N model in EPIC-TAMU follows the conceptual pool structure of the Century model [1]. Mineralization and immobilization of C and N also follows the approach in Century but a recent option has been added to describe C and N of microbial biomass following the approach used in the Phoenix model [20]. The EPIC-TAMU version also contains algorithms to model the effects of biochar additions on crop productivity, soil pH, and cation exchange capacity [21]. Other developments include a mechanistic model to describe microbial denitrification and the corresponding feedback on decomposition [2].
EPIC-TAMU has primarily been used for field-scale and regional-scale simulations [22,23]. It has been adapted with minimal changes for use as part of the AgMIP GGCMI project, and has otherwise not been previously used for global simulations. As a result, no default simulations (see Sect. 2.2 in main manuscript) were produced. To keep the number of EPIC-based GGCMs in evaluations across management scenarios constant, the fully harmonized setup was also used as default.

GEPIC

The GEPIC (GIS-based EPIC) GGCM was originally developed for studies of global crop-water relations [24]. In its present version, it uses input data for planting dates, growing season length, P fertilizer application rates, and cultivar distributions besides the original input data elevation, slope, country/region, N fertilizer application rates, and irrigation water management [25]. Default fertilizer inputs are mainly based on the FertiStat database

[26] and have been extrapolated for maize based on the human development index (HDI) for countries lacking data.
More recently, the GGCM has been setup for applications in sub-Saharan Africa based on a regional calibration [25] and thoroughly evaluated in various studies (e.g. [25,27,28]). The authors found that when using dynamic soil profiles in the setup, the model reproduces yields around the year 2000 well after a spin-up of 30 years [25]. Extending the simulation period may result in erosion of the whole soil profile at some point or complete nutrient depletion in grid cells that lack fertilizer inputs. To avoid resulting detrimental effects on crop yields and unrealistically long monocultures, the model is run for each decade of the study period separately, which aims at mimicking fallow rotation with an average cultivation period of 40 years and complete recovery of the soil profile afterwards (see Figure B).

PEPIC

PEPIC (Python-based EPIC) is a global EPIC-based GGCM developed at the Swiss Federal Institute of Aquatic Science and Technology (Eawag) initially based on GEPIC, which had been developed at the same institute. Hence, the two GGCMs have similar features in software design and default input data. However, one of the main purposes of PEPIC was to develop a fully free tool, which can be used without any software license. Therefore, unlike GEPIC, PEPIC was compiled by a free computer language, Python. In addition, the parameterization and setup has been adjusted in large parts (Table 1 in main manuscript) to match focus research purposes. It was initially developed to investigate the impacts of different PET methods on crop-water relations [3]. Presently, applications of PEPIC focus on assessing trade-offs between crop yields and nutrient losses, e.g. N, in the context of global agricultural intensification [29]. For such assessments, N was applied three times during the whole season following a fixed schedule [29]. Besides the parameterization, this is a single major difference compared to the other four EPIC-based GGCMs, which used automatic N fertilization based on plant nutrient requirements.

Text D. Evaluations of the wider ensemble

The members of the GGCM ensemble differ substantially in the implementation and detail of plant growth and agro-environmental processes, stress handling, representation of soils, and fertilizer effects (Table A). Concerning major conceptual plant growth processes, five GGCMs employ dynamic (DA) leaf area development routines and the remainder (incl. the EPIC-based) a prescribed shape (PS) of phenology. Four models use a gross photosynthesis-respiration (P-R) based approach for light utlisation, all others (incl. the EPIC-based) the descriptive radiation use efficiency (RUE) or a mix of both. Beyond these two aspects of plant growth processes, differences among GGCMs increase further for the representation of evapotranspiration, consideration of stresses, yield formation, and soil processes. Only the EPIC-based GGCMs consider at present interactions of soil-crop-management within the ensemble. The other two site-based models APSIM and DSSAT include such routines in principle but these have been disabled for the present ensemble simulations. The ecosystem model-based GGCMs in the ensemble have the least coverage of soil-crop-management interactions including nutrient cycling. In these GGCMs, nutrient supply from exogenous sources is typically considered as a coefficient in plant growth and stress estimation directly (e.g. [30]) or crop parameters are calibrated to match reported yields [31]. Yet, several of these GGCMs have implemented process-based soil nutrient cycling routines in later versions [32,33] that were not available in these simulations. Common to all GGCMs is at least a basic representation of soil hydrology as a component of evapotranspiration and water stress estimates, which can either be based on root zone soil water availability or supply-demand ratio (Table A). Accordingly, despite their modularity in routines for selected agro-environmental processes, the EPIC-based GGCMs cannot be considered representative for structural differences among core models within the ensemble. Parameterization-induced uncertainties within the EPIC-based sub-ensemble can therefore not be generalized for the whole ensemble. However, the evaluation of differences among various EPIC implementations and interactions among setup components can provide valuable insights relevant for future developments of GGCMs within the ensemble and wider community; and relating the EPIC-based sub-ensemble to the wider range of GGCMs provides information about the magnitude of parameterization induced differences compared to structural differences. The wider ensemble employed in this study in turn is representative for major concepts and processes presently implemented in state-of-the-art field-scale maize crop models [34].
For global mean yield estimates, the continuous decrease in the spread among EPIC-based GGCMs with increasing level of harmonization and elimination of nutrient limitations is contrasted by an increasing spread for the non-EPIC-based GGCMs, most notably in the harm-suffN scenario (Figure V, panel b,d,f). This is driven by very high yield estimates by two GGCMs and very low estimates by one, while three simulate yield potentials at

a similar level as the EPIC ensemble. Besides this increase in spread among the GGCMs, the range of yields is in the fullharm scenario among the non-EPIC-based GGCMs about three times as large as among the EPIC-based and more than five times in the harm-suffN scenario. Even if LPJ-GUESS, which provides very low yield estimates in the harm-suffN scenario, was excluded from the analysis, the range in yields for the non-EPIC-based GGCMs would still be about three times that of the EPIC-based sub-ensemble and similar to the spread in the fullharm scenario (not shown). Across scenarios, some non-EPIC-based GGCMs such as pDSSAT show high and increasing yields from default to harm-suffN, while others vary substantially among scenarios, such as PEGASUS, which shows a decrease in yields from default to fullharm, where it provides the lowest estimates, and a substantial increase to harm-suffN, where it ranks second in terms of absolute yields (see also Table L).

Similarly, the correlation of yield estimates from the whole GGCM ensemble at the grid cell level is far lower. While the EPIC-based GGCMs show a significant increase with harmonization and elimination of nutrient deficits (Figure 4a-f), this is less so for the whole ensemble (Figure W; Table M) and not evident for the non-EPIC-based GGCMs alone (Figure Y; Table O). When considering irrigation, the agreement of the whole ensemble is comparably high in cold to temperate climates of the northern hemisphere and extends to parts of the tropics with increasing harmonization (Figure W, panel a,c,e). Under rainfed conditions, the agreement is high in most of the US, Europe and various arid regions and improves with increasing harmonization in the same areas (Figure W, panel b,d,f). The spatial patterns remain largely constant albeit with substantially lower agreement if only the non-EPIC-based GGCMs are considered (Figure Y, panel a-f; Table O). Excluding the models that did not provide simulations for all scenarios results in a slight improvement in model agreement and indicates a larger effect of nutrient deficit elimination than input harmonization (Figure X, Figure Z).

A pair-wise correlation analysis of simulated yields at the pixel level (Figure AA) shows among most GGCMs a fairly even distribution of correlation coefficients across management scenarios with the mode around zero. Most notably this is the case for two ecosystem model-based GGCMs CLM-crop and PEGASUS, although the peak of the latter moves to a high correlation in the harm-suffN scenario with sufficient irrigation for most EPIC-based GGCMs. The ecosystem model-based ORCHIDEE-crop shows a good agreement with various GGCMs, foremost EPIC-IIASA, EPIC-TAMU, and PEPIC in the fullharm simulations (no harm-suffN simulations were provided by ORCHIDEE-crop for maize), although this model differs substantially in the representation of plant growth, associated stresses, and representation of soil processes (Table A).

The evaluation of model skill in reproducing inter-annual variability for the top producing countries in the harmonized scenarios shows that for the majority of countries and models the highest skill can be achieved with the harm-suffN setup (Figure AB). Notable exceptions occur e.g. for pDSSAT in France and CLM-crop in Brazil. Various EPIC-based GGCMs (n=4) appear among the best performing GGCMs with a slightly higher share (n=6) from the wider ensemble. With the whole ensemble, significant improvements occur foremost for countries in which the EPIC-based GGCMs show low to moderate skill, such as Brazil and Mexico, while performance remains low for Indonesia across the whole ensemble.

Grouping the GGCMs by the binary conceptual characteristics of model type, leaf area development, and light utilization (Table A) to assess the impact of key structural differences on reproducing inter-annual variability of global average yields (corresponding to Figure V) indicates that the implementation of light utilization as such within the ensemble has little impact on time-series correlation coefficient r (Figure ACd-f). For leaf area development there is typically higher skill for models using the PS approach (Figure ACa-c), whereas grouped by model types, the GGCMs using site-based core models show higher skill (Figure ACg-i). Yet, there is an imbalance in sample sizes between the groups and the five EPIC-based GGCMs dominate the respective groups they belong to. Accordingly, due to the small sample sizes, the multiple implementations of one core model, and wide-ranging structural differences, these evaluations can only be considered a first indication about the contribution of structural differences to GGCM skills. It will need to be determined in future experiments in how far whole GGCM structure or single components contribute to higher skill. Finally, the outlier in the harmonized scenarios for the groups PS, RUE, and site-based models (Figure AC, panels b,c,e,f,h,i), which is caused by an EPIC-based GGCM, indicates that the GGCM-specific setup can play a substantial role besides structure. Hence, further evaluations across scales and for contrasting sites will be required to identify structural model components that provide higher skill as has recently been done for temperature response functions in site-based models [35].

Comparing directly the EPIC-based to the wider ensemble for the same metric time-series correlation coefficient r (Figure AD) shows that the individual EPIC-based GGCMs have on average higher skill in the default and harm-suffN setups but not fullharm, for which they are still within the inter-quartile range of the non-EPIC-based sub-ensemble. The percentile ranges of the two sub-ensembles are highly comparable in the default scenario. However, they widen for the non-EPIC-based and whole ensemble with increasing harmonization, but remain constant for the EPIC-based sub-ensemble although an outlier occurs here in the harmonized scenarios. The performance of

the multi-GGCM mean (MGM; arithmetic mean of yields from all GGCMs in a (sub)-ensemble plotted as cross marks in Figure AD) is higher for the whole ensemble than the two sub-ensembles in the default and harm-suffN setups and overall highest in the default scenario. In the fullharm setup, the MGM of the non-EPIC-based GGCMs shows the highest skill. Accordingly, although the single EPIC-based GGCMs show overall high skill, their MGM is consistently the lowest, albeit it nearly equals that of the non-EPIC-based models in the default setup.

To also test the sensitivity of MGM in each of these ensembles to the exclusion of single GGCMs, we calculated MGM in addition with exclusion of one GGCM at a time (Figure AE). This shows that the EPIC-based ensemble is in the harmonized setups most sensitive to the selection of GGCMs, while the whole ensemble is highly resilient and the non-EPIC-based ensemble is least sensitive in the fullharm scenario. In addition, MGM is most sensitive to the setup scenarios for the EPIC-based GGCMs.

In summary, the evaluations of ensemble performance suggest that MGM can profit more from the combination of structurally different models rather than various configurations of the same core model. As for absolute yields (Figure V), structural differences also cause a greater spread in time-series correlation coefficients in the non-EPIC-based ensemble for the harmonized scenarios. Albeit, an outlier among the EPIC-based GGCMs indicates that the specific setup greatly affects GGCM performance. Yet, it needs to be determined in future experiments in how far these results are driven by sample sizes.

Table A. Relevant characteristics of GGCMs in this study based on Müller et al. [36].

GGCM	Type[1]	Leaf area development[2]	Light utilisation[3]	Evapotrans-piration[4]	Growth stresses[5]	Yield formation[6]	Soil input data[7]	Soil layers[8]	Soil C and nutrient models[9]	Crop residue handling	Spin-up[10]
**CLM-crop**	Eco	DA	P-R	TF	W (S),N,H	Prt	IGBP Global Soil Data Task 2000	10	C N	To litter pool	NA
**EPIC-BOKU**	Site (EPIC)	PS	RUE	PM	W (E), T, H, A, N, P	HIws Prt B	ISRIC-WISE; ROSETTA; AWC; Albedo D; HYD USDA	10	C N B(1) P(6)	To litter pool	Soil OM, C, NH3, NO3, H2O, P (1)
**EPIC-IIASA**	Site (EPIC)	PS	RUE	HG	W (E), T, H, A, N, P	HIws Prt B	ISRIC-WISE; ROSETTA; AWC; HYD USDA	10	C N B(1) P(6)	To litter pool	Soil OM, C, NH3, NO3, H2O, P (50)
**EPIC-TAMU**	Site (EPIC)	PS	RUE	PM	W (E), T, H, A, N, P	HIws Prt B	ISRIC-WISE	3	C N B(1) P(6)	To litter pool	Soil OM, C, NH3, NO3, H2O, P, CR (10)
**GEPIC**	Site (EPIC)	PS	RUE	HG	W (E), T, H, A, N, P	HIws Prt B	ISRIC-WISE	5	C N B(1) P(6)	80% removed 20% litter	Soil OM, C, NH3, NO3, H2O, P, CR (30)
**LPJ-GUESS**	Eco	DA	P-R	PT	W (S), T	HIws	HWSD; STC; HYD C; THM L	2	NA	Removed, no effect on yield	H2O (30)
**LPJmL**	Eco	PS	P-R	PT	W (S), T	HIws	HWSD; STC; HYD C; THM L	5	NA	Removed, no effect on yield	H2O, Tsoil (200)
**ORCHIDEE-crop**	Eco	DA	P-R	PT	W (S),T,N	Prt	NA	11	NA	Removed, no effect on yield	H2O (1)
**pAPSIM**	Site	DA	RUE	TE	W (E,S), T, H, A, N	Gn Prt	HWSD	5	C,N,P,B(3)	NA	NA
**pDSSAT**	Site	PS	RUE	PT/PM	W (E), T, H, A, N	Gn	HWSD	4	C N P(3)	Removed, no effect on yield	Soil OM, C, NH3, NO3, H2O (1)
**PEGASUS**	Eco	DA	RUE	PT	W (E), T, H, N, P, K	Prt	ISRIC-WISE (AWC)	3	NA	NA	H2O (4)
**PEPIC**	Site (EPIC)	PS	RUE	PM	W (E), T, H, A, N, P	HIws Prt B	ISRIC-WISE	5	C N B(1) P(6)	Yes	Soil OM, C, NH3, NO3, H2O, P, CR (20)

[1] Site: site-base crop model; Eco: ecosystem model
[2] DA: Dynamic simulation based on development and growth processes; PS: prescribed shape of LAI curve as function of phenology, modified by water stress & low productivity
[3] RUE: Simple (descriptive) radiation use efficiency approach; P-R: Detailed (explanatory) gross photosynthesis-respiration
[4] TF: Turbulent Flux, PM: Penman-Monteith, HG: Hargreaves, PT: Priestly-Taylor, TE: Transpiration Efficiency
[5] W: water stress with (S)=water available in root zone and (E)=ratio of supply to demand; T: temperature stress; H: specific heat stress; A: aeration stress; N: nitrogen stress; P: phosphorus stress; K: potassium stress; BD: bulk density; AL: aluminum stress
[6] Yield formation depending on: HI: fixed harvest index; B: total (above ground) biomass; Gn: number of grains and grain growth rate; Prt: partitioning during reproductive stages; HIws: harvest index modified by water stress
[7] Major source of soil property input data and methods for manipulation to derive parameters required by the model; Albedo D: Albedo according to Dobos, 2006; AWC: Available Water Capacity (Van Genuchten et al., 1992) ; HYD USDA: hydraulic soil parameters according to USDA and NRCS, 2015; HYD C: hydraulic soil parameters according to Cosby et al., 1984; THM L: thermal parameters according to Lawrence and Slater, 2008; HWSD: Harmonized world soil database (Fischer et al., 2008); STC: soil texture classification based on the USDA soil texture classification (http://ufdc.ufl.edu/IR00003107/00001); ISRIC-WISE (Batjes, 2006) ; ROSETTA (Schaap and Bouten, 1996)
[8] Number of soil layers considered in simulation
[9] C: carbon/organic matter model; N: nutrient cycling model; P(x): x number of organic matter pools; B(x): x number of microbial biomass pools; NA: no soil C and nutrient cycling or no effect on crop growth
[10] Modules affected by spin-up: OM: organic matter, C: carbon; NH3: ammonia; NO3: nitrate; H2O: soil water; P: phosphorus; CR: crop residues; (X) number of spin up years; NA: no spin-up

Table B. Legend for Table 2 in main paper with brief explanation for parameters differing among EPIC-based GGCMs. Numbers in round braces refer to the number of subroutines available for estimating the respective model output. Routines considering the parameters are laid out in Text A.

	No	Parameter	Description/Effect
Hydrology	1	PET estimation method (5)	Affects PET and hence water stress and hydrology
	2	Hargreaves exp. coefficient	Higher values increase importance of diurnal temperature range in Hargreaves equation
	3	Hargreaves linear coefficient	Linearly scales PET estimation in Hargreaves eq.
	4	Soil evaporation-cover coefficient	Affects evaporation from soil through surface albedo
	5	Soil cover-temperature function	Defines two points on a sigmoid curve describing soil cover effect on soil temperature
	6	Soil evaporation coefficient	Higher values increase soil evaporation from top 0.2m in exponential term
	7	Soil evaporation-depth function	Defines two points on a sigmoid curve describing evaporation from soil with depth
	8	Plant water use-soil water tension function	Defines two points on a sigmoid curve describing plant water use in relation to soil water tension
	9	Field capacity and wilting point estimation method (11)	Affects wide range of soil hydrologic and microbial processes by defining soil water holding capacity and saturation and a given water content
	10	Soil variable dependence of daily curve number (CN) estimate (5)	Daily CN estimates may depend on soil water with or without weighting by depth or be static
	11	CN number index coefficient	Regulates impact of PET in runoff retention parameter. Higher values increase runoff
	12	CN adjustment for standing dead residue	Higher values increase runoff if residue<1 t ha^{-1} and increase if >1 t ha^{-1}
Soil degradation	13	Wind erosion considered	Loss of top soil and total soil volume
	14	Water erosion considered	Loss of top soil and total soil volume
	15	Water erosion conservation practice	Lower values proportionally limit water erosion
	16	Water erosion estimation method (6)	Magnitude of soil erosion and differences in driving factors (e.g. rainfall force vs. runoff)
	17	Field length for wind erosion	Higher values increase wind erosion
	18	Field width for wind erosion	Higher values increase wind erosion
	19	Soil profile handling (static/dynamic)	Static soil profile is reinitialized at the beginning of each year, except for mineral nutrient pools
	20	Simulation continuity (transient/decadal)	Simulations are carried in a fully transient way or as in the GEPIC model separately for each decade
Organic matter and nutrient cycling	21	Denitrification method (3)	Affects magnitude and dynamics of denitrification
	22	Microbial decay rate	Higher value increases microbial turn-over off labile C pool
	23	Slow to passive humus coefficient	High values allocate more slow to passive humus, resulting in slower nutrient mobilization from OM
	24	Oxygen content-soil depth function	Defines two points on a sigmoid curve describing oxygen distribution with soil depth together with parameter 20 below
	25	Oxygen coefficient for microbial activity	Higher values decrease O_2 avail. with soil depth
	26	N volatilization coefficient	Fraction of potential nitrification and volatilization of NH_3 allocated to volatilization
Management	27	Automatic irrigation trigger	Potential biomass reduction factor on a given day that triggers application of irrigation water (e.g. 0.9 corresponds to reduction by ≥10%)
	28	Maximum single water application [mm]	Max. volume of water applied per irrigation event
	29	Automatic fertilizer application trigger[4)]	Value of potential biomass reduction factor on a given day that triggers application of fertilizer
Growth	30	Coefficient for lin. or exp. root growth	Allocates root growth to lin. and exp. functions
	31	Coefficient for root growth dist. by depth	Higher values increase root growth with soil depth
	32	Root growth stress considered	Higher values render root growth less sensitive to bulk density constraints (≥2 eliminates constraint)
	33	Fraction of growing season from which HI_{min} affects yield formation	Fraction of growing season in terms of PHU from which on water availability affects yield formation

Table C. Crop management operations of EPIC-IASA and GEPIC.

Operation	EPIC-IIASA	GEPIC
Fertilizer application[1]	Fixed P	Fixed P
Ploughing	Moldboard plough<br>Tillage depth=150 mm<br>Surface roughness=30 mm<br>Mixing efficiency=99%	Tandem disk<br>Tillage depth=40 mm<br>Surface roughness=50 mm<br>Mixing efficiency=75%
Planting	Regular planter<br>Tillage depth=40 mm<br>Surface roughness=10 mm<br>Mixing efficiency=10%<br>Row spacing=1.0 m<br>Ridge height=75 mm	Regular planter<br>Tillage depth=20 mm<br>Surface roughness=10 mm<br>Mixing efficiency=10%<br>Row spacing=0.5 m<br>Ridge height=75 mm
In-season operations		2x row cultivation (at 10% and 25% PHU accumulated)<br>Tillage depth=25 mm<br>Surface roughness=15 mm<br>Mixing efficiency=25%
Harvest	Combined harvester	Combined harvester
Residue handling	No residue removal	Removal of approx. 80% stover

[1]N fertilizer is applied automatically in both models based on plant stress as specified in Table 2 of the main article

Table D. Parameterization of different maize cultivars used in the GGCMs as shown in Figure 1 of the manuscript with corresponding coloring of column headings. Cultivar 1 is the default in the EPIC model and corresponds to a high-yielding variety. Cultivar 2 has been calibrated for applications in Europe **[37]**. Cultivar 3 is a faster maturing version of Cultivar 1. Cultivar 4 has been parameterized for West Africa and North-Eastern Brazil **[38]**. TBS=base temperature for plant growth, TOP=optimum temperature, HI_{max}=maximum harvest index without water stress, HI_{min}=minimum harvest index under water stress. The temperature requirement (GDD), commonly used for distinguishing cultivars, is here prescribed by the growing season length of the spatially explicit planting and harvest dates.

Parameter	Cutivar1	Cultivar1b	Cutivar2	Cutivar3	Cutivar4
TBS [°C]	8	8	6.5	8	8
TOP [°C]	25	25	22.5	25	25
HI_{max} [-]	0.5	0.55	0.5	0.5	0.35
HI_{min} [-]	0.4	0.4	0.4	0.4	0.01

Table E. Relative spread of maize yield estimates measured as yields of the highest estimate in relation to yields of the lowest estimate in Figure 2 of the main paper. See Table 1 of the main paper for management scenarios.

Management	Relative range of maize yield estimates	
	Maximum [%]	Mean [%]
default	124	95
fullharm	55	41
harm-suffN	26	18

Table F. Statistical coefficients for linear regressions of yield estimates over time in Figure 2 of the main article and in Figure F and mean error [t ha^{-1}] compared to reported yields. The default scenario for EPIC-TAMU is replaced by fullharm as the first scenario was not simulated by this GGCM (see methods).

Model	Intercept	Slope	R^2	p	Mean error	Scenario and panel in Figure 2 and Figure F
EPIC-BOKU	8.068	-0.001	0.000	0.909	3.676	default panel a
EPIC-IIASA	6.178	0.006	0.114	0.068	1.891	
EPIC-TAMU	5.454	-0.005	0.045	0.258	0.987	
GEPIC	4.128	0.001	0.001	0.901	-0.246	
PEPIC	5.100	-0.018	0.376	0.000	0.439	
EPIC-BOKU	6.861	-0.039	0.660	0.000	1.880	fullharm panel b
EPIC-IIASA	6.399	0.005	0.073	0.150	2.096	
EPIC-TAMU	5.454	-0.005	0.045	0.258	0.987	
GEPIC	4.611	-0.001	0.001	0.896	0.216	
PEPIC	5.162	-0.017	0.314	0.001	0.508	
EPIC-BOKU	7.409	-0.063	0.823	0.000	2.049	harm-suffN panel c
EPIC-IIASA	6.747	0.006	0.075	0.143	2.450	
EPIC-TAMU	6.773	0.007	0.064	0.178	2.501	
GEPIC	5.895	0.005	0.026	0.395	1.596	
PEPIC	6.070	-0.015	0.218	0.009	1.459	

Table G. Quantiles of coefficient of variation [%] among EPIC-GGCMs for grid-wise maize yield estimates (Figure 3 in main article) depending on the setup and management scenarios (see Table 1 in main article). Table H shows results without EPIC-TAMU, for which default and fullharm are identical.

Management scenario	Irrigation regime	0%	25%	50%	75%	100%
default	irrigated	5.14	23.71	44.60	73.58	223.61
default	rainfed	4.34	30.43	52.45	76.83	223.61
fullharm	irrigated	2.49	22.48	39.06	54.05	223.61
fullharm	rainfed	2.89	28.42	44.21	62.31	223.61
harm-suffN	irrigated	3.22	18.02	24.90	33.32	223.61
harm-suffN	rainfed	4.38	20.31	27.75	44.78	223.61

Table H. Same as Table G but excluding EPIC-TAMU.

Management scenario	Irrigation regime	0%	25%	50%	75%	100%
default	irrigated	1.02	24.06	47.84	78.76	200.00
default	rainfed	2.45	28.83	56.41	82.54	200.00
fullharm	irrigated	1.23	24.04	42.52	59.52	200.00
fullharm	rainfed	1.77	26.89	48.03	68.11	200.00
harm-suffN	irrigated	0.90	13.84	21.18	30.39	200.00
harm-suffN	rainfed	1.26	15.85	25.39	46.25	200.00

Table I. Fractions of grid cells [%] in which the median time series correlation among the EPIC-based GGCMs (Figure 4 in main article) fulfils a certain level of significance. Results without EPIC-TAMU, for which default and fullharm are identical, are shown in Table J.

Management scenario	Water supply	p<0.1	p<0.05	p<0.01
default	irrigated	16.87	11.21	3.47
default	rainfed	36.61	28.20	13.26
fullharm	irrigated	23.79	17.21	8.95
fullharm	rainfed	48.81	40.44	24.64
harm-suffN	irrigated	68.76	62.68	50.76
harm-suffN	rainfed	68.29	60.35	43.30

Table J. Same as Table I but excluding EPIC-TAMU.

Management scenario	Water supply	p<0.1	p<0.05	p<0.01
default	irrigated	14.44	9.44	3.31
default	rainfed	28.83	21.43	9.68
fullharm	irrigated	22.58	17.09	9.29
fullharm	rainfed	44.58	36.48	22.32
harm-suffN	irrigated	56.71	49.54	36.44
harm-suffN	rainfed	59.32	50.65	33.52

Table K. Time-series correlation coefficient r for each GGCM in the ten major maize producing countries for the fullharm and harm-suffN scenarios (Table 1 in main paper) and annual N fertilizer application rates for maize in each country. In the harm-suffN scenario, sufficient N is applied in all countries.

Country	Best	EPIC-BOKU	EPIC-IIASA	GEPIC	EPIC-TAMU	PEPIC	Country median	N rate [kg ha^{-1}]	
United States	0.878	0.737	0.878	0.707	0.757	0.772	0.757	163	fullharm
China	0.711	0.711	0.388	0.549	0.594	0.563	0.563	186	
Brazil	0.331	0.293	0.224	0.331	0.247	0.036	0.247	61	
Argentina	0.796	0.234	0.796	0.349	0.564	0.567	0.564	31	
Mexico	0.328	0.085	0.067	0.216	0.302	0.328	0.216	120	
India	0.777	0.246	0.777	0.395	0.411	0.368	0.395	35	
Ukraine	0.550	0.142	0.308	0.413	0.463	0.550	0.413	22	
Indonesia	0.111	0.019	0.111	0.056	0.082	0.021	0.056	55	
France	0.848	0.786	0.716	0.782	0.832	0.848	0.786	201	
South Africa	0.622	0.513	0.622	0.609	0.508	0.507	0.513	70	
**GGCM median**	**0.666**	**0.269**	**0.505**	**0.404**	**0.486**	**0.528**	-	-	
United States	0.870	0.729	0.870	0.724	0.767	0.769	0.767	sufficient	harm-suffN
China	0.710	0.710	0.391	0.549	0.582	0.564	0.564	sufficient	
Brazil	0.381	0.278	0.315	0.351	0.381	0.238	0.315	sufficient	
Argentina	0.787	0.409	0.787	0.672	0.756	0.647	0.672	sufficient	
Mexico	0.270	0.270	0.099	0.192	0.153	0.232	0.192	sufficient	
India	0.809	0.338	0.809	0.550	0.544	0.594	0.550	sufficient	
Ukraine	0.490	0.135	0.316	0.408	0.388	0.490	0.388	sufficient	
Indonesia	0.097	0.063	0.087	0.002	0.049	0.097	0.063	sufficient	
France	0.849	0.789	0.720	0.826	0.832	0.849	0.826	sufficient	
South Africa	0.690	0.432	0.690	0.678	0.664	0.557	0.664	sufficient	
**GGCM median**	**0.700**	**0.373**	**0.540**	**0.550**	**0.563**	**0.560**	-	-	

Table L. Statistical coefficients for linear regressions of yield estimates (not shown) corresponding to global average yields in Figure V and mean error [t ha^{-1}] compared to reported yields. The default scenario for EPIC-TAMU is replaced by fullharm, the fullharm scenario for LPJmL and LPJ-GUESS by default as the respective scenarios were not simulated by these GGCMs (see Methods).

Model	Intercept	Slope	R^2	p	Mean error	Scenario and panels in Figure 2 and Figure V
EPIC-BOKU	8.068	-0.001	0.000	0.909	3.676	default<br>Panel a<br>Panels a,b
EPIC-IIASA	6.178	0.006	0.114	0.068	1.891	
EPIC-TAMU	5.454	-0.005	0.045	0.258	0.987	
GEPIC	4.128	0.001	0.001	0.901	-0.246	
PEPIC	5.100	-0.018	0.376	0.000	0.439	
CLM-crop	6.839	0.014	0.260	0.004	2.675	
LPJmL	4.557	0.007	0.044	0.266	0.277	
LPJ-GUESS	3.585	0.004	0.110	0.073	-0.738	
pAPSIM	5.245	0.003	0.040	0.286	0.915	
pDSSAT	7.358	0.001	0.001	0.864	2.993	
PEGASUS	4.908	0.020	0.254	0.004	0.834	
EPIC-BOKU	6.861	-0.039	0.660	0.000	1.880	fullharm<br>Panel b<br>Panels c,d
EPIC-IIASA	6.399	0.005	0.073	0.150	2.096	
EPIC-TAMU	5.454	-0.005	0.045	0.258	0.987	
GEPIC	4.611	-0.001	0.001	0.896	0.216	
PEPIC	5.162	-0.017	0.314	0.001	0.508	
CLM-crop	6.758	0.001	0.003	0.786	2.393	
LPJmL	4.557	0.007	0.044	0.266	0.277	
LPJ-GUESS	3.585	0.004	0.110	0.073	-0.738	
pAPSIM	5.511	0.003	0.018	0.476	1.170	
pDSSAT	8.044	0.001	0.000	0.912	3.674	
PEGASUS	2.724	0.013	0.392	0.000	-1.454	
EPIC-BOKU	7.409	-0.063	0.823	0.000	2.049	harm-suffN<br>Panel c<br>Panels e,f
EPIC-IIASA	6.747	0.006	0.075	0.143	2.450	
EPIC-TAMU	6.773	0.007	0.064	0.178	2.501	
GEPIC	5.895	0.005	0.026	0.395	1.596	
PEPIC	6.070	-0.015	0.218	0.009	1.459	
CLM-crop	6.850	0.014	0.263	0.004	2.689	
LPJmL	6.063	0.003	0.008	0.634	1.720	
LPJ-GUESS	2.523	0.009	0.538	0.000	-1.719	
pAPSIM	7.261	-0.001	0.000	0.920	2.869	
pDSSAT	10.064	0.003	0.003	0.782	5.733	
PEGASUS	9.783	0.022	0.226	0.008	5.741	

Table M. Fractions of grid cells [%] in which the median time series correlation among all GGCMs (Figure W) fulfils a certain level of significance. Evaluations excluding EPIC-TAMU, LPJmL, and LPJ-GUESS, for which default and fullharm are identical, are shown in Table N.

Management scenario	Water supply	$p<0.1$	$p<0.05$	$p<0.01$
default	irrigated	4.99	2.40	0.31
default	rainfed	9.34	4.86	0.68
fullharm	irrigated	3.51	2.03	0.42
fullharm	rainfed	12.29	6.80	1.13
harm-suffN	irrigated	7.06	3.64	0.82
harm-suffN	rainfed	15.31	8.76	1.60

Table N. Same as Table M but excluding EPIC-TAMU, LPJmL, and LPJ-GUESS.

Management scenario	Water supply	p<0.1	p<0.05	p<0.01
default	irrigated	4.92	2.13	0.24
default	rainfed	8.97	4.92	0.94
fullharm	irrigated	3.56	1.97	0.33
fullharm	rainfed	12.21	7.22	1.97
harm-suffN	irrigated	10.63	5.47	1.08
harm-suffN	rainfed	19.87	12.74	3.70

Table O. Fractions of grid cells [%] in which the median time series correlation among the GGCMs, excluding the EPIC-based ones, (Figure Y) fulfils a certain level of significance. Evaluations excluding LPJmL and LPJ-GUESS, for which default and fullharm are identical, are shown in Table P.

Management scenario	Water supply	p<0.1	p<0.05	p<0.01
default	irrigated	4.30	2.26	0.42
default	rainfed	3.90	1.93	0.34
fullharm	irrigated	3.66	1.95	0.42
fullharm	rainfed	3.47	1.39	0.17
harm-suffN	irrigated	3.19	1.95	0.49
harm-suffN	rainfed	3.96	1.80	0.27

Table P. Same as Table O but excluding LPJmL and LPJ-GUESS.

Management scenario	Water supply	p<0.1	p<0.05	p<0.01
default	irrigated	5.79	2.92	0.39
default	rainfed	5.34	2.91	0.81
fullharm	irrigated	4.62	2.15	0.30
fullharm	rainfed	4.59	2.14	0.37
harm-suffN	irrigated	5.88	2.89	0.45
harm-suffN	rainfed	7.40	4.03	0.92

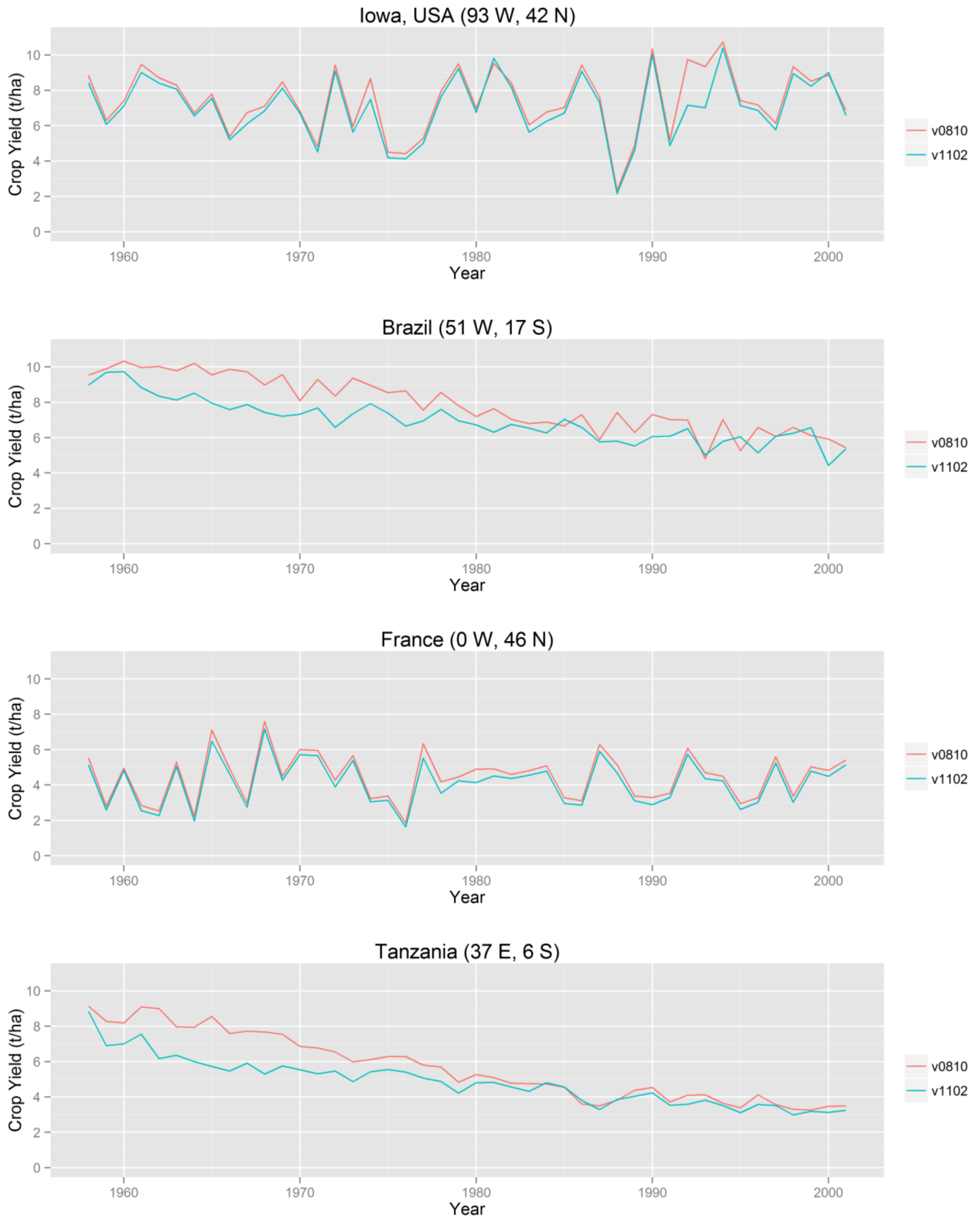

Figure A. Maize yield estimates of EPIC v0810 and EPIC v1102 for four contrasting locations.

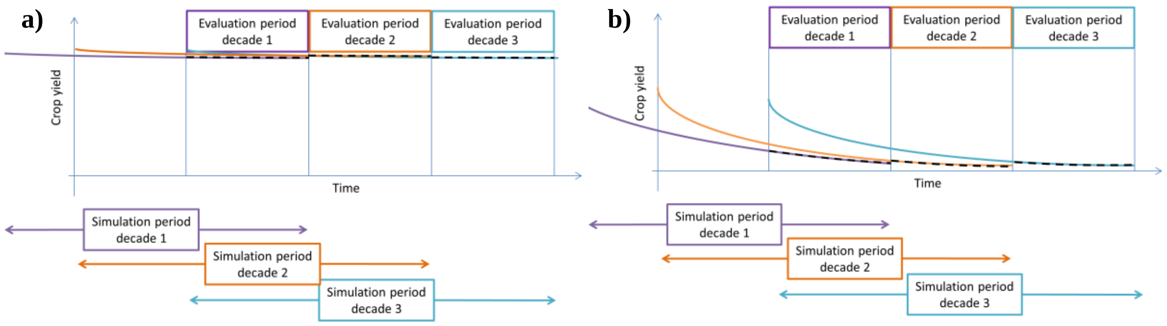

Figure B. Schematic representation of decadal GEPIC runs with dynamic soil profile and erosion for (a) high nutrient input and (b) low nutrient input conditions. Colors represent simulations for three decades with a 20 year spin-up for each decade, which is discarded. Only the last ten years are part of the evaluation as indicated by the dashed black lines.

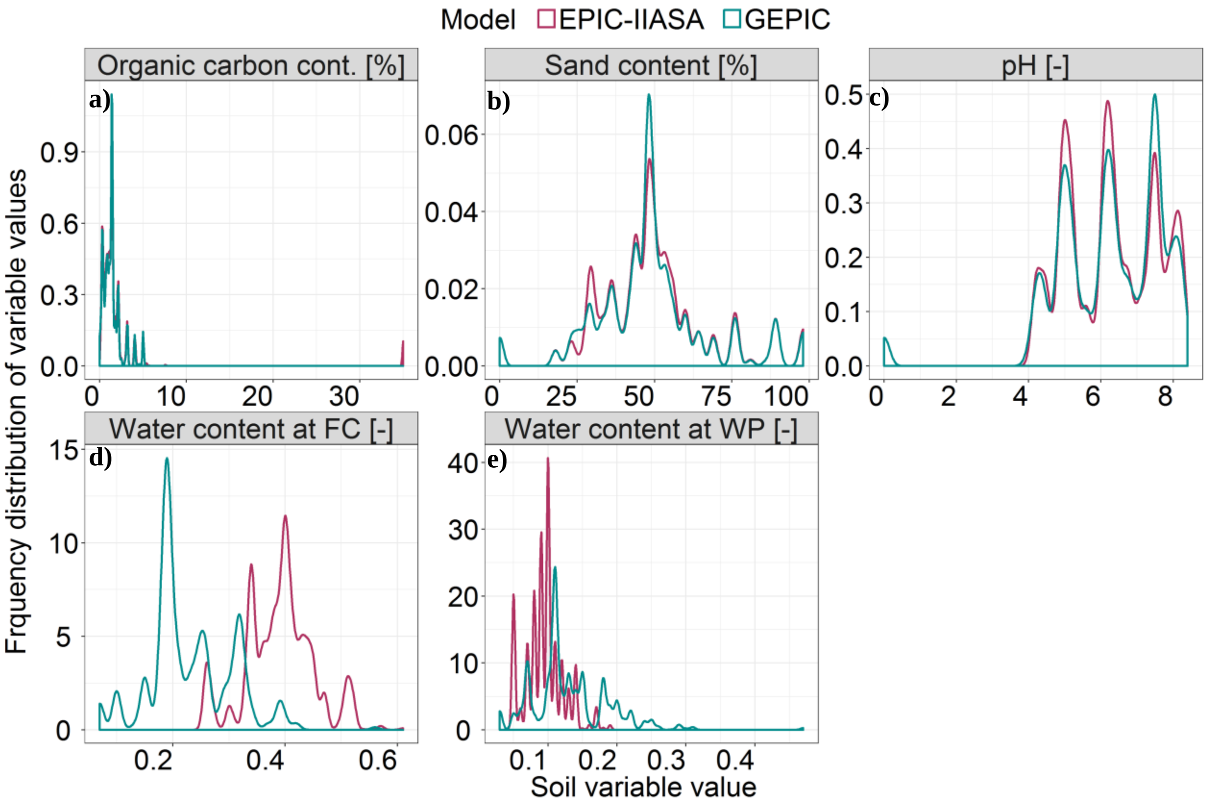

Figure C. Density distributions of key soil parameters in the original ISRIC WISE dataset used in GEPIC and the processed soil data used in EPIC-IIASA based on WISE. Water contents [-] at field capacity (FC) and wilting point (WP) are not provided in the original dataset and were estimated by different methods as specified in Table 2 (parameter 6) of the main paper. Very low pH values for WISE indicate the coincidence of water bodies with the land mask. Organic soil such as histosols do not exist in the original soil data and were parameterized for EPIC-IIASA only as indicated by the high OC values occurring in this dataset.

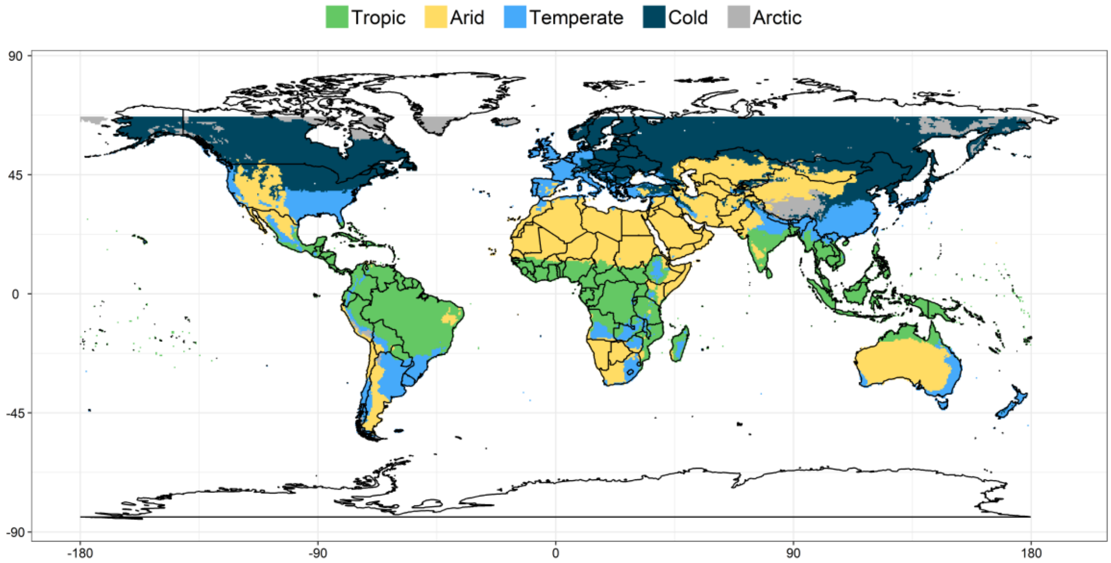

Figure D. Major Koeppen-Geiger climate regions according to Peel et al. [39] based on the climate data used in this study. Climate data were available only up to 67°N above which no harvest area for maize is reported.

a)

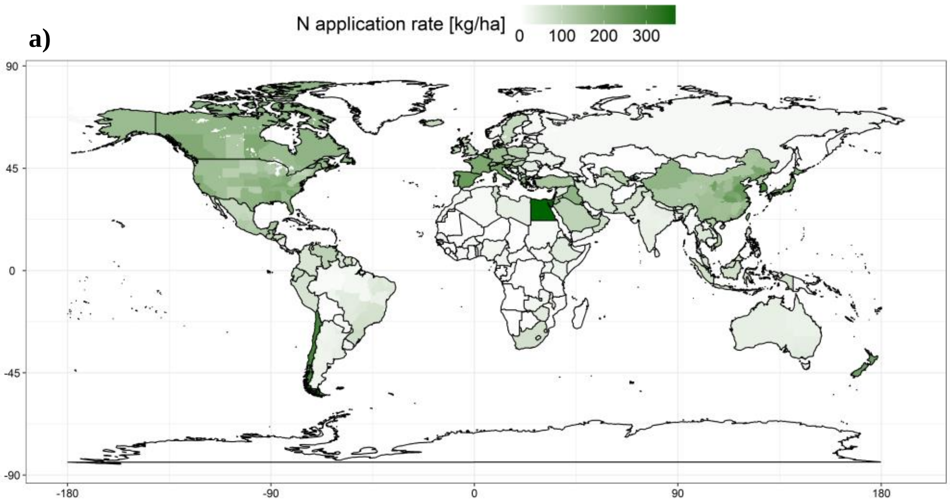

b)

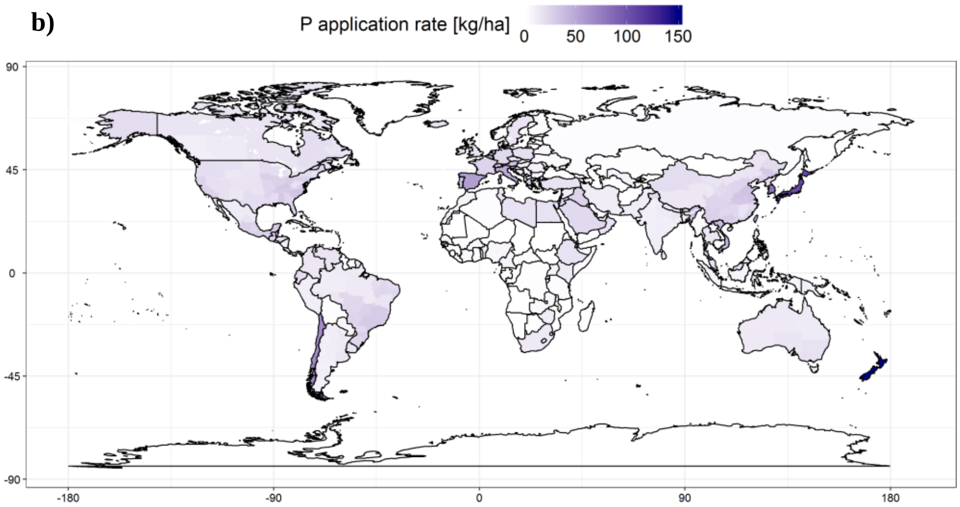

Figure E. (a) Nitrogen and (b) phosphorus fertilizer application rates used in the fullharm setup [40].

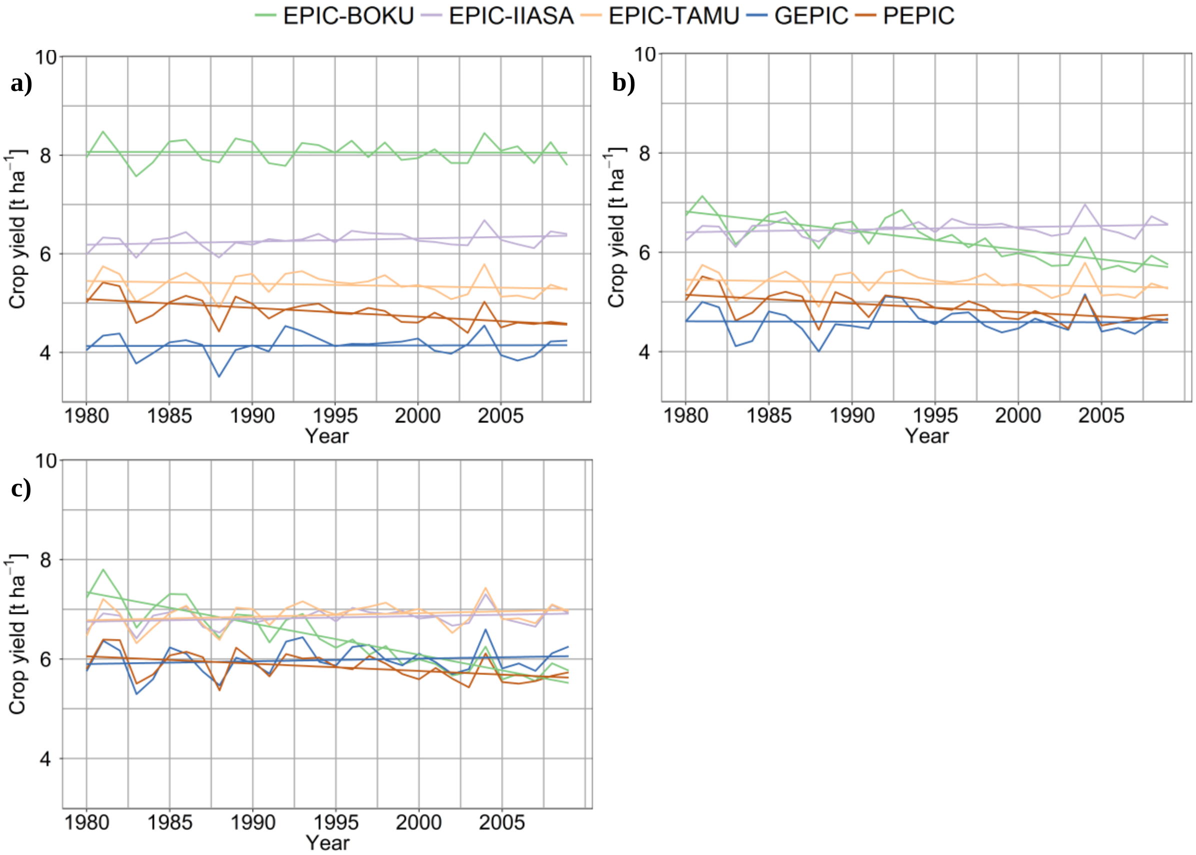

Figure F. Same as Figure 2 in the main manuscript but with linear regressions included but without ensemble mean and reported yields. Regression coefficients are presented in Table F for better readability.

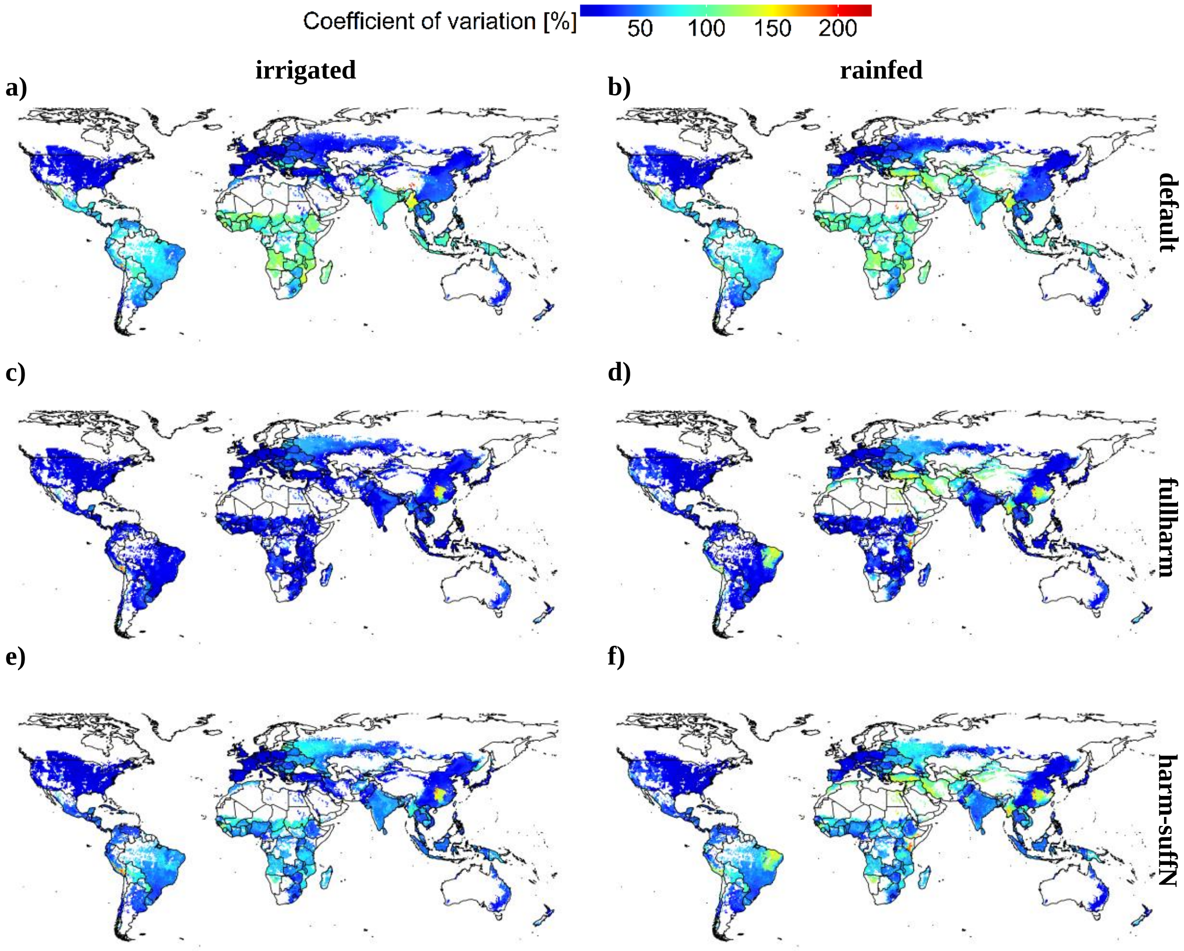

Figure G. Same as Figure 3 in the main body but excluding EPIC-TAMU.

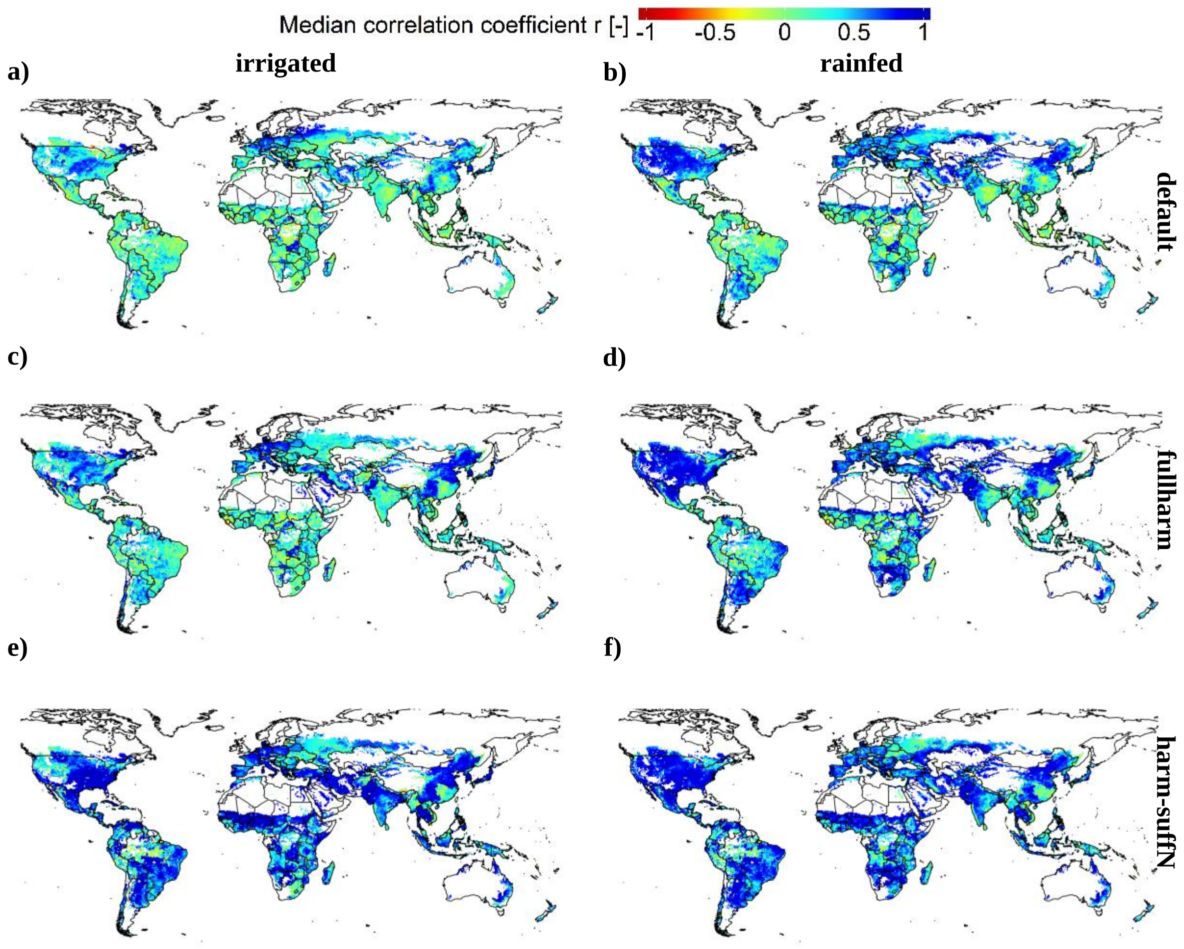

Figure H. Same as Figure 4 in the main body but excluding EPIC-TAMU

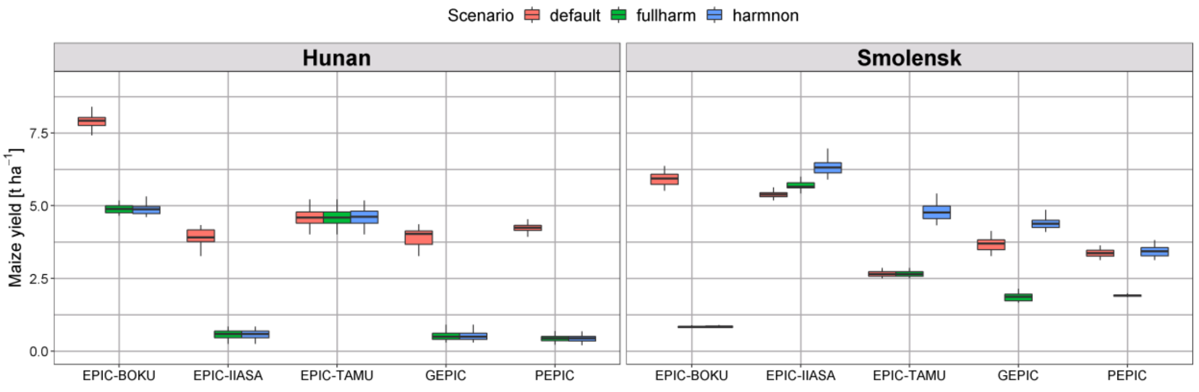

Figure I. Long-term maize yield estimates for the EPIC-based GGCMs in two spatial units at administrative level 2 with increase in CV_{av} after harmonization in Figure 3 of the main body.

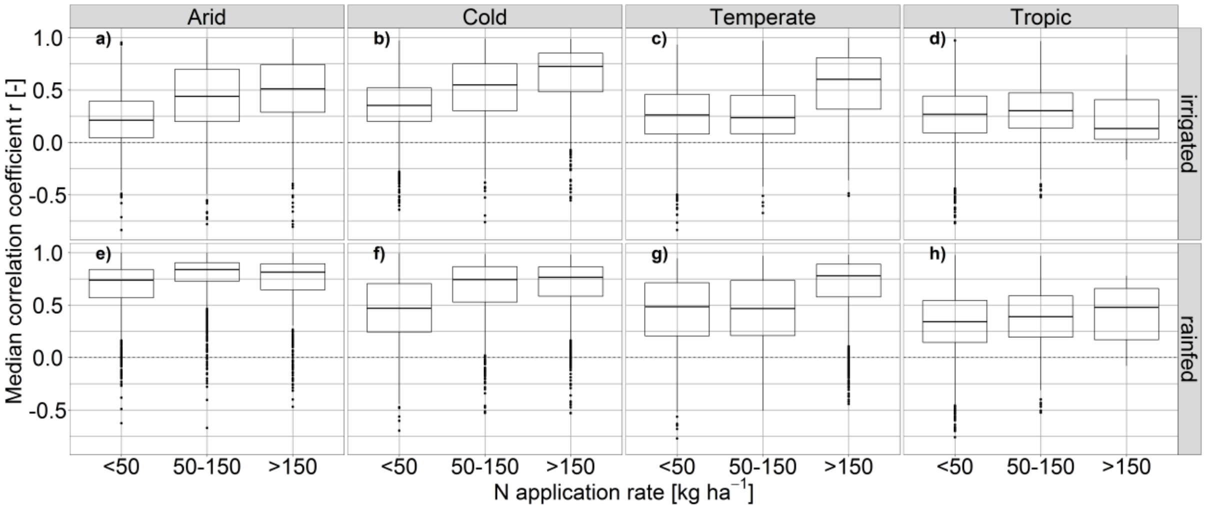

Figure J. Median time-series correlation coefficient r for maize yields among EPIC-based GGCMs compared to binned fertilizer application rates in the fully harmonized management scenario (fullharm) with sufficiently irrigated (a-d) or rainfed (e-h) water supply in each grid cell of four major climate regions.

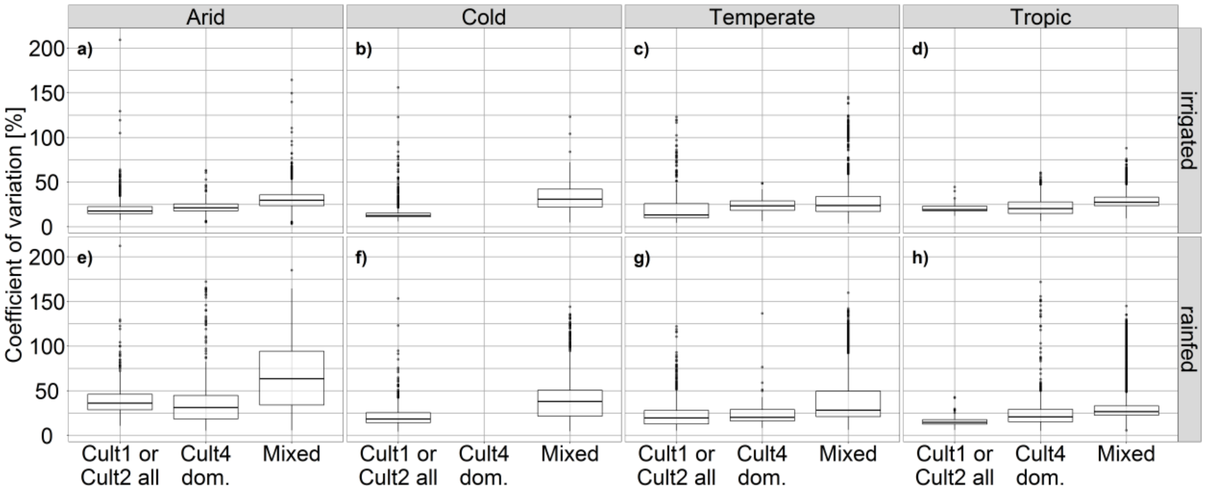

Figure K. Coefficient of variation (CV_{av}) among maize yield estimates in the harm-suffN scenario in grid cells in which either all GGCMs plant the high-yielding cultivars 1 or 2 (Figure 1 in main paper) or in which at least four GGCMs plant the low-yielding drought-sensitive cultivar 4 or in which cultivar types are mostly mixed.

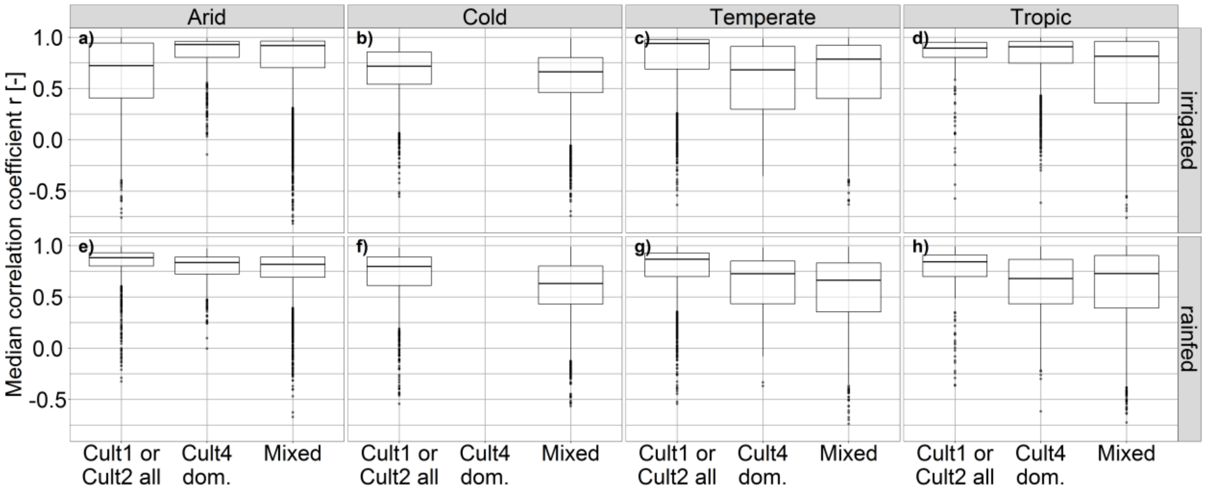

Figure L. Median time-series correlation coefficient in the harm-suffN scenario in grid cells in which either all GGCMs plant the high-yielding cultivars 1 or 2 (Figure 1 in main paper and Table D) or in which at least four GGCMs plant the low-yielding drought-sensitive cultivar 4 or in which cultivar types are mostly mixed.

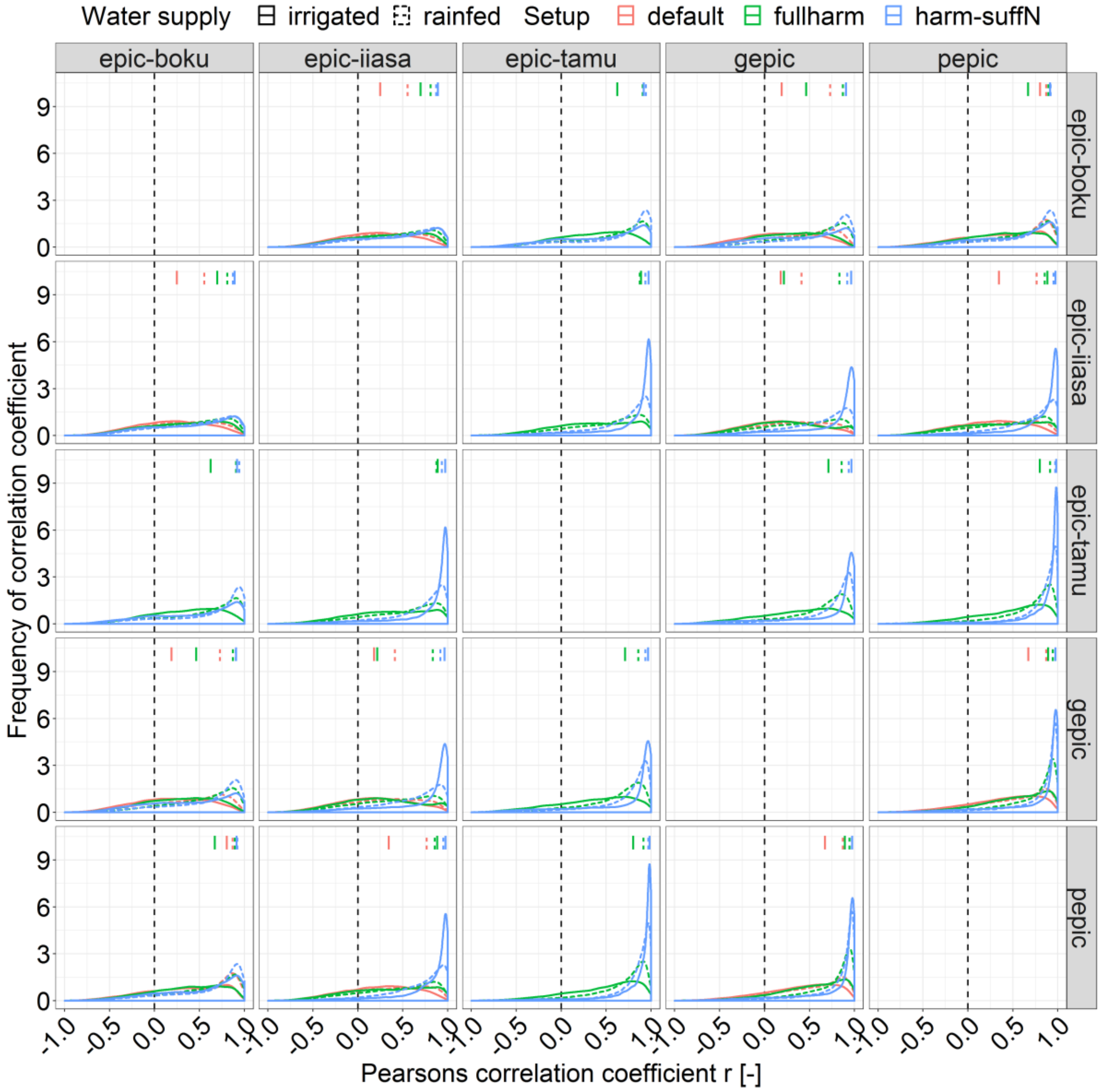

Figure M. Frequency distribution of time-series correlation coefficients among EPIC-based GGCMs in each grid cell and for each management scenario.

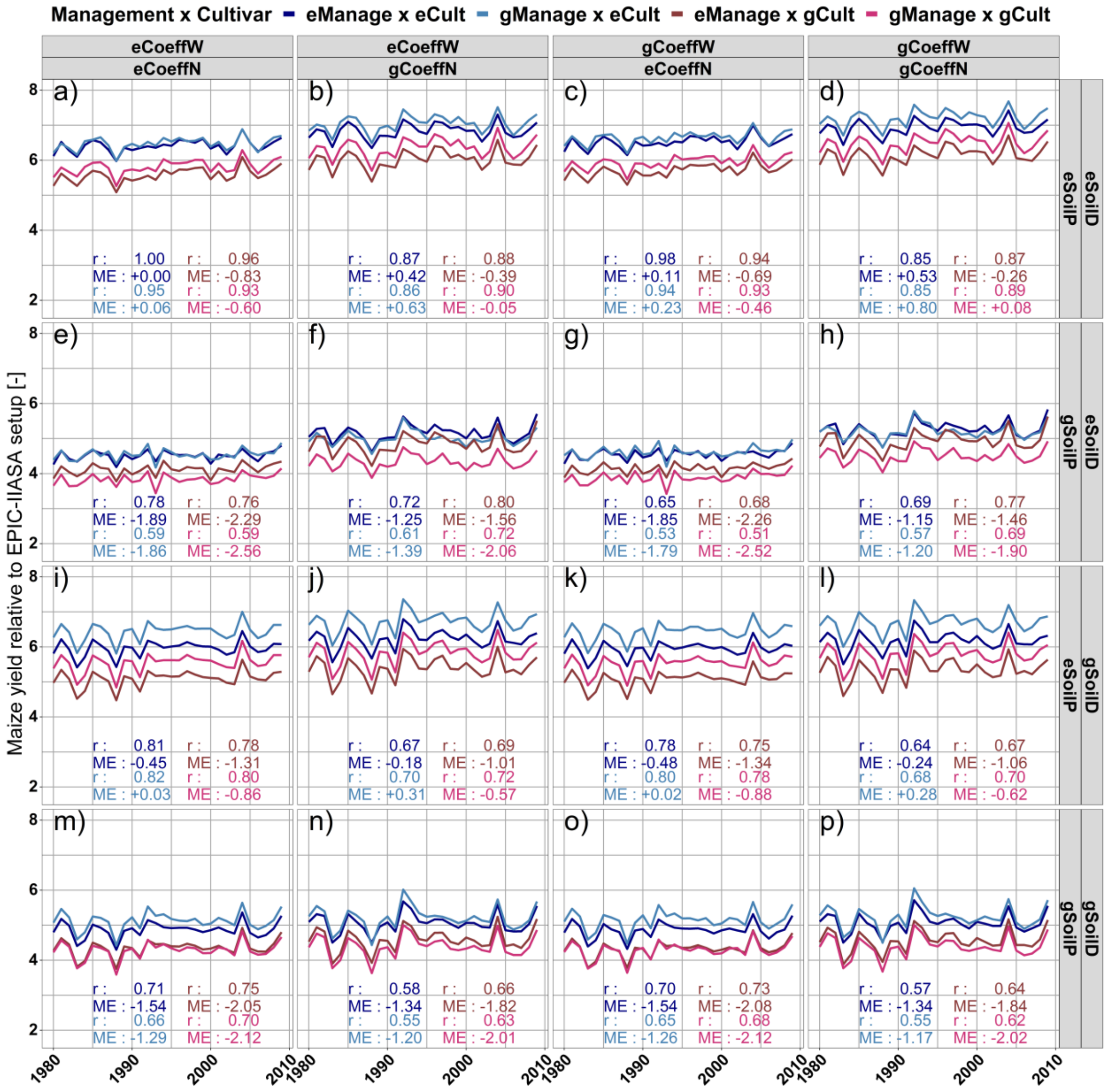

Figure N. Global average rainfed maize yields over a 29 year period for 64 setup combinations based on the EPIC-IIASA and GEPIC setups (Table 3 in main article). e=EPIC-IIASA, g=GEPIC, Cult=cultivar definition and distribution, SoilD=soil data, SoilP=spin-up and soil handling, CoeffN=organic matter and nutrient cycling coefficients, CoeffW=hydrologic coefficients, Manage=crop management.

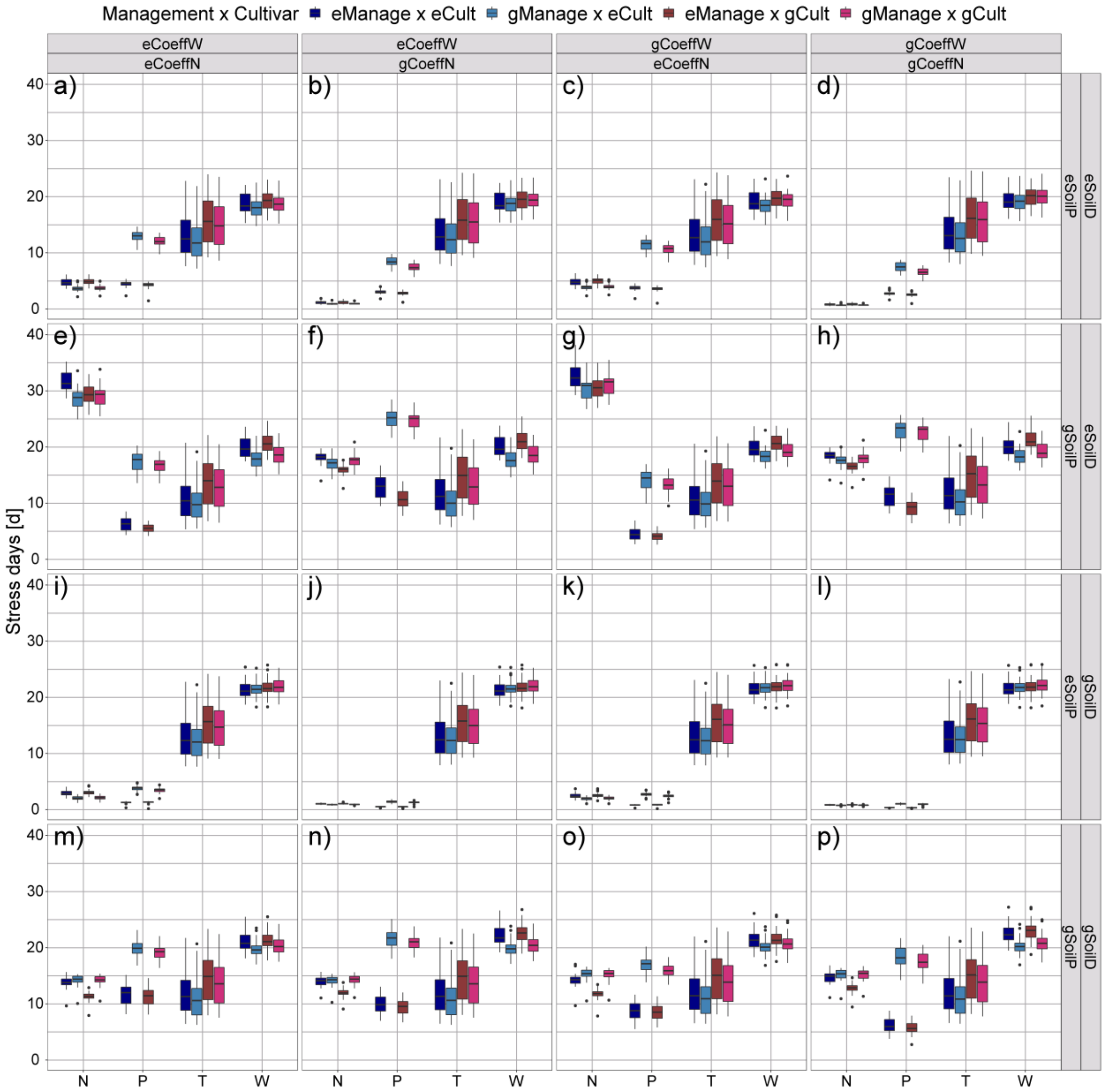

Figure O. Box-and-whisker plots of global averaged growth stresses over a 29 year period for 64 setup combinations based on the EPIC-IIASA and GEPIC setups under rainfed conditions (Table 3 in main paper). e=EPIC-IIASA, g=GEPIC, Cult=cultivar definition and distribution, SoilD=soil data, SoilP=spin-up and soil handling, CoeffN=organic matter and nutrient cycling coefficients, CoeffW=hydrologic coefficients, Manage=crop management. N=nitrogen deficit, P=phosphorus deficit, T=temperature stress, W=water deficit. The corresponding yields are shown in Figure N and their relative difference from the EPIC-IIASA setup in Figure 7 in the main paper.

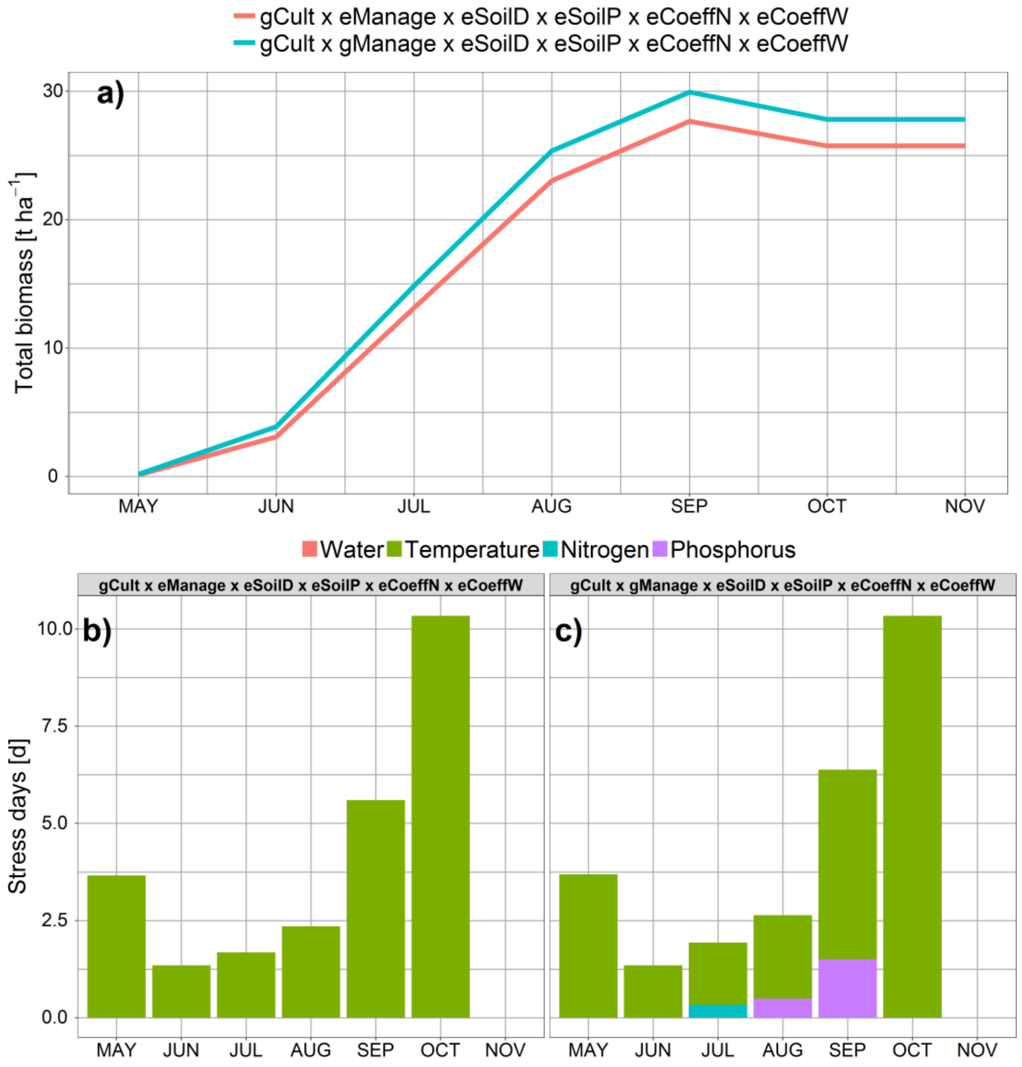

Figure P. (a) Monthly total biomass and (b-c) stress occurrence for a single year in a randomly sampled grid cell of the US Corn Belt differing the managements of EPIC-IIASA and GEPIC (eManage/gManage) with otherwise identical setups (included in Figure 7a of the main article) and a static soil profile (eSoilP). The GEPIC management results in higher biomass at any point of time despite higher stress occurrence (panel c) due to narrower row spacing than in EPIC-IIASA, which increases potential biomass estimation. Nutrient-related stresses occur only for the GEPIC management, which includes plant residue removal after harvest (Table C). This management hence causes a two-fold impact on nutrient stress with overall lower availability of nutrients in the long-run under low-input conditions and higher biomass accumulation - resulting in stronger nutrient mining - early in the growing season.

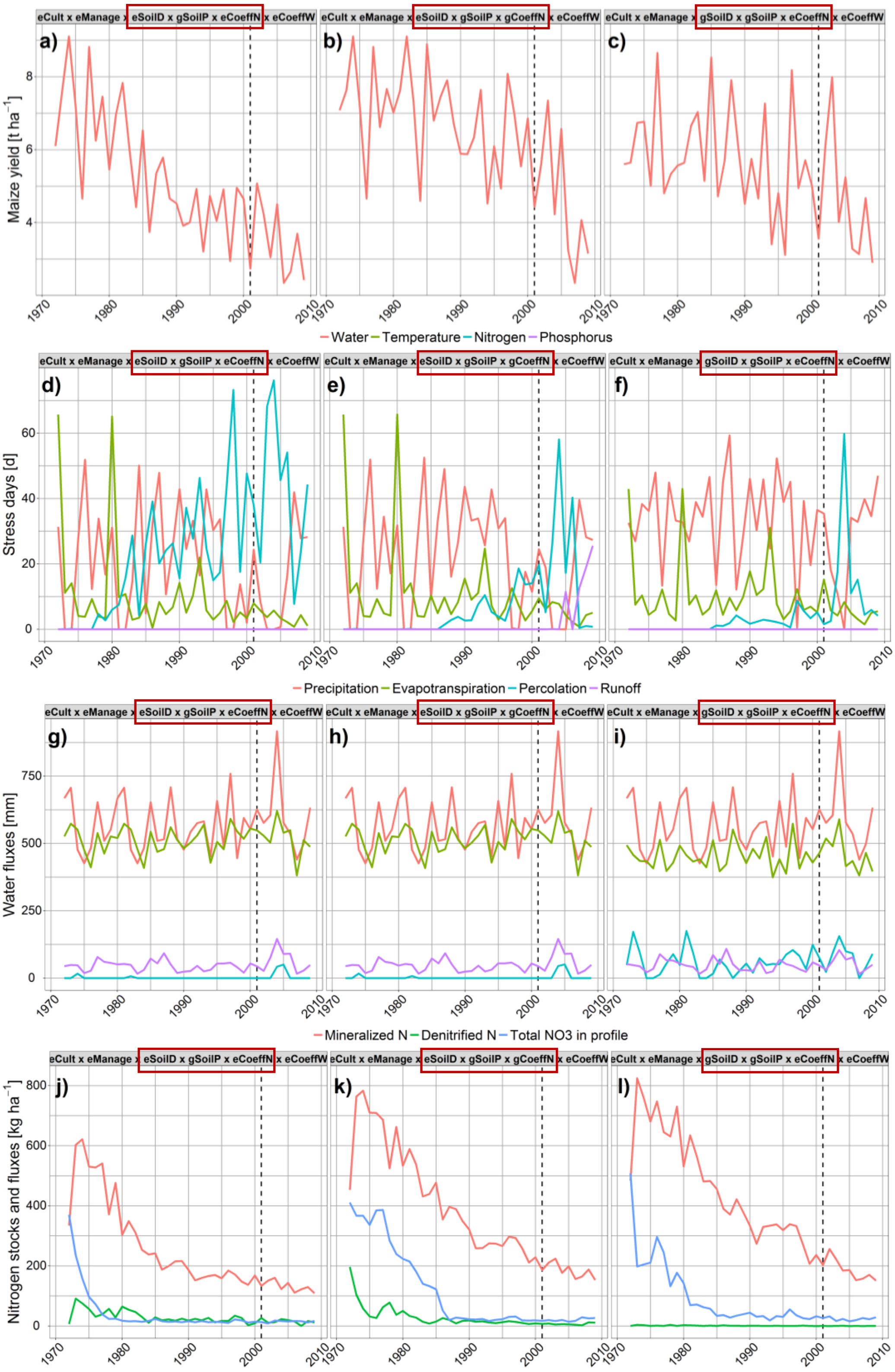

Figure Q. Annual (a-c) yields, (d-f) stresses, (g-i) water fluxes, and (j-l) nitrogen fluxes for a randomly sampled grid in Ukraine with low fertilizer application for three EPIC-GGCM setups differing in soil data (SoilD) and OM/nutrient cycling parameterization (CoeffN) using dynamic soil profile handling (gSoilP). Scenarios correspond to Figure 7e (left column of panels), 7f (center column of panels), and 7m (right column of panels) in the main article. The vertical dashed line indicates the end of the spin-up period (Figure B). The setup in the left column causes low N mineralization and early rapidly increasing N stress, which is lower if gCoeffN (center column) is used due to more rapid turnover of OM. If the soil data of GEPIC are used (right vs. left panel column), water stress is constantly high due to lower field capacity (Figure C), which in turn also triggers earlier the mineralization of OM and causes lower nutrient stresses in the model.

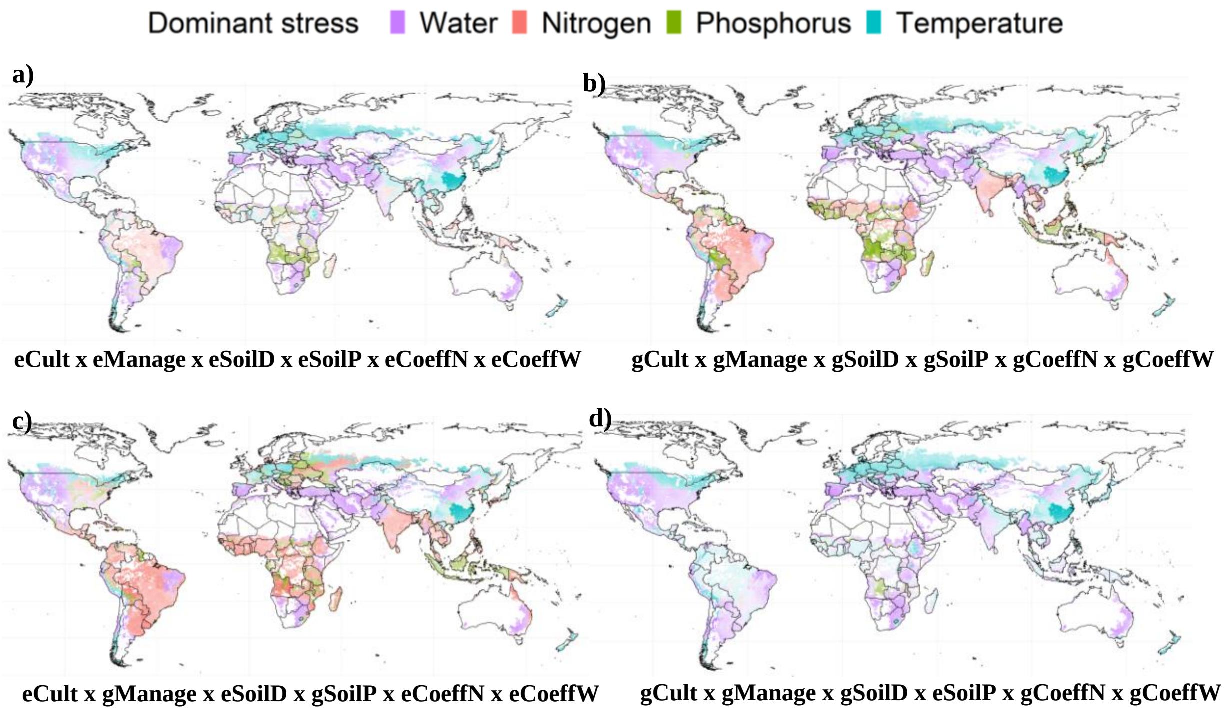

Figure R. Dominant stress per grid cell averaged over the simulation period in four selected setups shown underneath each panel. The transparency of each grid is proportional to the relative magnitude of the stress within the setup. The EPIC-IIASA setup (a) causes a dominance of climate-related stresses in most parts of the world, except for parts of the tropics. With the GEPIC setup (b) nutrient-related stresses are higher and have more coverage throughout the tropics and stretch out to parts of temperate low-input regions while water stress stretches to higher latitudes in the US. The most extensive nutrient stress occurs if the nutrient cycling coefficient of EPIC-IIASA are combined with the dynamic soil handling and management of GEPIC (c). If the GEPIC setup is run with static soil handling of EPIC-IIASA (d), hardly any nutrient-related stresses occur. The corresponding panels in Figure 7 of the main article are a ➔ a, b ➔ p, c ➔ k, d ➔ h.

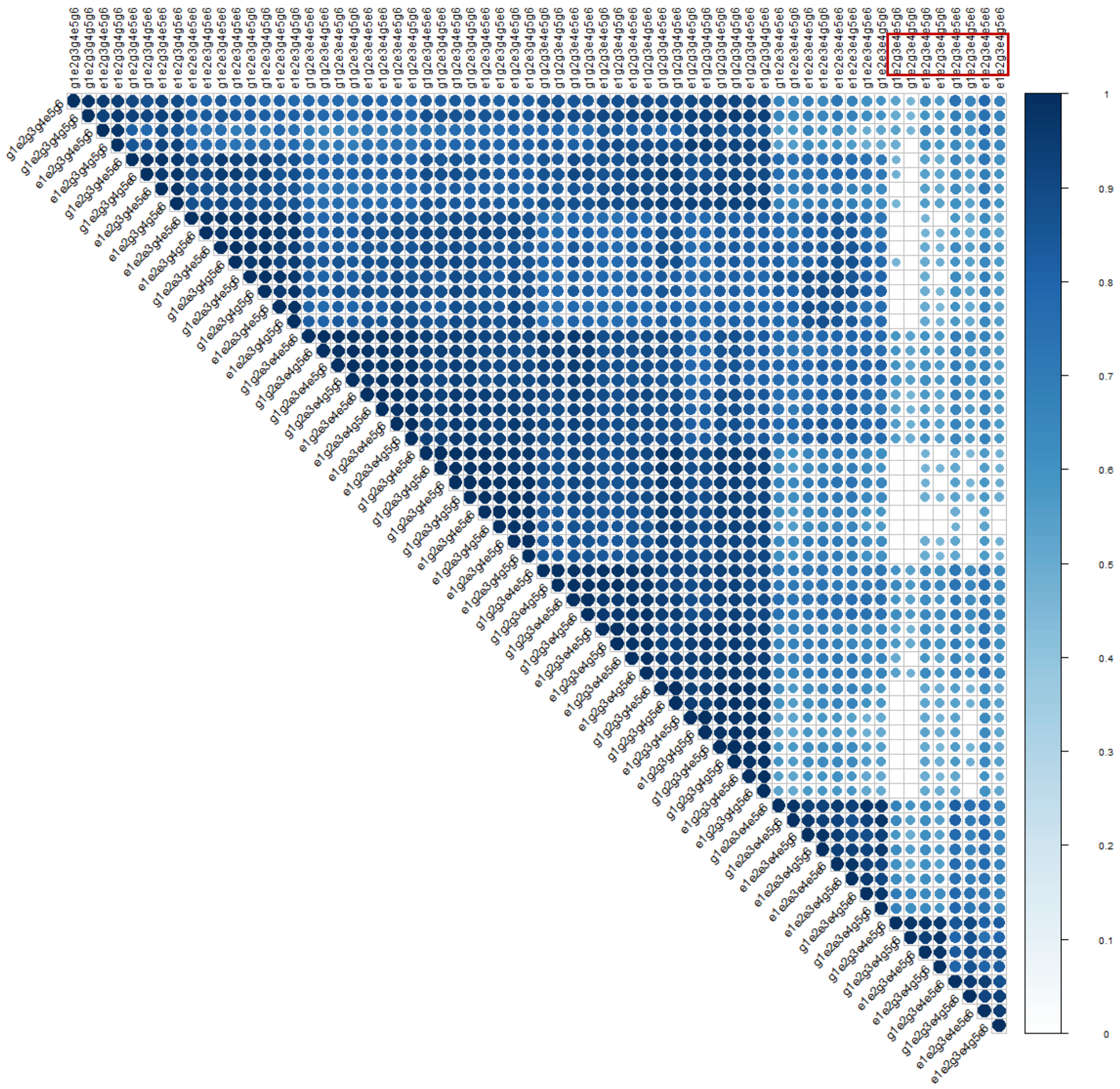

Figure S. Correlation matrix for the 64 setup permutation of EPIC-IIASA and GEPIC. Colour indicates the correlation coefficient r as shown on the right scale, circle sizes represent the level of significance. Insignificant correlations are excluded. The setups are ordered based on hierarchical clustering **[41]**. The red box indicates the setup combinations showing the least agreement with most the majority of other setups. 1= cultivar distribution, 2= soil data, 3=soil handling, 4=nutrient cycling coefficient, 5=hydrologic coefficients, and 5=management. e=EPIC-IIASA and g=GEPIC.

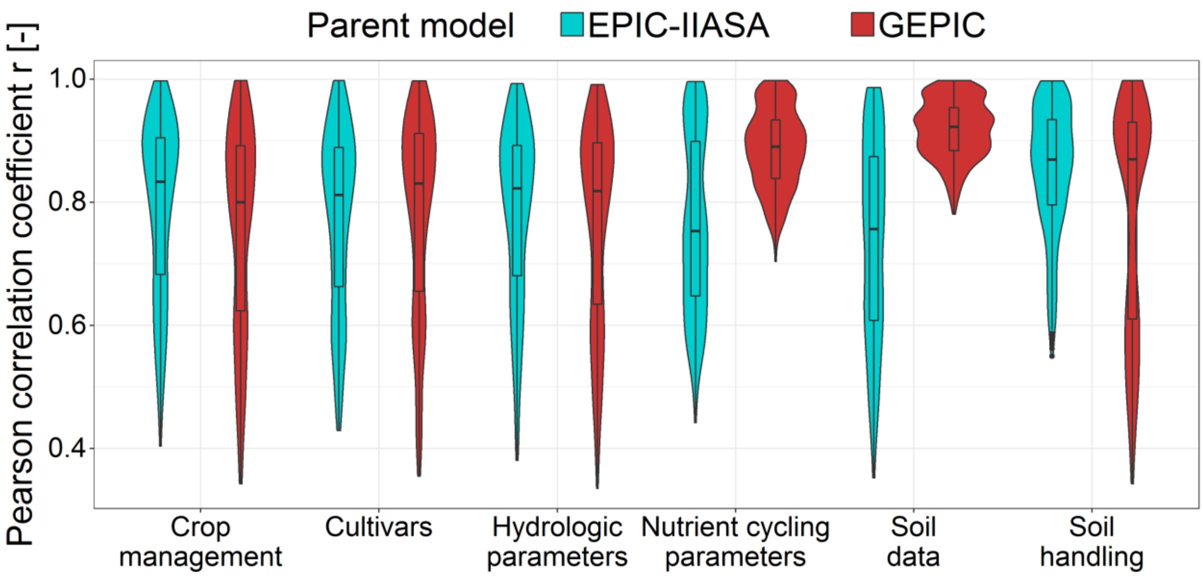

Figure T. Distributions (violins) and box-and-whisker plots of correlation coefficients among all setup combinations of EPIC-IIASA and GEPIC (Table 3 in main article) aggregated by setup domains.

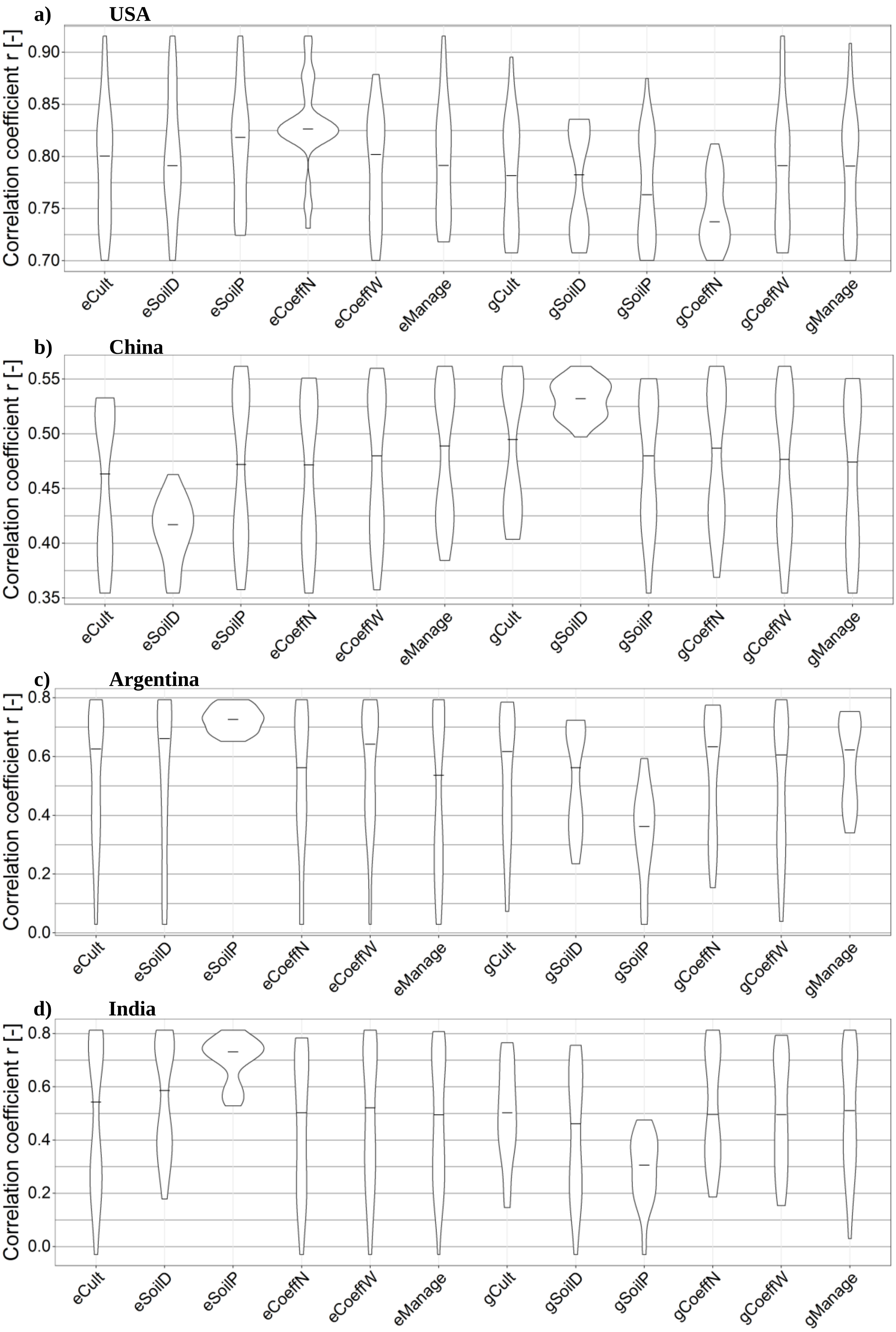

Figure U. Distributions (violins) and medians (horizontal lines) of the time series correlation coefficient r of simulated and reported yields for each setup domain (Table 3 in main article) in (a) USA, (b) China, (c) Argentina, and (d) India. Corresponding performance of each setup combination are shown in Figure 9 of the main article.

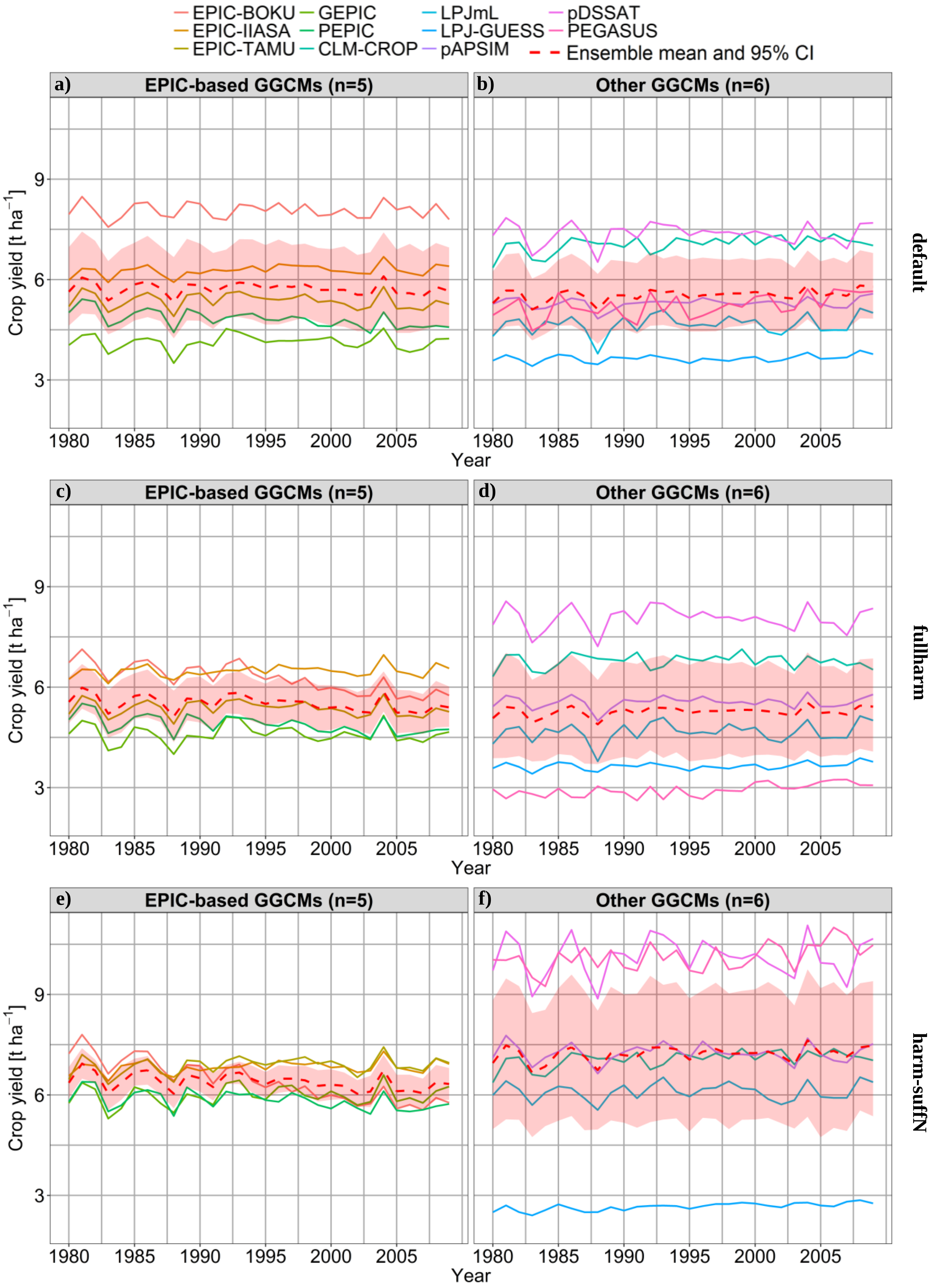

Figure V. Global average area-weighted maize yields and 95% confidence interval of the mean for EPIC-GGCMs and non-EPIC-based GGCMs for three management scenarios. Solid lines show outputs from single models. Table L shows linear regression coefficients and ME relative to FAO reported yields.

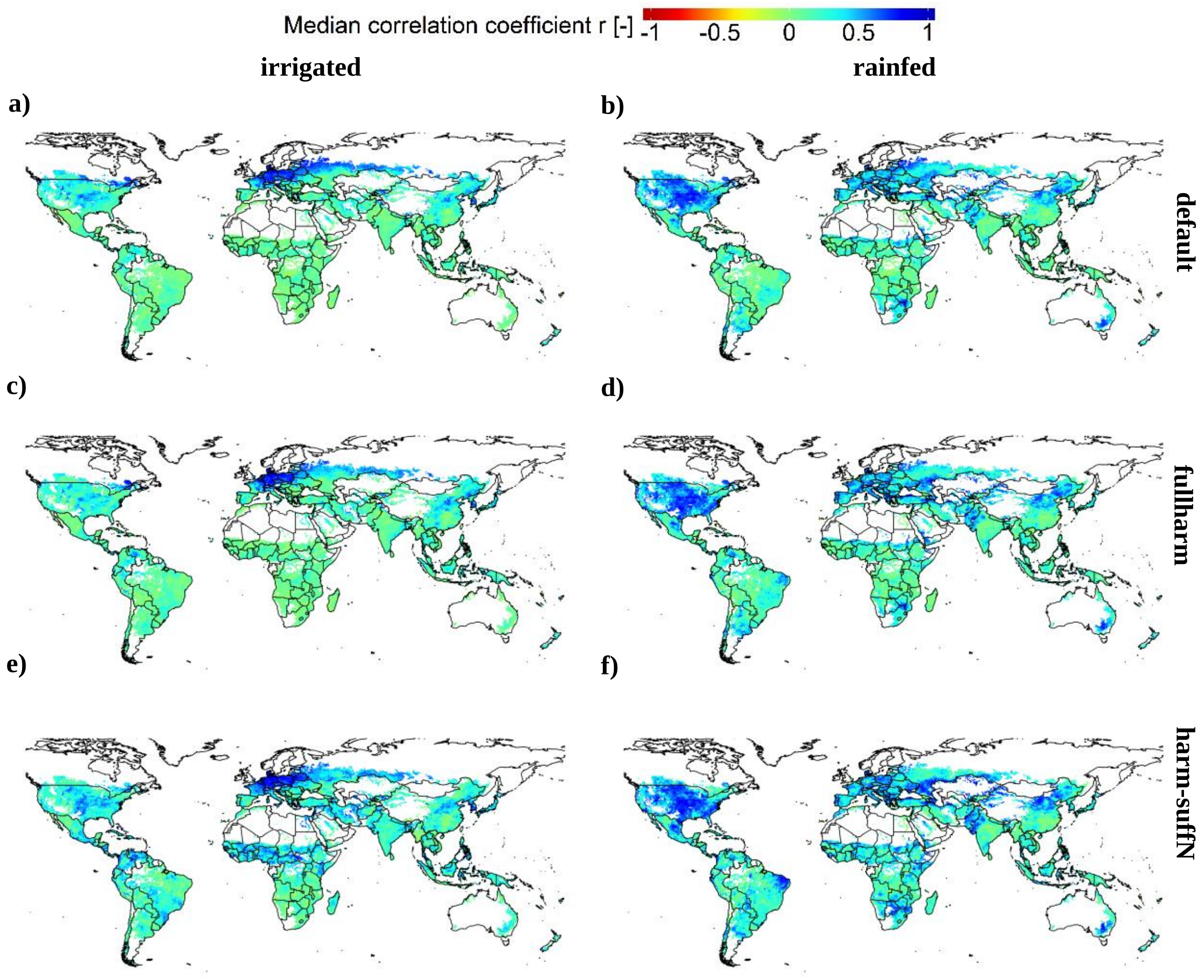

Figure W. Median of time-series correlation coefficient r for maize yield estimates among the whole GGCM ensemble for each of the six crop management scenarios defined in Table 1 of the main article. Evaluations excluding EPIC-TAMU, LPJmL, and LPJ-GUESS, for which default and fullharm are identical, are shown in Figure X.

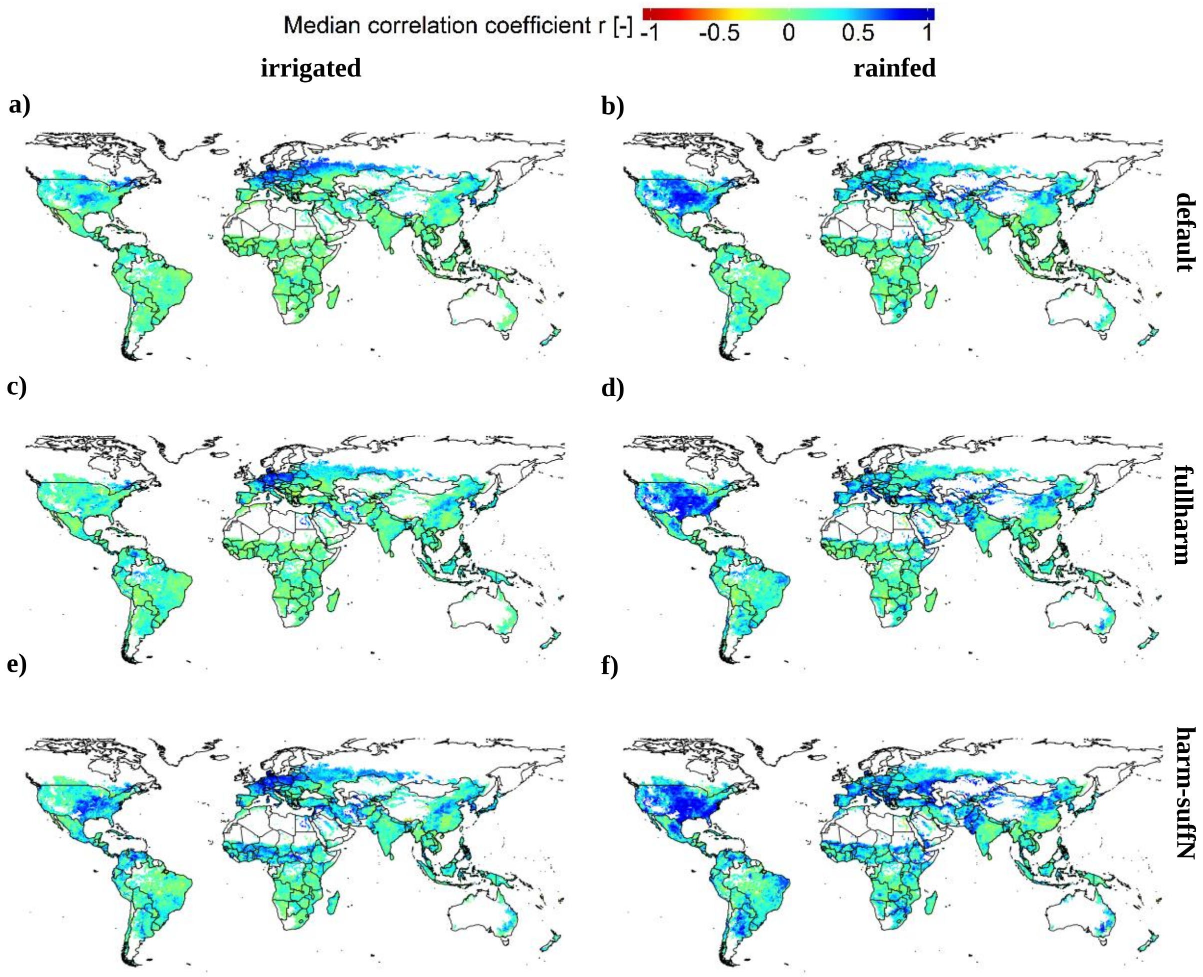

Figure X. Same as Figure W but excluding EPIC-TAMU, LPJmL, and LPJ-GUESS.

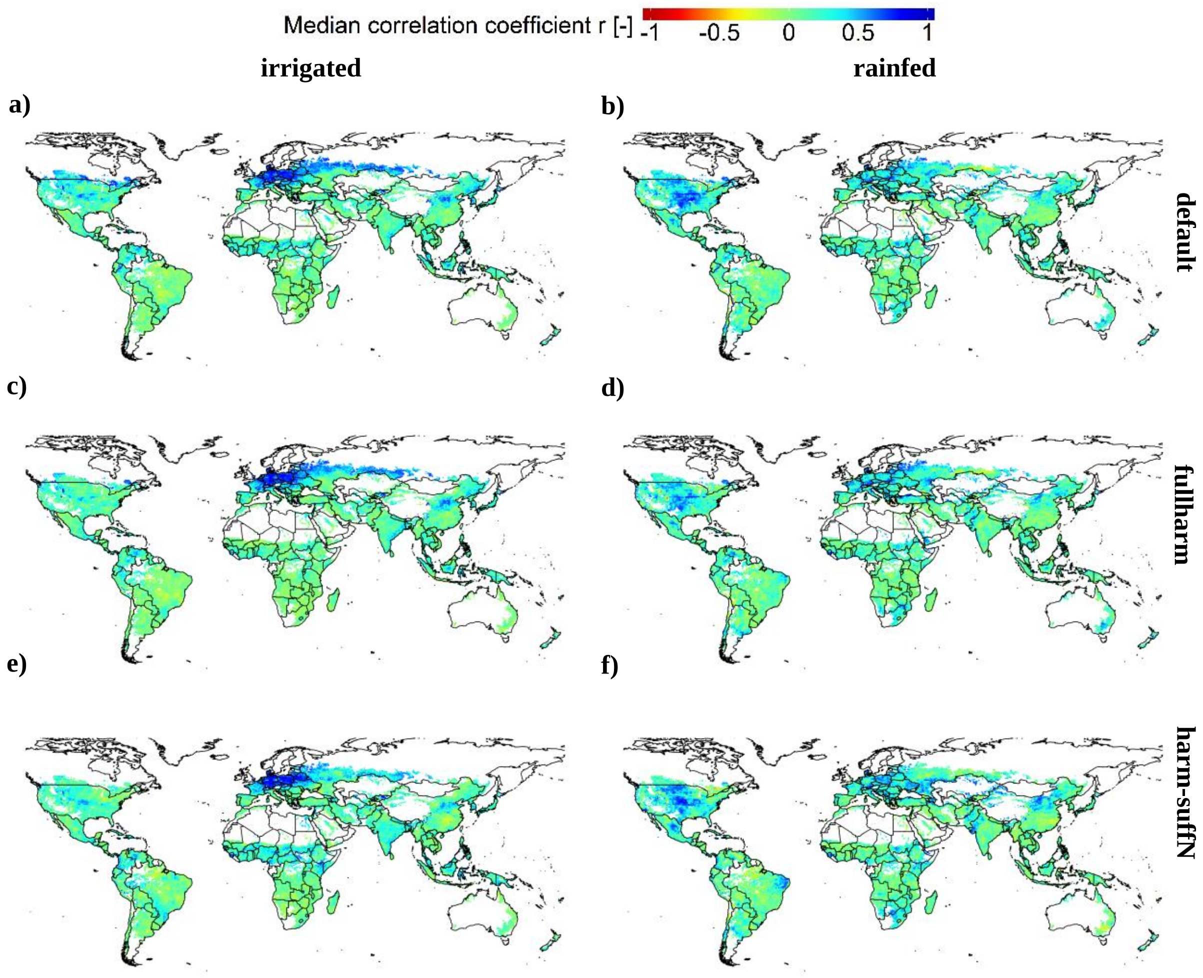

Figure Y. Median of time-series correlation coefficient r for maize yield estimates among the GGCM ensemble excluding the EPIC-based GGCMs for each of the six crop management scenarios defined in Table 1 of the main article. Evaluations excluding LPJmL and LPJ-GUESS, for which default and fullharm are identical, are shown in Figure Z.

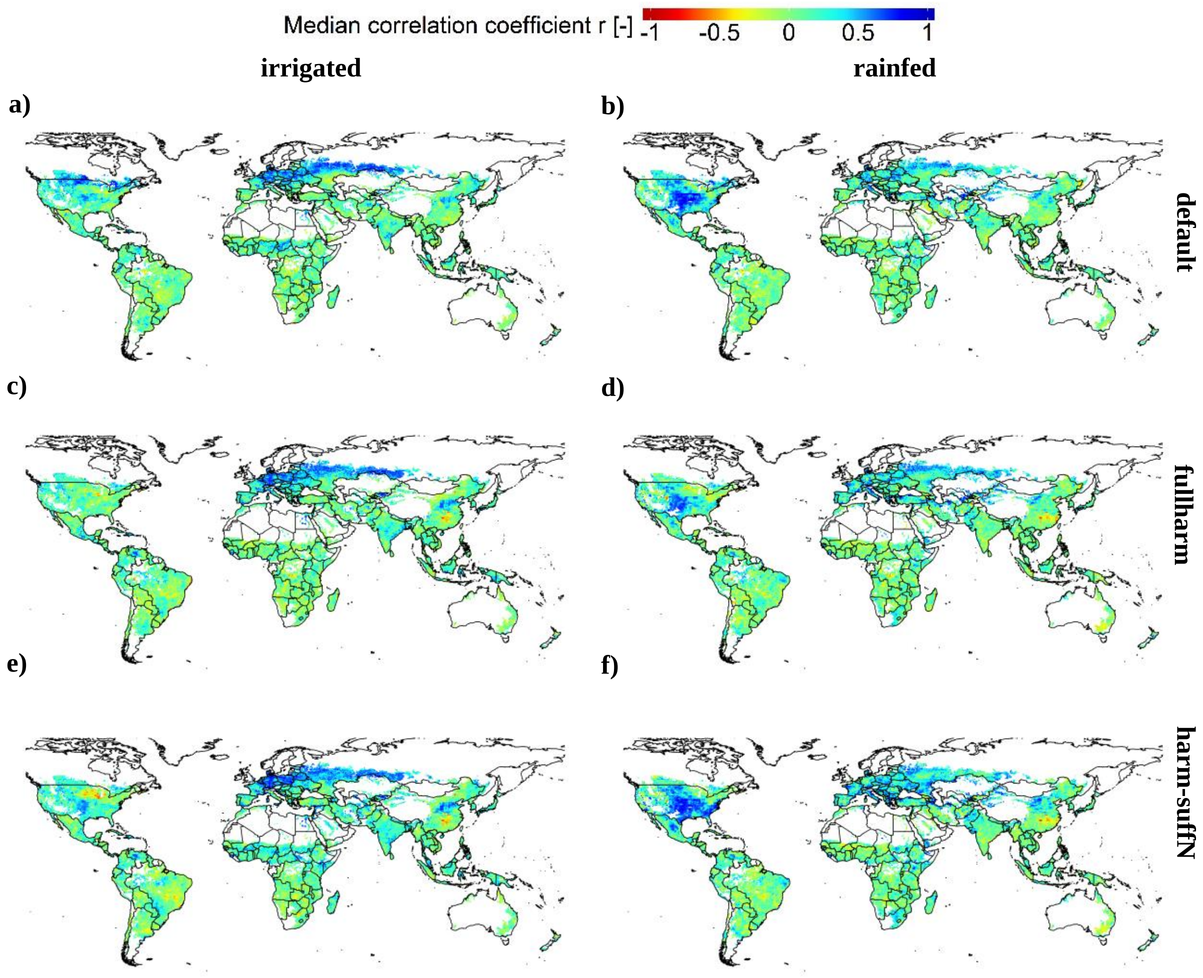

Figure Z. Same as Figure Y but excluding LPJmL and LPJ-GUESS.

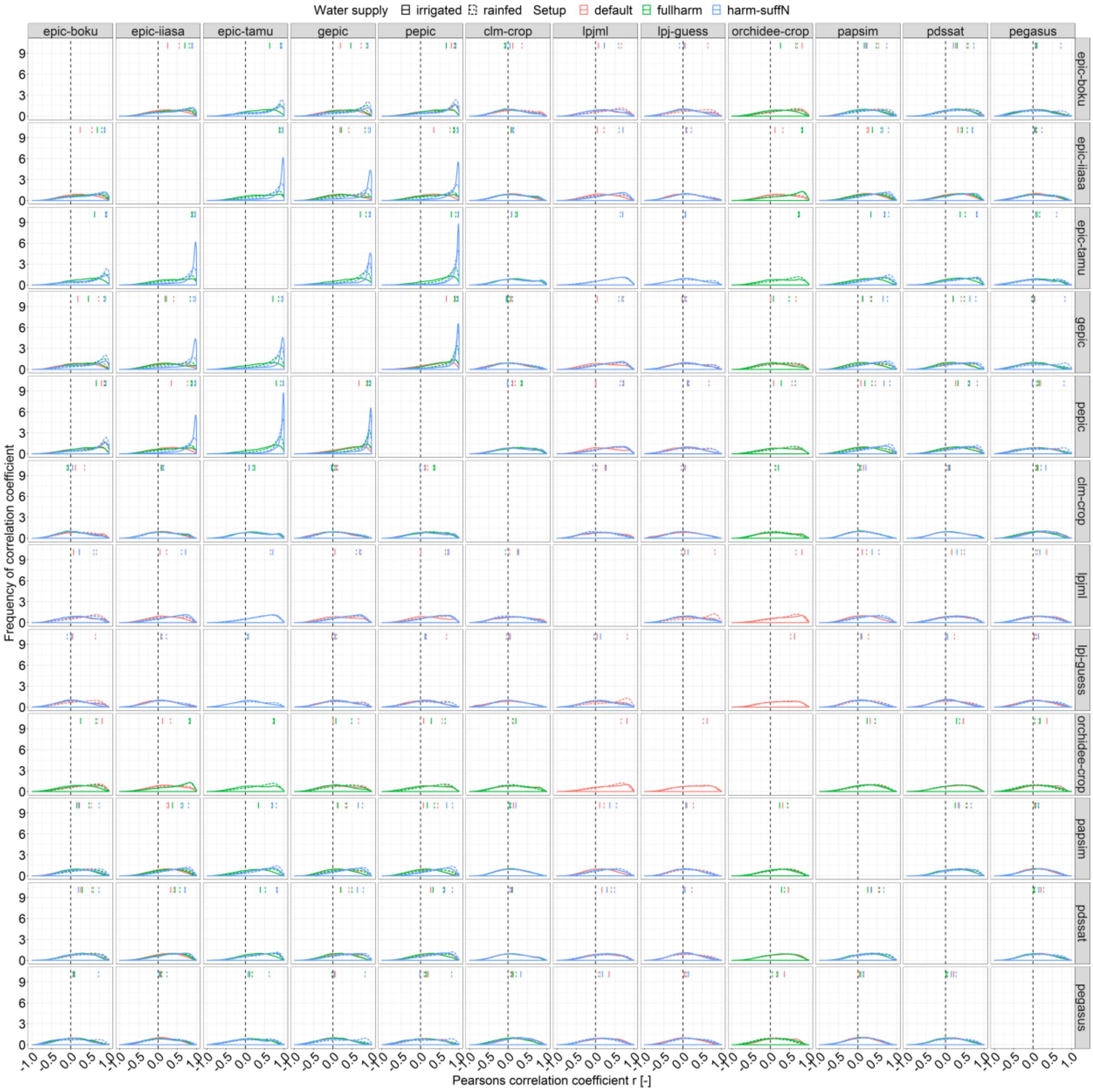

Figure AA. Frequency distributions of time-series correlation coefficients in each grid cell for all GGCMs and setup scenarios (Table 1 in main article). Solid and dashed lines at the top of each panel indicated the location of the major peak in the distribution for rainfed (dashed) or sufficiently irrigated (solid) simulations of each management scenario (Table 1 in main article).

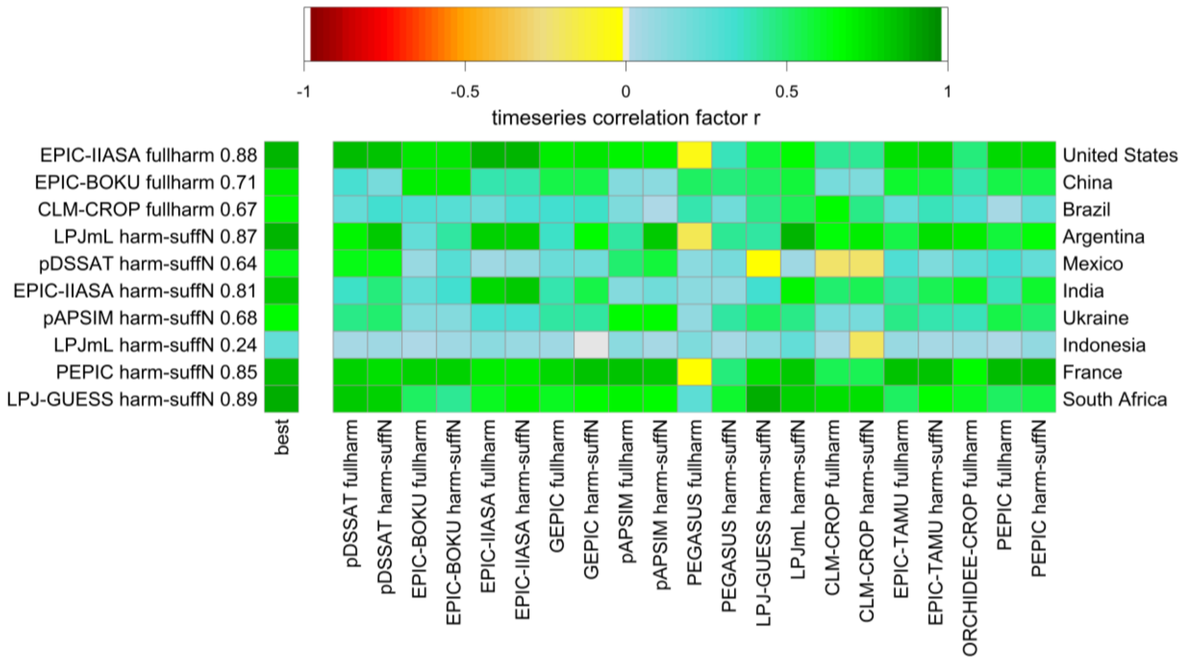

Figure AB. Time-series correlation coefficients for all GGCMs with the fullharm and harm-suffN scenarios (x-axis) in the top ten maize producing countries (right y-axis) and the best performing GGCM/setup combination including the r value (left y-axis).

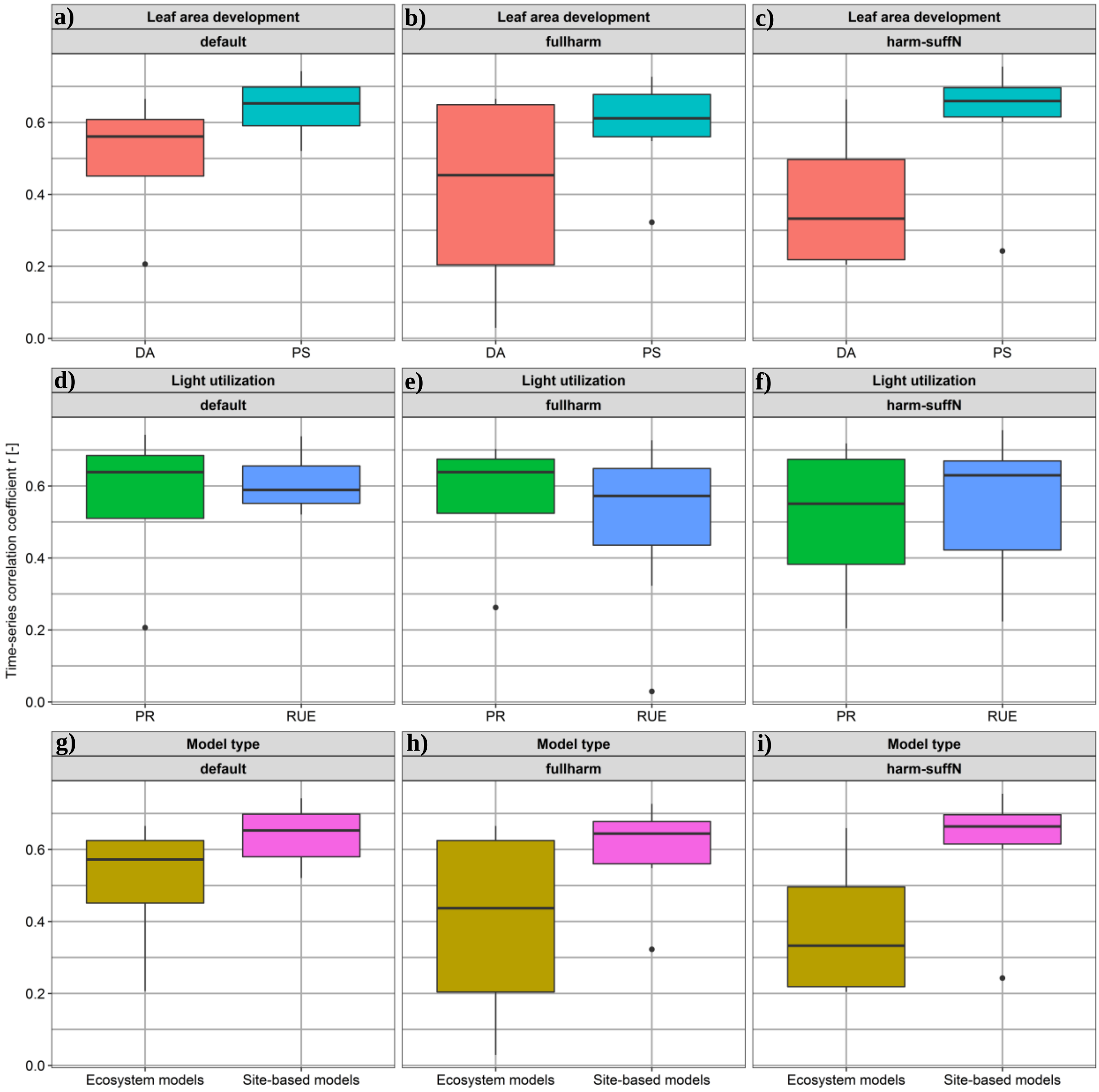

Figure AC. Time-series correlation coefficients for GGCMs grouped by basic characteristics (Table A) for the three setup scenarios. DA=dynamic leaf area development, PS=prescribed leaf area development, PR=gross photosynthesis-respiration light utilisation approach, RUE=radiation use efficiency light utilisation approach.

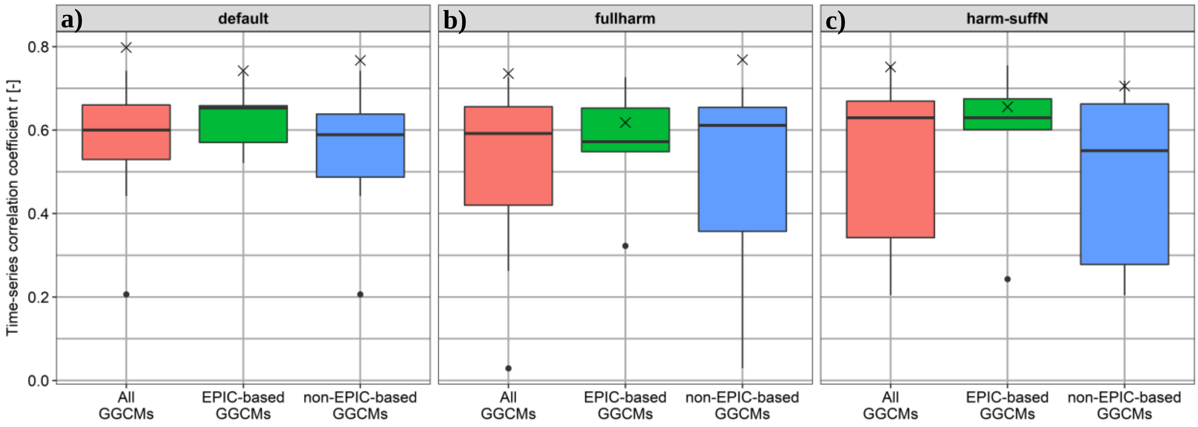

Figure AD. Box-and-whisker plots of time-series correlation coefficients for single GGCMs against FOASTAT global reported yields grouped into the (sub-)ensembles "All GGCMs", "EPIC-based GGCMs", and "non-EPIC-based GGCMs" for each setup scenario (a-c). Cross marks show time-series correlation coefficients of the multi-GGCM mean for each ensemble.

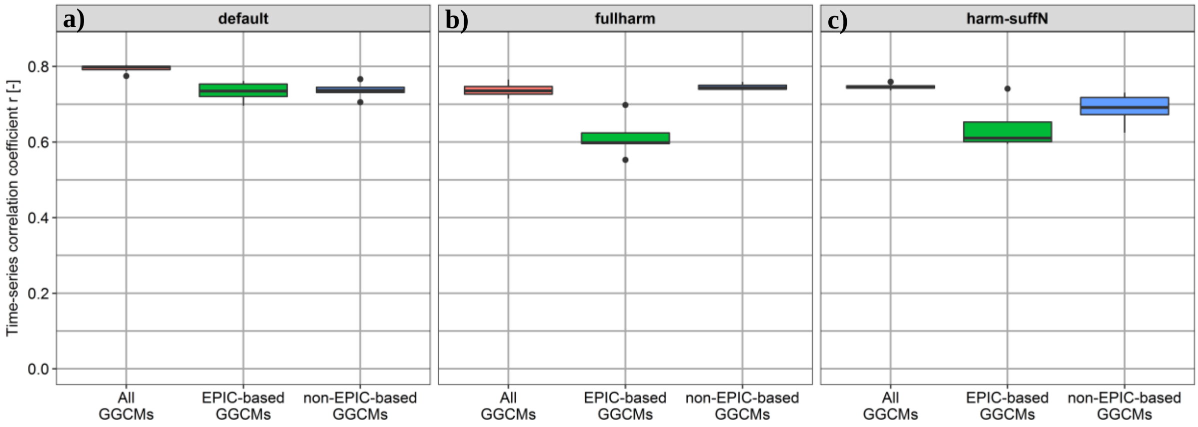

Figure AE. Box-and-whisker plots of time-series correlation coefficients for permutations of multi-GGCM means excluding one GGCM at a time against FOASTAT global reported yields for each setup scenario (a-c). GGCMs are grouped into the (sub-)ensembles "All GGCMs", "EPIC-based GGCMs", and "non-EPIC-based GGCMs" as in Figure AD.

References

1. Izaurralde RC, Williams JR, McGill WB, Rosenberg NJ, Jakas MCQ. Simulating soil C dynamics with EPIC: Model description and testing against long-term data. Ecological Modelling. 2006;192: 362–384. doi:10.1016/j.ecolmodel.2005.07.010

2. Izaurralde RC, McGill WB, Williams JR. Development and application of the EPIC model for carbon cycle, greenhouse gas mitigation, and biofuel studies. Managing Agricultural Greenhouse Gases. Elsevier; 2012. pp. 293–308.

3. Liu W, Yang H, Folberth C, Wang X, Luo Q, Schulin R. Global investigation of impacts of PET methods on simulating crop-water relations for maize. Agricultural and Forest Meteorology. 2016;221: 164–175. doi:10.1016/j.agrformet.2016.02.017

4. Parton WJ, Ojima DS, Cole CV, Schimel DS. A General Model for Soil Organic Matter Dynamics: Sensitivity to Litter Chemistry, Texture and Management. Quantitative Modeling of Soil Forming Processes. 1994;sssaspecialpubl: 147–167. doi:10.2136/sssaspecpub39.c9

5. Seligman NG, Keulen HV. 4.10 PAPRAN: A simulation model of annual pasture production limited by rainfall and nitrogen. Simulation of nitrogen behaviour of soil-plant systems. 1980;192.

6. Havlík P, Schneider UA, Schmid E, Böttcher H, Fritz S, Skalský R, et al. Global land-use implications of first and second generation biofuel targets. Energy Policy. 2011;39: 5690–5702. doi:10.1016/j.enpol.2010.03.030

7. Schneider UA, Havlík P, Schmid E, Valin H, Mosnier A, Obersteiner M, et al. Impacts of population growth, economic development, and technical change on global food production and consumption. Agricultural Systems. 2011;104: 204–215. doi:10.1016/j.agsy.2010.11.003

8. Nelson GC, Valin H, Sands RD, Havlík P, Ahammad H, Deryng D, et al. Climate change effects on agriculture: Economic responses to biophysical shocks. PNAS. 2014;111: 3274–3279. doi:10.1073/pnas.1222465110

9. Frank S, Schmid E, Havlík P, Schneider UA, Böttcher H, Balkovič J, et al. The dynamic soil organic carbon mitigation potential of European cropland. Global Environmental Change. 2015;35: 269–278. doi:10.1016/j.gloenvcha.2015.08.004

10. Stolbovoy V, Montanarella L, Panagos P. Carbon Sink Enhancement in Soils of Europe: Data, Modeling, Verification. JRC technical and scientific reports, EUR. 2007;23037.

11. Skalský R, Tarasovičová Z, Balkovič J, Schmid E, Fuchs M, Moltchanova E, et al. GEO-BENE global database for bio-physical modeling. GEOBENE project. 2008;

12. Elshout PMF, Zelm R van, Balkovic J, Obersteiner M, Schmid E, Skalsky R, et al. Greenhouse-gas payback times for crop-based biofuels. Nature Climate Change. 2015;5: 604–610. doi:10.1038/nclimate2642

13. Sacks WJ, Deryng D, Foley JA, Ramankutty N. Crop planting dates: an analysis of global patterns. Global Ecology and Biogeography. 2010;19: 607–620. doi:10.1111/j.1466-8238.2010.00551.x

14. Mueller ND, Gerber JS, Johnston M, Ray DK, Ramankutty N, Foley JA. Closing yield gaps through nutrient and water management. Nature. 2012;490: 254–257. doi:10.1038/nature11420

15. Balkovič J, van der Velde M, Schmid E, Skalský R, Khabarov N, Obersteiner M, et al. Pan-European crop modelling with EPIC: Implementation, up-scaling and regional crop yield validation. Agricultural Systems. 2013;120: 61–75. doi:10.1016/j.agsy.2013.05.008

16. Xiong W, Balkovič J, van der Velde M, Zhang X, Izaurralde RC, Skalský R, et al. A calibration procedure to improve global rice yield simulations with EPIC. Ecological Modelling. 2014;273: 128–139. doi:10.1016/j.ecolmodel.2013.10.026

17. Xiong W, van der Velde M, Holman IP, Balkovic J, Lin E, Skalský R, et al. Can climate-smart agriculture reverse the recent slowing of rice yield growth in China? Agriculture, Ecosystems & Environment. 2014;196: 125–136. doi:10.1016/j.agee.2014.06.014

18. Balkovič J, van der Velde M, Skalský R, Xiong W, Folberth C, Khabarov N, et al. Global wheat production potentials and management flexibility under the representative concentration pathways. Global and Planetary Change. 2014;122: 107–121. doi:10.1016/j.gloplacha.2014.08.010

19. Xiong W, Skalský R, Porter CH, Balkovič J, Jones JW, Yang D. Calibration-induced uncertainty of the EPIC model to estimate climate change impact on global maize yield. Journal of Advances in Modeling Earth Systems. 2016;8: 1358–1375. doi:10.1002/2016MS000625

20. McGill WB. PHOENIX, a model of the dynamics of carbon and nitrogen in grassland soil. Terrestrial nitrogen cycles. 1981;

21. Lychuk TE, Izaurralde RC, Hill RL, McGill WB, Williams JR. Biochar as a global change adaptation: predicting biochar impacts on crop productivity and soil quality for a tropical soil with the Environmental Policy Integrated Climate (EPIC) model. Mitig Adapt Strateg Glob Change. 2015;20: 1437–1458. doi:10.1007/s11027-014-9554-7

22. Gelfand I, Sahajpal R, Zhang X, Izaurralde RC, Gross KL, Robertson GP. Sustainable bioenergy production from marginal lands in the US Midwest. Nature. 2013;493: 514–517. doi:10.1038/nature11811

23. Zhang X, Izaurralde RC, Manowitz DH, Sahajpal R, West TO, Thomson AM, et al. Regional scale cropland carbon budgets: Evaluating a geospatial agricultural modeling system using inventory data. Environmental Modelling & Software. 2015;63: 199–216. doi:10.1016/j.envsoft.2014.10.005

24. Liu J, Williams JR, Zehnder AJB, Yang H. GEPIC – modelling wheat yield and crop water productivity with high resolution on a global scale. Agricultural Systems. 2007;94: 478–493. doi:10.1016/j.agsy.2006.11.019

25. Folberth C, Gaiser T, Abbaspour KC, Schulin R, Yang H. Regionalization of a large-scale crop growth model for sub-Saharan Africa: Model setup, evaluation, and estimation of maize yields. Agriculture, Ecosystems & Environment. 2012;151: 21–33. doi:10.1016/j.agee.2012.01.026

26. FAO. Fertistat, Fertilizer Use Statistics. 2007;

27. van der Velde M, Folberth C, Balkovič J, Ciais P, Fritz S, Janssens IA, et al. African crop yield reductions due to increasingly unbalanced Nitrogen and Phosphorus consumption. Global Change Biology. 2014;20: 1278–1288. doi:10.1111/gcb.12481

28. Folberth C, Yang H, Gaiser T, Liu J, Wang X, Williams J, et al. Effects of ecological and conventional agricultural intensification practices on maize yields in sub-Saharan Africa under potential climate change. Environ Res Lett. 2014;9: 044004. doi:10.1088/1748-9326/9/4/044004

29. Liu W, Yang H, Liu J, Azevedo LB, Wang X, Xu Z, et al. Global assessment of nitrogen losses and trade-offs with yields from major crop cultivations. Science of The Total Environment. 2016;572: 526–537. doi:10.1016/j.scitotenv.2016.08.093

30. Deryng D, Sacks WJ, Barford CC, Ramankutty N. Simulating the effects of climate and agricultural management practices on global crop yield. Global Biogeochemical Cycles. 2011;25. doi:10.1029/2009GB003765

31. Fader M, Rost S, Müller C, Bondeau A, Gerten D. Virtual water content of temperate cereals and maize: Present and potential future patterns. Journal of Hydrology. 2010;384: 218–231. doi:10.1016/j.jhydrol.2009.12.011

32. Bloh W von, Schaphoff S, Müller C, Rolinski S, Waha K, Zaehle S. Implementing the nitrogen cycle into the dynamic global vegetation, hydrology, and crop growth model LPJmL (version 5.0). Geoscientific Model Development. 2018;11: 2789–2812. doi:https://doi.org/10.5194/gmd-11-2789-2018

33. Olin S, Schurgers G, Lindeskog M, Wårlind D, Smith B, Bodin P, et al. Modelling the response of yields and tissue C : N to changes in atmospheric CO_2 and N management in the main wheat regions of western Europe. Biogeosciences. 2015;12: 2489–2515. doi:https://doi.org/10.5194/bg-12-2489-2015

34. Bassu S, Brisson N, Durand J-L, Boote K, Lizaso J, Jones JW, et al. How do various maize crop models vary in their responses to climate change factors? Global Change Biology. 2014;20: 2301–2320. doi:10.1111/gcb.12520

35. Wang E, Martre P, Zhao Z, Ewert F, Maiorano A, Rötter RP, et al. The uncertainty of crop yield projections is reduced by improved temperature response functions. Nature Plants. 2017;3: 17102. doi:10.1038/nplants.2017.102

36. Müller C, Elliott J, Chryssanthacopoulos J, Arneth A, Balkovic J, Ciais P, et al. Global gridded crop model evaluation: benchmarking, skills, deficiencies and implications. Geosci Model Dev. 2017;10: 1403–1422. doi:10.5194/gmd-10-1403-2017

37. Cabelguenne M, Debaeke P, Bouniols A. EPICphase, a version of the EPIC model simulating the effects of water and nitrogen stress on biomass and yield, taking account of developmental stages: validation on

maize, sunflower, sorghum, soybean and winter wheat. Agricultural Systems. 1999;60: 175–196. doi:10.1016/S0308-521X(99)00027-X

38. Gaiser T, de Barros I, Sereke F, Lange F-M. Validation and reliability of the EPIC model to simulate maize production in small-holder farming systems in tropical sub-humid West Africa and semi-arid Brazil. Agriculture, Ecosystems & Environment. 2010;135: 318–327. doi:10.1016/j.agee.2009.10.014

39. Peel MC, Finlayson BL, McMahon TA. Updated world map of the Köppen-Geiger climate classification. Hydrol Earth Syst Sci. 2007;11: 1633–1644. doi:10.5194/hess-11-1633-2007

40. Elliott J, Müller C, Deryng D, Chryssanthacopoulos J, Boote KJ, Büchner M, et al. The Global Gridded Crop Model Intercomparison: data and modeling protocols for Phase 1 (v1.0). Geosci Model Dev. 2015;8: 261–277. doi:10.5194/gmd-8-261-2015

41. Wei T, Simko V. corrplot: Visualization of a correlation matrix. R package version 073. 2013;230: 11.
